# Worldwide patterns and trends in childhood and adolescent brain cancers, 1990–2021: insights from the global burden of disease study 2021

**DOI:** 10.3389/fpubh.2025.1591309

**Published:** 2025-09-12

**Authors:** Linbo Li, Chunhai Cai, Qianqian Zhao, Chunsun Fan, Jian Fan

**Affiliations:** ^1^Central Laboratory, Qidong People's Hospital, Qidong Liver Cancer Institute, Affiliated Qidong Hospital of Nantong University, Qidong, Jiangsu, China; ^2^State Key Laboratory of Cardiology and Medical Innovation Center, Shanghai East Hospital, Frontier Science Center for Stem Cell Research, School of Life Sciences and Technology, Tongji University, Shanghai, China

**Keywords:** pediatric brain cancer, global burden of disease, health inequity, sociodemographic index, joinpoint regression

## Abstract

**Background:**

Childhood and adolescent brain and central nervous system cancers (CABCs) represent the leading cause of cancer-related mortality among individuals aged 0–19 years; however, global trends and socio-demographic disparities remain insufficiently explored.

**Methods:**

We utilized the Global Burden of Disease (GBD) 2021 dataset to evaluate the evolving burden of CABCs across 204 countries from 1990 to 2021. We analyzed age-standardized prevalence rate (ASPR), age-standardized incidence rate (ASIR), age-standardized mortality rate (ASMR), and age-standardized disability-adjusted life years (ASDR) among individuals aged 0–19 years. Methodological approaches, including Joinpoint regression, decomposition analysis, and age-period-cohort modeling, were applied to assess trends across five Sociodemographic Index (SDI) levels. Data robustness was further enhanced through temporal smoothing and adjustments to the mortality-to-incidence ratio for pediatric populations.

**Results:**

Globally, ASPR exhibited a modest increase (average annual percentage change [AAPC] = 0.42, 95% CI: 0.29–0.54), while ASIR, ASMR, and ASDR demonstrated significant declines (AAPC = −0.29, −1.31, and −1.34, respectively). High-SDI regions experienced the highest ASPR (18.0 per 100,000) and ASIR (2.6 per 100,000), a reflection of advanced diagnostic capabilities and improved survival outcomes. In contrast, Low-SDI regions saw an upward trajectory in both mortality (AAPC = 0.06) and disability-adjusted life years (DALYs) with the burden disproportionately affecting children under 5 years of age. East Asia accounted for the highest burden of cases (63,271 prevalent cases in 2021), while Sub-Saharan Africa exhibited alarming increases in the incidence among young children. Decomposition analysis revealed that the global reduction in DALYs (−498,553) was predominantly offset by population growth and rising prevalence in low-resource settings.

**Conclusion:**

While advancements in medical care have contributed to the reduction of CABC mortality in high-SDI regions, persistent disparities in Low-SDI areas necessitate urgent interventions. Targeted strategies—such as scalable diagnostic tools, establishment of regional treatment hubs, and equitable financial support through global initiatives like the WHO Childhood Cancer Initiative—are crucial to addressing these disparities. This study underscored the dual challenge of enhancing survivorship outcomes in high-income settings while rectifying systemic healthcare gaps in low-resource regions to promote global equity in pediatric oncology care.

## Introduction

Childhood and adolescent brain and central nervous system cancers (CABCs) are among the most devastating pediatric malignancies, representing a leading cause of cancer-related mortality in individuals aged 0–19 years (1, 2). These tumors pose unique clinical challenges due to their complex neuroanatomical locations and aggressive biology, and survivors often face lifelong neurological, cognitive, and psychosocial sequelae (3, 4). Despite advancements in neuroimaging, surgical techniques, and multimodal therapies, significant disparities persist, with survival outcomes varying widely across socio-economic settings (5, 6). While high-income regions benefit from early detection and state-of-the-art treatment protocols, low- and middle-Sociodemographic Index (SDI) areas often experience diagnostic delays, limited treatment accessibility, and poorer prognoses (7, 8).

Existing epidemiological research on childhood cancers has predominantly focused on high-resource environments, leaving critical gaps in our understanding of CABCs in low-SDI regions (9–11). Additionally, variations in cancer registry completeness and diagnostic practices complicate direct comparisons across countries, potentially masking the true disease burden (12). These limitations underscore the urgent need for comprehensive, standardized global data to inform equitable healthcare policies and optimize resource allocation (13, 14). Recognizing this gap, international initiatives—such as the WHO Global Initiative for Childhood Cancer—have prioritized improving survival rates through early diagnosis and access to effective therapies.

Leveraging the Global Burden of Disease (GBD) 2021 dataset, this study systematically evaluated temporal trends and socio-demographic disparities in the burden of CABCs across 204 countries from 1990 to 2021. Using advanced analytical techniques—including joinpoint regression, decomposition analysis, and age-period-cohort modeling—we assessed changes in age-standardized prevalence rate (ASPR), age-standardized incidence rate (ASIR), age-standardized mortality rate (ASMR), and age-standardized disability-adjusted life years rate (ASDR). In addition, we conduct a frontier analysis to explore the theoretical minimum burden achievable given a country’s SDI level (15, 16), providing a more accurate understanding of global inequities. By identifying key determinants of regional disparities, this study aimed to guide targeted interventions, including scalable diagnostic strategies, regional treatment hubs, and improved access to care, thereby contributing to the broader goal of reducing childhood cancer mortality and advance health equity in pediatric oncology (17).

## Methods

### Study design and data sources

We conducted a repeated cross-sectional study of CABCs using annual estimates from the GBD 2021 study. Specifically, we analyzed GBD data for each calendar year (1990–2021) at the global and regional levels to assess temporal trends in prevalence, incidence, mortality, and disability-adjusted life years (DALYs). Each year’s data constitute an independent cross-sectional “snapshot,” allowing us to examine changes in the burden of CABCs over time.

DALY is a composite metric used to quantify the overall burden of disease (15). It is calculated as the sum of years of life lost (YLLs) due to premature mortality and years lived with disability (YLDs), providing a comprehensive measure of health loss. This metric enables cross-country and cross-disease comparisons by integrating both mortality and morbidity data.

The GBD 2021 dataset provides comprehensive estimates for 371 diseases and injuries across 204 countries and territories by integrating data from cancer registries, vital registration systems, hospital records, and population-based surveys (15). This robust dataset enabled the quantification of incidence, prevalence, mortality, and DALYs associated with CABCs on a global scale. To ensure data quality, we prioritized high-quality cancer registry data where available, supplemented by modeled estimates for regions with incomplete or missing data. Data integration involved rigorous quality control measures, including cross-validation between sources, imputation of missing values using GBD standard methods, and temporal smoothing to reduce fluctuations in regions with sparse data.

Additionally, countries and regions were categorized by SDI, a composite measure based on income per capita, educational attainment, and fertility rates. SDI ranges from 0 to 1, with higher values indicating greater socio-demographic development (18). For analysis purposes, SDI levels were classified into five categories: High, High-middle, Middle, Low- middle, and Low based on GBD-defined thresholds. This stratification facilitates the comparison of disease burden across different stages of development.

As this study utilized anonymized, publicly available data, ethical approval was not required in accordance with institutional guidelines.

### Study population and case definition

The study focused on individuals aged 0–19 years. CABCs were identified in accordance with GBD criteria using International Classification of Diseases, 10th Revision (ICD-10) codes C70–C72 and C75.1–C75.3 (1). These codes were mapped to the GBD classification system through a standardized crosswalk methodology, aligning ICD-10 codes with GBD cause lists to ensure consistency in disease categorization across datasets. To enhance pediatric-specific accuracy, mortality-to-incidence ratios were refined using high-quality cancer registry data, and temporal smoothing techniques were applied to mitigate fluctuations in regions with sparse case data.

### Statistical analysis

#### Age-standardized rates (ASRs)

ASPR, ASIR, ASMR, and ASDR were calculated per 100,000 population by adjusting for variations in age distribution using the GBD reference population. Rates were weighted by age-specific population proportions, and 95% uncertainty intervals (UI) were derived using Monte Carlo simulation methods to reflect estimation precision.

#### Temporal trend analysis

Temporal trends from 1990 to 2021 were examined using joinpoint regression analysis. This method identifies statistically significant changes in trend slopes by allowing for up to five joinpoints, selected based on permutation tests. Statistical significance was assessed using Wald χ^2^ tests, and average annual percentage changes (AAPCs) with corresponding 95% confidence intervals (CIs) were computed to quantify trends over time (1, 19). Additionally, variations in the SDI and regional differences were accounted for to ensure a more comprehensive analysis.

#### Decomposition analysis

The Das Gupta method was used to decompose changes in DALYs into contributions from four distinct factors: population growth, shifts in age structure, changes in disease prevalence, and variations in case fatality/severity. The Das Gupta approach involves calculating the attributable change in DALYs for each component while holding other components constant. Special attention was paid to the pediatric population (0–19 years) to capture the impact of demographic shifts.

#### Age–period–cohort modeling

To disentangle the intertwined effects of age, period, and birth cohort, intrinsic estimator models were utilized. These models allowed estimation of net drift (the overall temporal trend) and local drift (age-specific trends). Cohort effects were normalized to the 1995–1999 birth cohort, and statistical significance was assessed using Wald *χ*^2^ tests (20, 21). Additionally, the models adjusted the SDI and regional differences.

#### Health inequity and frontier analyses

Health inequities were quantified using two complementary metrics. The Slope Index of Inequality (SII) was calculated using regression-based methods to measure absolute disparities, while the Concentration Index (CI) was employed to assess relative disparities, with negative CI values indicating a disproportionate burden in low-SDI regions. Both metrics were computed with 1,000 bootstrap iterations to derive 95% confidence intervals. Additionally, frontier analysis was performed using locally weighted regression (LOESS) with 1,000 bootstrap iterations to model the theoretical minimum achievable ASMR and ASDR at each SDI level. The frontier values were defined as the lowest observed burden for a given SDI level, representing the theoretical minimum achievable under optimal conditions. The difference between observed and frontier values helped identify countries where further improvements in care delivery could be prioritized (22, 23).

#### Software and sensitivity analyses

All analyses were conducted in R (version 4.4.1) and the Joinpoint Regression Program (version 5.2.0). Sensitivity analyses were undertaken to assess the robustness of the findings to potential biases due to missing data or variable data quality across regions. All analytic code and aggregated data are publicly accessible via the Global Health Data Exchange (GHDx).

## Results

### Global burden of CABCs

**Figure 1:**
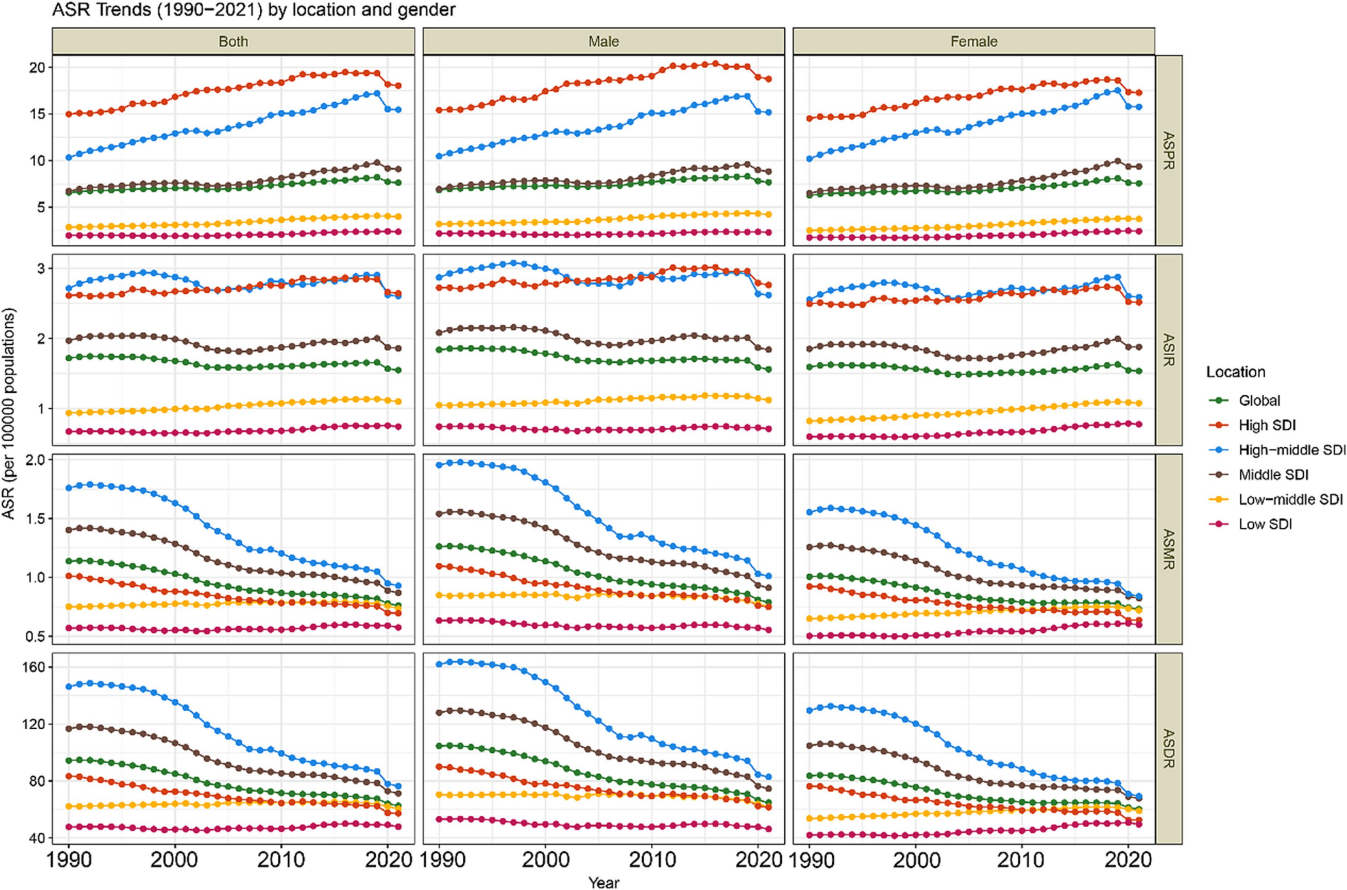
Trends in age-standardized rates (ASRs) of CABCs (1990–2021) by location and gender. SDI, socio-demographic index. CABCs, childhood and adolescent brain and central nervous system cancers. ASPR, age-standardized prevalence rate; ASIR, age-standardized rates for incidence; ASMR, age-standardized mortality rate; ASDR, age-standardized DALY rate; DALYs, disability adjusted life years.

From 1990 to 2021, the global ASPR and ASIR of CABCs remained relatively stable. In contrast, both ASMR and ASDR showed a consistent annual decline across both sexes (Figure 1). In 2021, the global prevalence of CABCs was estimated at 199,214 cases (95% UI: 166,466.2 to 241,711.8), with 40,535 new incidences (95% UI: 33,345.9 to 49,609), 19,923 deaths (95% UI: 16,061.8 to 24,531.8), and 1,632,940 DALYs (95% UI: 1,312,423.7 to 2,014,237). The corresponding ASRs were 7.6 (95% UI: 6.3 to 9.2) for prevalence, 1.5 (95% UI: 1.3 to 1.9) for incidence, 0.8 (95% UI: 0.6 to 0.9) for mortality, and 62 (95% UI: 49.8 to 76.4) for DALYs.

Compared to 1990, the global burden of CABCs showed an increase in prevalence, with an AAPC of 0.42 (95% CI: 0.29 to 0.54). Conversely, incidence, mortality, and DALYs all decreased, with AAPCs of −0.29 (95% CI: −0.41 to −0.17), −1.31 (95% CI: −1.48 to −1.13), and −1.34 (95% CI: −1.51 to −1.16), respectively (Table 1 and Supplementary Table 1).

**Table 1:** Prevalence of CABCs between 1990 and 2021 at the global and regional levels.

Location	Cases number in 1990	ASPR in 1990 (per 100,000 populations)	Cases number in 2021	ASPR in 2021 (per 100,000 populations)	AAPC between 1990–2021 (95% CI)
Global	148027.9 (117699.1 to 178652.0)	6.6 (5.2 to 7.9)	199213.6 (163323.9 to 242620.3)	7.6 (6.2 to 9.3)	0.42 (0.29 to 0.54)
High SDI	37037.1 (34950.7 to 39265.5)	15.0 (14.1 to 15.9)	41858.7 (38307.2 to 45530.1)	18.0 (16.5 to 19.6)	0.59 (0.36 to 0.82)
High-middle SDI	37142.9 (29376.6 to 44808.1)	10.3 (8.1 to 12.5)	46309.3 (36363.4 to 60086.0)	15.5 (12.1 to 20.1)	1.2 (0.86 to 1.55)
Middle SDI	50505.2 (35446.8 to 62626.0)	6.7 (4.7 to 8.3)	67076.6 (50590.4 to 85557.0)	9.1 (6.8 to 11.6)	0.88 (0.67 to 1.09)
Low-middle SDI	17306.9 (11930.0 to 26247.6)	2.9 (2.0 to 4.3)	29904.1 (22632.7 to 38212.5)	4.0 (3.0 to 5.1)	1.08 (1.01 to 1.14)
Low SDI	5920.2 (3497.3 to 11214.9)	2.0 (1.2 to 3.7)	13938.1 (9333.4 to 18648.7)	2.3 (1.6 to 3.1)	0.62 (0.38 to 0.86)
Andean Latin America	966.9 (656.9 to 1558.2)	5.1 (3.4 to 8.1)	2011.7 (1393.2 to 2941.2)	8.5 (5.9 to 12.5)	1.69 (1.31 to 2.07)
Australasia	764.8 (613.2 to 957.0)	12.4 (10.0 to 15.5)	920.7 (656.2 to 1278.0)	12.2 (8.7 to 17.0)	0.03 (−0.37 to 0.44)
Caribbean	786.4 (592.3 to 1259.0)	5.2 (3.9 to 8.4)	897.6 (670.4 to 1264.8)	5.9 (4.4 to 8.4)	0.43 (0.09 to 0.76)
Central Asia	1792.8 (1396.6 to 2311.6)	5.6 (4.4 to 7.1)	2964.8 (2386.2 to 3721.7)	8.4 (6.8 to 10.6)	1.41 (0.98 to 1.84)
Central Europe	3473.9 (3112.8 to 3916.2)	9.1 (8.1 to 10.2)	2194.3 (1837.7 to 2587.2)	9.3 (7.8 to 11.0)	0.13 (−0.2 to 0.46)
Central Latin America	3360.6 (3079.6 to 3710.0)	4.1 (3.7 to 4.5)	4247.6 (3515.6 to 5179.9)	5.0 (4.1 to 6.1)	0.63 (0.36 to 0.91)
Central Sub-Saharan Africa	287.7 (145.7 to 614.8)	0.9 (0.5 to 1.8)	695.0 (433.0 to 1061.4)	0.9 (0.6 to 1.4)	0.21 (0.1 to 0.33)
East Asia	50976.4 (34178.4 to 65573.0)	11.6 (7.8 to 14.9)	63270.9 (46660.1 to 87062.1)	18.5 (13.6 to 25.6)	1.4 (1.14 to 1.65)
Eastern Europe	4403.1 (4017.8 to 4867.8)	6.6 (6.0 to 7.3)	3284.6 (2936.6 to 3664.0)	7.3 (6.5 to 8.2)	0.3 (−0.2 to 0.8)
Eastern Sub-Saharan Africa	2874.8 (1856.6 to 4898.8)	2.4 (1.5 to 4.0)	6670.9 (4536.8 to 9614.1)	2.9 (2.0 to 4.2)	0.69 (0.49 to 0.89)
High-income Asia Pacific	6415.8 (5294.0 to 7691.1)	13.3 (11.0 to 16.0)	7003.2 (5273.5 to 9007.2)	23.0 (17.3 to 29.7)	1.73 (0.81 to 2.66)
High-income North America	16273.1 (15083.2 to 17599.2)	20.1 (18.6 to 21.7)	18087.3 (16261.5 to 20111.4)	20.3 (18.2 to 22.6)	0.06 (−0.33 to 0.45)
North Africa and Middle East	13468.0 (8666.9 to 20507.9)	7.5 (4.8 to 11.3)	30493.1 (20473.2 to 40687.1)	13.0 (8.7 to 17.3)	1.81 (1.63 to 2)
Oceania	34.1 (16.9 to 55.5)	1.0 (0.5 to 1.6)	74.9 (40.4 to 119.0)	1.2 (0.6 to 1.8)	0.46 (0.09 to 0.84)
South Asia	13893.9 (8291.0 to 21566.3)	2.5 (1.5 to 3.9)	20125.8 (14985.7 to 27010.8)	3.0 (2.2 to 4.1)	0.6 (0.3 to 0.9)
Southeast Asia	6074.9 (3683.2 to 8916.9)	2.8 (1.7 to 4.1)	8335.0 (5799.1 to 10629.2)	3.6 (2.5 to 4.7)	0.89 (0.8 to 0.98)
Southern Latin America	1039.8 (773.0 to 1391.1)	5.4 (4.0 to 7.2)	1720.2 (1235.9 to 2426.1)	8.9 (6.4 to 12.6)	1.57 (0.87 to 2.26)
Southern Sub-Saharan Africa	426.7 (312.6 to 604.3)	1.6 (1.2 to 2.3)	730.0 (524.0 to 969.0)	2.3 (1.7 to 3.1)	1.17 (0.52 to 1.83)
Tropical Latin America	4098.2 (3472.4 to 4738.4)	6.0 (5.1 to 7.0)	4661.0 (3823.9 to 5517.5)	7.0 (5.8 to 8.4)	0.45 (0.07 to 0.83)
Western Europe	15299.4 (13987.8 to 16887.3)	15.9 (14.5 to 17.5)	16866.2 (14914.3 to 19021.3)	18.4 (16.3 to 20.8)	0.42 (0.16 to 0.68)
Western Sub-Saharan Africa	1316.6 (852.6 to 1948.5)	1.1 (0.7 to 1.6)	3958.6 (1913.8 to 5415.8)	1.4 (0.7 to 1.9)	0.79 (0.68 to 0.9)

**Figure 2:**
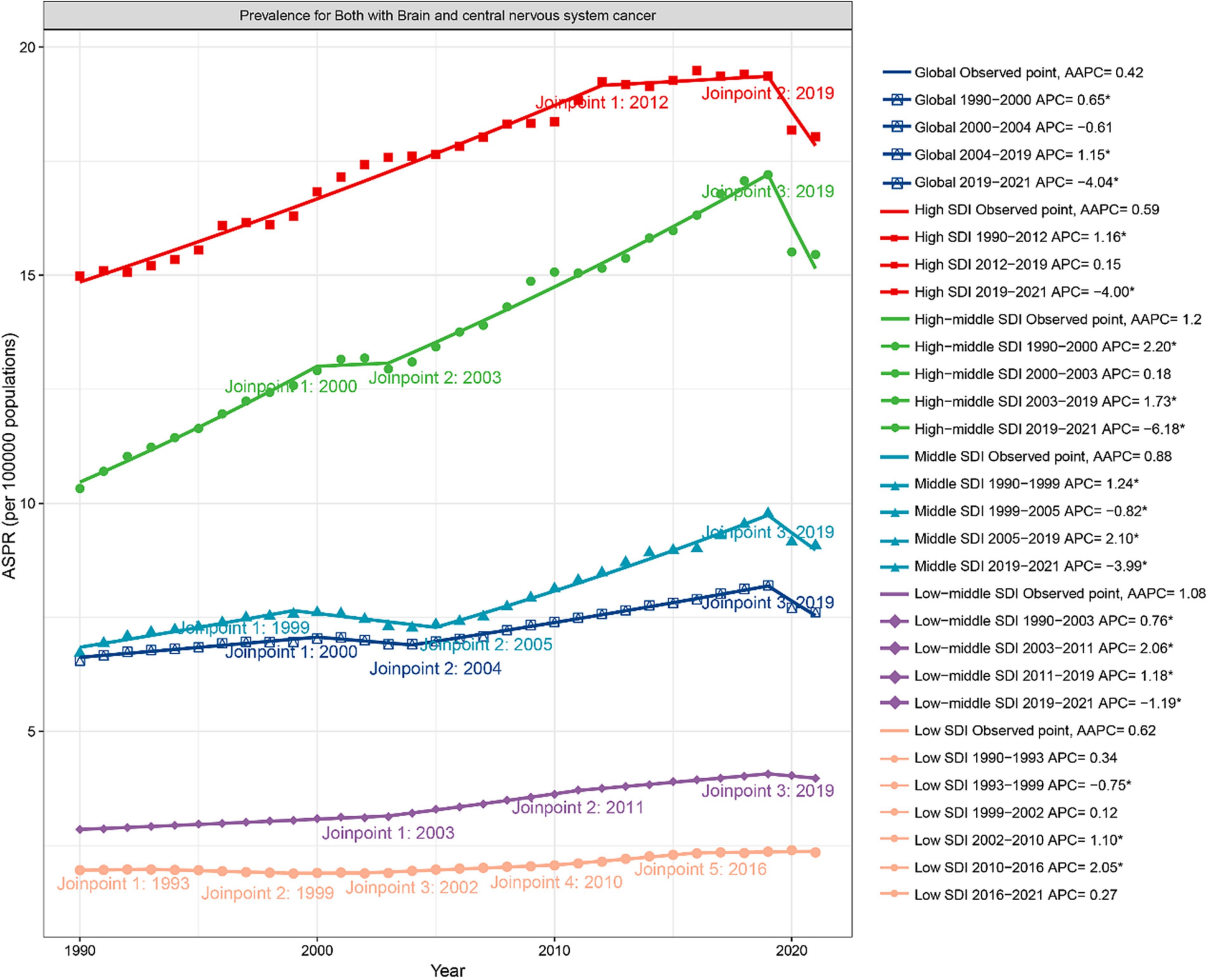
Joinpoint regression analysis of prevalence of CABCs at global and SDI levels (1990–2021). SDI, socio-demographic index; CABCs, childhood and adolescent brain and central nervous system cancers; ASPR, age-standardized prevalence rate; AAPC, average annual percentage changes. APC, annual percentage change.

Joinpoint regression analysis revealed that the global ASPR increased until 2019, after which it declined significantly (APC = −4.04) (Figure 2). Meanwhile, ASIR slightly increased from 2004 to 2019 (APC = 0.31) (Supplementary Figure 1). Over the past three decades, both ASMR and ASDR consistently decreased (Supplementary Figures 2, 3).

### SDI burden of CABCs

The burden of CABCs varied significantly by SDI level. High SDI regions consistently exhibited the highest ASPR, followed by High-Middle SDI regions, which also maintained higher ASIRs. For ASMR and ASDR, High-Middle SDI regions showed elevated levels, while Low SDI regions had the lowest rates across all metrics (Figure 1). In 2021, High SDI regions had the highest ASPR at 18.0 (95% UI: 16.5 to 19.6), while High-Middle SDI regions reported the highest ASIR, ASMR, and ASDR at 2.6 (95% UI: 2.1 to 3.4), 0.9 (95% UI: 0.8 to 1.2), and 76.3 (95% UI: 62.3 to 96.0), respectively (Table 1 and Supplementary Table 1).

From 1990 to 2021, prevalence increased across all SDI regions, with High-Middle SDI regions experiencing the highest growth rate (AAPC = 1.2, 95% CI: 0.86 to 1.55). Notably, Low SDI regions experienced a gradual but consistent increase in prevalence, suggesting a growing burden. While incidence decreased in High-Middle and Middle SDI regions, other SDI regions, particularly Low SDI regions, saw slight increases in incidence. Mortality and DALYs increased minimally in Low SDI regions (AAPC = 0.06, 95% CI: −0.15 to 0.26 and AAPC = 0.04, 95% CI: −0.16 to 0.24, respectively), but decreased in other SDI regions (Table 1 and Supplementary Table 1).

Post-2019 trends highlighted regional disparities, with Low SDI regions showing a worrying trend of stagnation and gradual increase in several metrics. While ASPR declined in all SDI regions except Low SDI, where it remained stable, the burden in Low SDI regions did not decrease as significantly as in other regions. High-Middle SDI regions experienced the most significant decline in ASPR (APC = −6.18) (Figure 2). Conversely, ASIR in Low SDI regions showed a steady increase, reflecting a lack of significant progress in reducing disease burden (Supplementary Figure 1). Low-Middle and Low SDI regions showed flatter trends for ASIR, ASMR, and ASDR, but these rates began declining only after 2016, indicating a delayed response to interventions (Supplementary Figures 2, 3).

The Middle SDI region had the highest burden, particularly among children aged 0–14 years. Increases in prevalence were most notable in 10–14-year-olds, while mortality and DALYs were concentrated in younger children. However, Low SDI regions showed a concerning annual increase in all metrics, particularly in children under 5, underscoring the increasing burden in these areas (Supplementary Figure 4).

### Regional burden of CABCs

The burden of CABCs displayed significant regional variation. East Asia reported the highest overall burden across all age groups, followed by South Asia and North Africa and the Middle East. In contrast, Sub-Saharan Africa, particularly in the Western and Eastern regions, saw annual increases in burden, especially among children under 5 years old. Oceania, on the other hand, exhibited the lowest burden across all age groups (Supplementary Figure 5).

In 2021, East Asia had the highest prevalence (63,270.9; 95% UI: 46,660.1 to 87,062.1) and incidence (10,667.4; 95% UI: 7,894.9 to 14,713.6), with an ASIR of 3.1 (95% UI: 2.3 to 4.3). South Asia recorded the highest number of deaths and DALYs, while Central Asia exhibited the highest ASMR and ASDR (Table 1 and Supplementary Table 1).

When comparing the situation to 1990, prevalence increased across all regions. Notably, North Africa and the Middle East experienced the highest growth rate (AAPC = 1.81, 95% CI: 1.63 to 2.00). High-Income Asia Pacific had the highest incidence growth rate (AAPC = 1.18, 95% CI: 0.36 to 2.00), whereas Central Europe showed the smallest decrease in incidence (AAPC = −0.92, 95% CI: −1.25 to −0.59). Southern Sub-Saharan Africa experienced the largest increases in mortality and DALYs (AAPC = 1.04 and 1.03, respectively), while East Asia demonstrated the most substantial reductions in these metrics (AAPC = −2.55 and −2.60, respectively) (Table 1 and Supplementary Table 1).

A strong positive correlation was observed between ASPR and ASIR with the SDI (*ρ* = 0.79 and ρ = 0.73, respectively), suggesting that regions with higher SDI levels tended to have higher disease prevalence and incidence. Conversely, ASMR and ASDR showed a non-linear relationship with SDI, peaking in regions with moderate SDI levels (e.g., East Asia and Central Asia) before declining in regions with higher SDI (Supplementary Figure 6).

### National burden of CABCs

**Figure 3:**
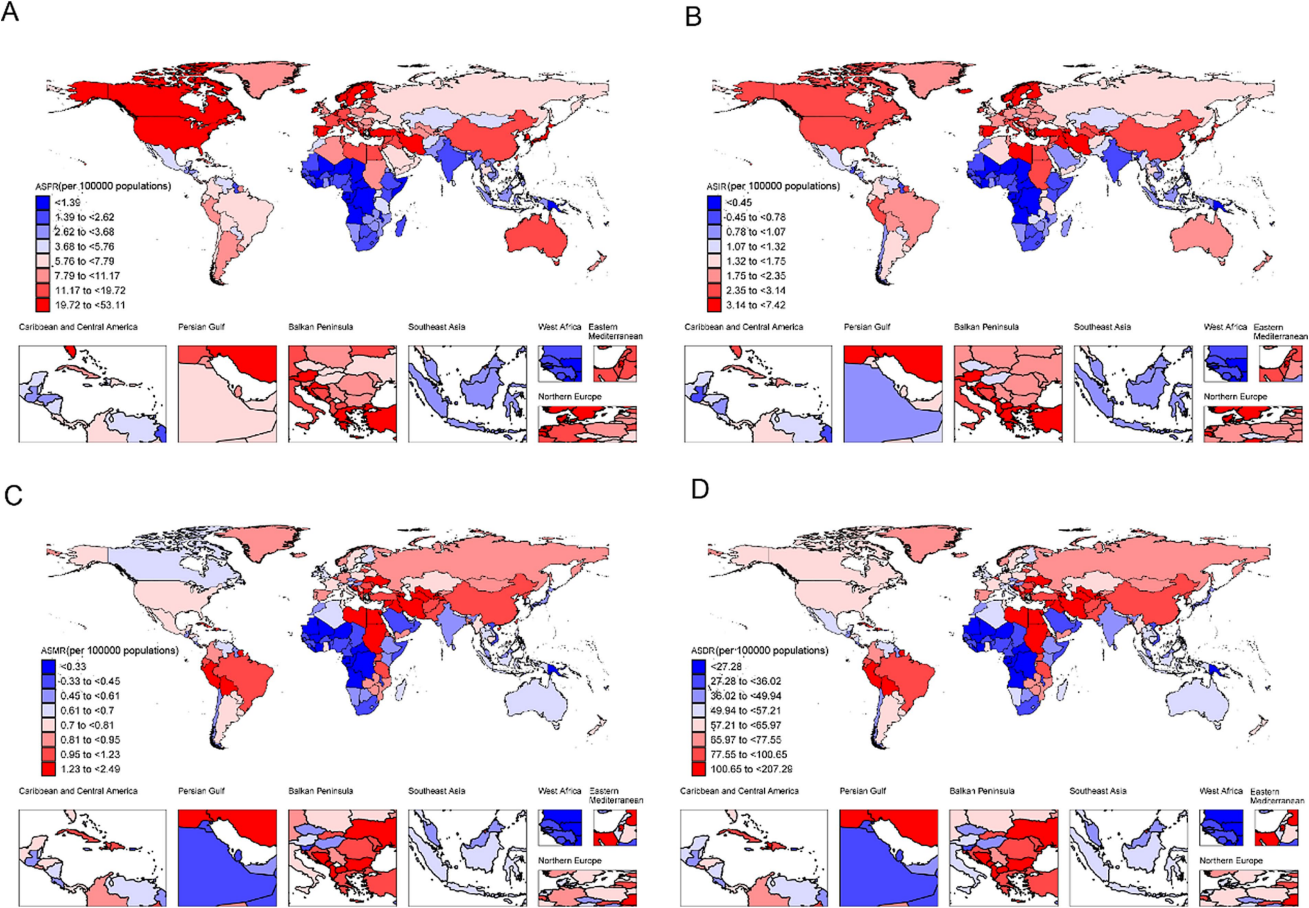
The burden of CABCs at national level in 2021. **(A)** ASPR, **(B)** ASIR, **(C)** ASMR, **(D)** ASDR. Countries are color-coded based on the octile (8 quantiles) of each metric, with the color gradient ranging from dark blue (lowest burden) to dark red (highest burden). The color assigned to each country represents its burden level for the specific metric in 2021. CABCs, childhood and adolescent brain and central nervous system cancers; ASPR, age-standardized prevalence rate; ASIR, age-standardized incidence rate; ASMR, age-standardized mortality rate; ASDR, age-standardized DALY rate; DALYs, disability adjusted life years.

On a national level, China and the United States had the highest prevalence in 2021, while China and India reported the highest incidence, deaths, and DALYs. Smaller nations, such as the Cook Islands and Niue, had the lowest numbers across all metrics due to their smaller populations (Supplementary Table 2). Monaco exhibited the highest ASPR and ASIR, at 54.4 (95% UI: 29.3 to 89.9) and 7.6 (95% UI: 4.1 to 12.5), respectively (Figures 3A,B and Supplementary Table 2). A total of 82 and 90 countries exceeded global ASPR and ASIR levels, respectively. Additionally, Tajikistan had the highest ASMR at 2.4 (95% UI: 1.5 to 3.9) (Figure 3C and Supplementary Table 2), and Tokelau reported the highest ASDR at 203.3 (95% UI: 101.6 to 315.3) (Figure 3D and Supplementary Table 2). The Gambia recorded the lowest ASPR and ASIR, while the Cook Islands had the lowest ASMR and ASDR (Figure 3 and Supplementary Table S2).

From 1990 to 2021, countries like Tokelau experienced significant increases in prevalence, incidence, mortality, and DALYs, with AAPCs of 9.89 (95% CI: 9.42 to 10.35), 9.11 (95% CI: 8.45 to 9.77), 8.32 (95% CI: 7.52 to 9.13), and 8.44 (95% CI: 7.60 to 9.29), respectively. Greenland showed the greatest reductions in prevalence and incidence (AAPC = −1.47, 95% CI: −1.81 to −1.13; APC = −2.09, 95% CI: −2.42 to −1.75), while Serbia exhibited the largest decreases in mortality and DALYs (AAPC = −2.85, 95% CI: −3.31 to −2.38; APC = −2.89, 95% CI: −3.37 to −2.41) (Supplementary Table 2). ASPR and ASIR were positively correlated with SDI (*ρ* = 0.75 and *ρ* = 0.66, respectively), while ASMR and ASDR reached peak levels in mid-SDI regions before declining in higher-SDI areas (Supplementary Figures 7–10).

### Decomposition analysis of DALYs in CABCs

**Figure 4:**
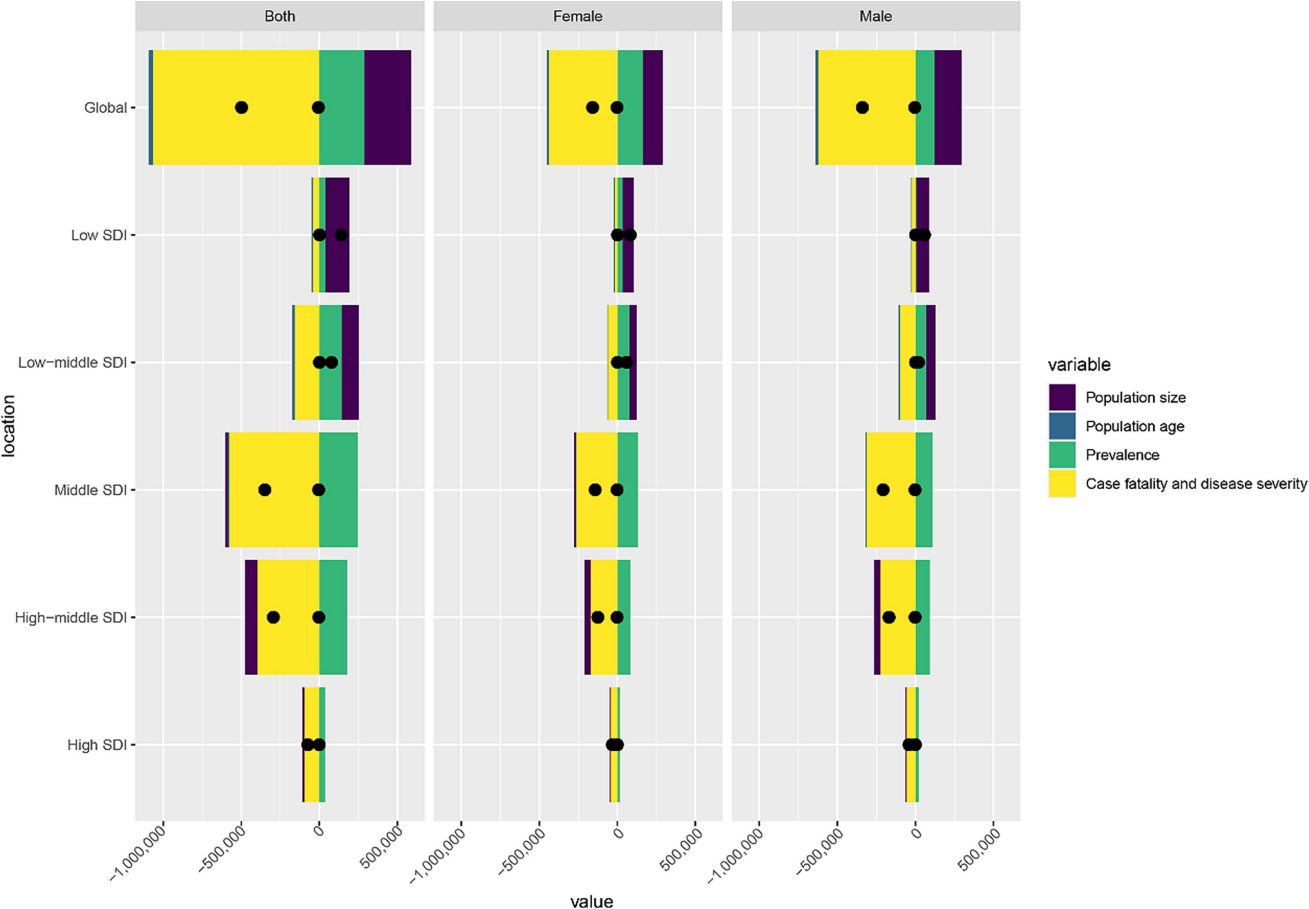
Decomposition analysis of DALYs burden for CABCs in 2021 by location and gender. SDI, socio-demographic index; CABCs, childhood and adolescent brain and central nervous system cancers; DALYs, disability adjusted life years.

Globally, DALYs decreased by 498,553, largely due to a reduction in case fatality and disease severity (contribution: −1,052,909; −211%). However, this was offset by an increase in prevalence (contribution: 283,801; 57%) and a declining population size (contribution: −59.8%). In regions with Low-SDI and Low-Middle SDI, DALYs rose by 140,352 and 78,442, respectively, driven mainly by population growth and increased prevalence. Although reductions in case fatality and disease severity mitigated some of these increases, they were insufficient to counterbalance these upward trends. In contrast, Middle-SDI, High-SDI, and High-Middle SDI regions experienced significant reductions in DALYs of −349,205, −73,230, and −294,463, respectively, largely due to improvements in case fatality and disease severity, despite rising prevalence (Figure 4).

Trends in DALYs reductions were consistent across males and females, with slightly greater reductions in males, possibly reflecting disparities in disease management or resource allocation (Figure 4). These findings underscore global progress in reducing CABCs-related mortality and severity, especially in higher-SDI regions. However, the rising prevalence and population growth in Low-SDI regions highlight the continuing challenges and the need for targeted interventions in resource-limited settings.

### Age-period-cohort analysis

**Figure 5:**
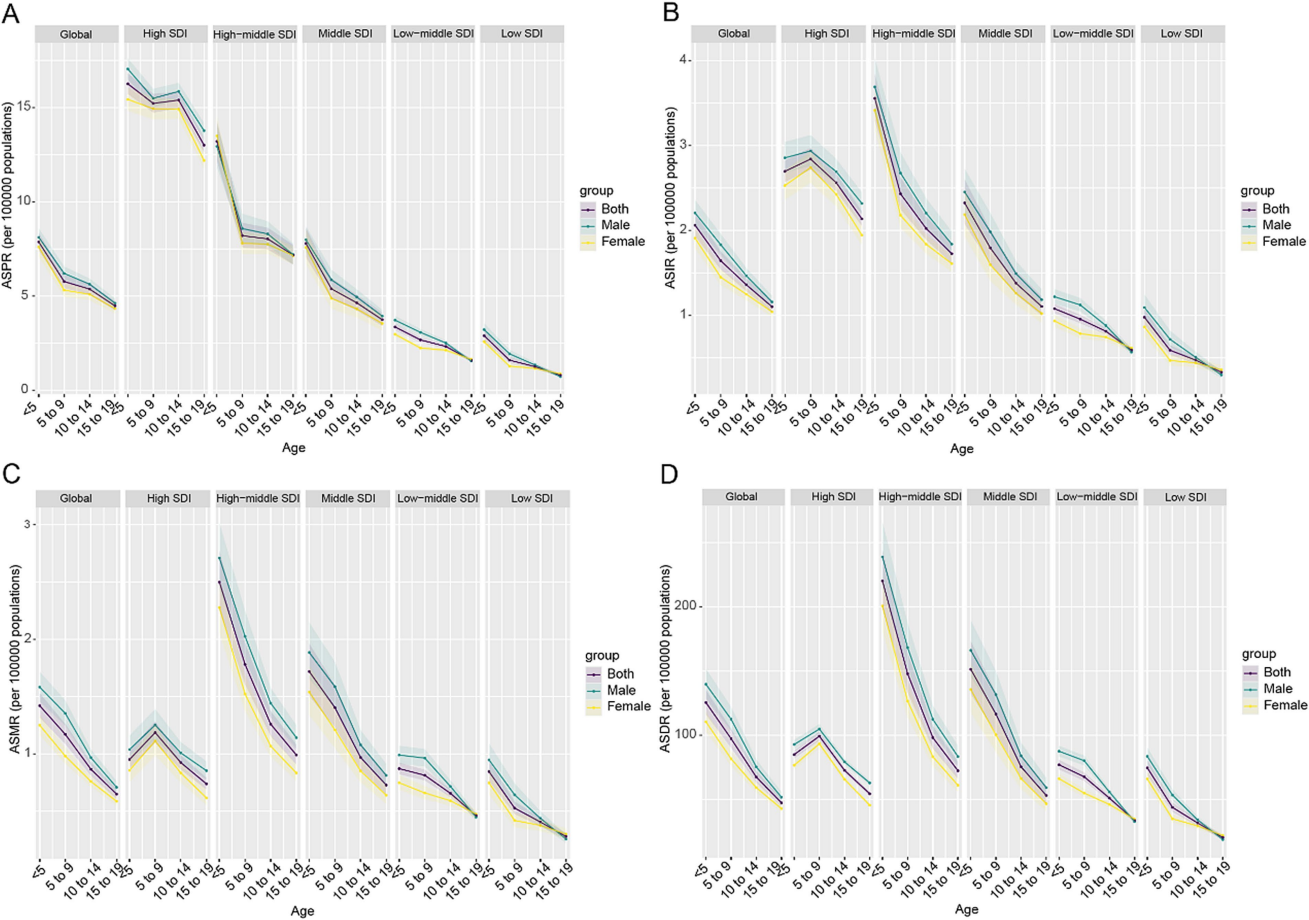
Age-specific trends in ASR metrics of CABCs by location and gender in 2021. **(A)** ASPR, **(B)** ASIR, **(C)** ASMR, **(D)** ASDR. SDI, socio-demographic index; CABCs, childhood and adolescent brain and central nervous system cancers; ASPR, age-standardized prevalence rate; ASIR, age-standardized incidence rate; ASMR, age-standardized mortality rate; ASDR, age-standardized DALY rate; DALYs, disability adjusted life years.

The Age-Period-Cohort analysis revealed distinct patterns in CABCs burden. Globally, ASPR, ASIR, ASMR, and ASDR peaked in children under 5 years of age, followed by a decline with age. This decline was more pronounced in High-SDI regions, while Low-SDI regions sustained higher overall burden levels. Notably, children aged 5–9 years in High-SDI regions exhibited higher ASIR, ASMR, and ASDR compared to other age groups (Figure 5).

Sex differences were evident, with males generally exhibiting higher incidence and mortality rates than females (Figures 5B,C). However, in Low-SDI and Low-Middle SDI regions, females demonstrated a more pronounced increase in the risk ratio (RR) of prevalence and incidence over time, likely reflecting disparities in healthcare access and awareness. Similarly, RRs for mortality and DALYs declined globally, with significant reductions in High-SDI regions. However, a resurgence was observed among recent female cohorts in Low-SDI regions, indicating worsening disease burdens in these resource-limited settings (Supplementary Figure 11). Annual percentage changes revealed increasing prevalence and incidence with age across all SDI levels, especially among female adolescents in Low-SDI regions. Mortality and DALYs showed significant annual decreases globally, especially in High-SDI regions, emphasizing the positive impact of medical advancements. However, Low-SDI regions showed an increasing trend in mortality and DALYs among females aged 15–19 years, signaling the need for targeted interventions in this demographic (Supplementary Figure 12). Cohort effects further revealed that recent birth cohorts (2000s) experienced higher prevalence and incidence risks, particularly in Low and Low-Middle SDI regions, compared to earlier cohorts (1970s–1980s). This trend was especially notable for females, reflecting potential disparities in disease management or delayed interventions (Supplementary Figure 13).

### Frontier analysis and health inequity analysis

Frontier analysis between 1990 and 2021 indicated general declines in ASMR and ASDR for CABCs across most countries. However, despite overall improvements, nations such as Monaco, Tokelau, Azerbaijan, Albania, Turkmenistan, and Uzbekistan continued to experience a substantial burden of mortality and DALYs, even in higher SDI regions (Supplementary Figure 14).

**Figure 6:**
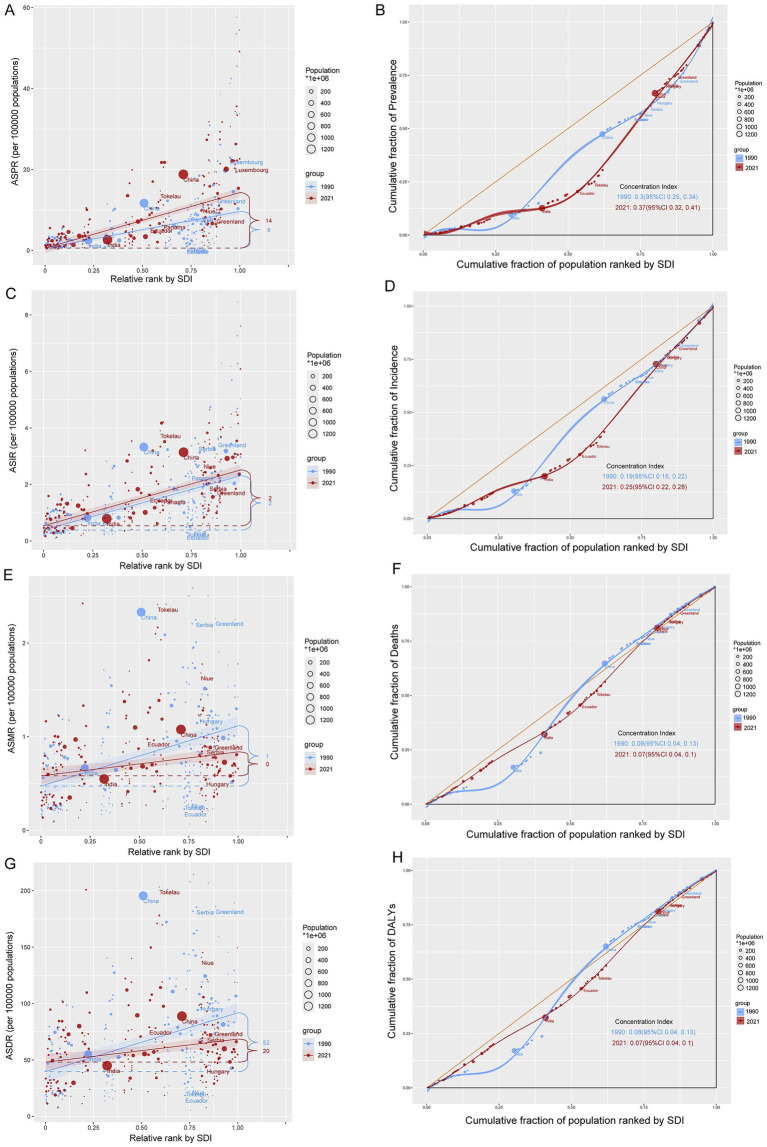
Absolute and relative cross-country inequality of CABCs, 1990–2021. Slope index of **(A)** ASPR, **(C)** ASIR, **(E)** ASMR and **(G)** ASDR. Countries are ranked by SDI on the *x*-axis. The *y*-axis shows the metric value. Each point represents a country, sized by its population (1990: light blue; 2021: red). The solid line (slope) indicates the health inequality trend. The numbers denote the count of countries with high values for each metric in 1990 and 2021. Concentration index of **(B)** prevalence, **(D)** incidence, **(F)** mortality and **(H)** DALYs. The *y*-axis shows the cumulative proportion of the disease metric burden. The yellow diagonal line represents the line of equality (where points would lie if burden was distributed equally across the SDI gradient). CI measures relative inequality across the entire socioeconomic distribution (range: −1 to +1). A positive CI indicates higher burden in higher-SDI countries; an increase in CI from 1990 to 2021 signifies worsening inequality. SDI, socio-demographic index; CABCs, childhood and adolescent brain and central nervous system cancers; ASPR, age-standardized prevalence rate; ASIR, age-standardized incidence rate; ASMR, age-standardized mortality rate; ASDR, age-standardized DALY rate; DALYs, disability adjusted life years.

Health inequity analysis revealed an intensification of CABCs prevalence and incidence over time. From 1990 to 2021, the concentration index for ASPR increased from 0.3 to 0.37, and for ASIR, from 0.19 to 0.25 (Figures 6A–D). These findings suggest that regions with higher SDI levels consistently bore a greater burden of prevalence and incidence. On the other hand, health inequities in ASMR and ASDR showed a decline, reflected by decreasing concentration indices (from 0.09 to 0.07), indicating relative reductions in mortality and DALYs in Low-SDI regions (Figures 6E–H).

## Discussion

This study analyzes the global, regional, and national burden of CABCs from 1990 to 2021. Our findings indicate that while the ASPR and ASIR remained relatively stable globally, there was a consistent annual decline in ASMR and ASDR across both sexes. In 2021, global CABCs prevalence reached 199,214 cases, with substantial variations across SDI regions and geographical areas. High SDI regions exhibited the highest ASPR and ASIR, likely due to advanced diagnostic capabilities and improved case detection, whereas low SDI regions showed alarming increases in prevalence, incidence, mortality, and DALYs despite having the lowest overall burden. These trends underscore both progress in management and widening healthcare disparities.

The stability of ASPR and ASIR globally suggests no significant increase in actual CABCs occurrence over three decades, potentially reflecting advancements in diagnostic technologies enabling earlier detection rather than true rising incidence (24–27). However, underdiagnosis remains a critical concern in low SDI regions, where limited diagnostic tools, incomplete cancer registries, and delayed healthcare-seeking behaviors (28, 29) may lead to case underestimation. Discrepancies between high and low SDI regions arise not only from true disease frequency differences but also variations in diagnostic capacity. High SDI countries benefit from universal health coverage, advanced imaging (MRI, CT), and robust cancer surveillance, while low SDI regions often lack infrastructure, resulting in missed diagnoses and artificially lower reported burdens.

The consistent decline in ASMR and ASDR indicates substantial improvements in treatment outcomes, particularly in high SDI regions. This progress stems from medical advancements (30), early diagnosis (31), and more effective therapies (32), including multimodal approaches with advanced radiotherapy, targeted treatments, and immunotherapies (33–36). However, increasing CABCs survivors highlight the growing need for long-term follow-up addressing neurological, cognitive, and psychosocial challenges through integrated rehabilitation and survivorship care (37–40).

Distinct regional disparities exist. High and High-Middle SDI regions show elevated ASPR and ASIR, likely reflecting enhanced diagnostics through screening and advanced imaging (41–43) rather than genuine incidence increases. Conversely, Low SDI regions face rising prevalence, incidence, mortality, and DALYs, driven by population growth (44), environmental/genetic risk factors (45), malnutrition, poor maternal-child healthcare, and environmental toxins (46, 47). Late-stage diagnoses due to limited healthcare access (48) worsen outcomes in these regions. Mortality and DALY declines in higher SDI regions underscore the importance of early detection programs and advanced treatments (29).

Significant geographic variations were observed, with East Asia bearing the highest burden, followed by South Asia and North Africa/Middle East. These patterns may stem from genetic predispositions, environmental exposures (49), healthcare infrastructure, and resource access (50). East Asian populations may have higher susceptibility to specific CNS tumors potentially due to genetics, while air pollution, diet, and prenatal factors (51) could contribute. Western and Eastern Sub-Saharan Africa experience rising burden among children under five, highlighting urgent needs for pediatric oncology services and diagnostics (44). The lack of specialized facilities and trained oncologists exacerbates disparities, necessitating region-specific interventions like expanding MRI/CT access and professional training (52). Oceania maintains the lowest burden, possibly due to smaller populations and effective healthcare (53).

At the national level, populous countries like China, India, and the United States report the highest cases, deaths, and DALYs, reflecting large populations (54) and higher detection rates (55). Smaller nations like Cook Islands report the lowest burdens. Interestingly, Monaco and Tokelau have exceptionally high ASPR and ASIR, possibly due to advanced diagnostics in Monaco (56) and small population effects in Tokelau (57). These national disparities highlight healthcare infrastructure, public health policies, and socioeconomic conditions (58–60) as critical determinants, requiring tailored interventions.

Decomposition analysis reveals that the global ~50% DALY reduction primarily results from improved case fatality and reduced severity (45), driven by multidisciplinary care teams and oncology centers (61). However, prevalence increases and population growth partially offset gains in low SDI regions. Despite case fatality improvements (62), rising affected child numbers outpace healthcare capacity (63), straining limited resources and demanding urgent investments in early detection and capacity building.

Age-Period-Cohort analysis shows the highest burden among children under five, with more pronounced declines in ASMR/ASDR in High SDI regions (64), highlighting the role of early intervention. In Low and Low-Middle SDI regions, relative risk for prevalence and incidence increased over time, particularly among females (65), suggesting emergent gender-based barriers in patriarchal societies where girls face delayed care. This female-specific increase may partly reflect cultural and structural barriers—such as restrictions on girls’ mobility, lower household decision-making power, and discriminatory attitudes of providers—that delay diagnosis and treatment; intersectionality work in agro-pastoral Tanzania and WHO analyses highlight how these factors disproportionately hinder girls’ access to care (66, 67). Conversely, declining mortality and DALYs in High SDI regions reflect established oncology services and survivorship programs (29, 68).

Frontier analysis identifies inefficiencies relative to expected burdens. Countries like Monaco and Tokelau show gaps between observed ASDR and frontier values (69), indicating potential inefficiencies in resource use or systemic care barriers requiring optimized diagnostics and care coordination (70). Conversely, Tajikistan and Uzbekistan represent priorities for international aid given their disproportionately high disease burdens, necessitating cost-effective diagnostics like portable MRI (71), workforce training, and regional treatment hubs (9). We note that our frontier analysis represents relative efficiency in converting health-system inputs into outcomes, and does not imply an absolute minimum incidence or mortality rate achievable regardless of genetic, environmental, or other non-health-system determinants.

Health inequity analysis confirms widening disparities, with concentrated ASMR/ASDR burdens (negative Concentration Index) in low-SDI regions due to diagnostic/treatment barriers (63). Global initiatives like WHO’s ‘Global Initiative for Childhood Cancer’ must prioritize low-SDI regions through funding, technical support (72), and community health worker integration (73) to facilitate early referrals. While High SDI regions show increased prevalence/incidence from superior diagnostics, low-SDI regions require enhanced cancer registries and provider training to address underreporting (74).

The study highlights critical global health inequities, emphasizing persistently high low-SDI mortality requiring interventions like portable MRI for early detection and regional treatment centers. Strengthening global collaboration through WHO programs (29), developing regional oncology networks for resource sharing, and workforce initiatives (75) are essential. Prevention strategies including nutritional support and school-based screenings (76) can reduce risks. Together, these measures can enhance diagnostics and treatment while reducing inequities in resource-limited settings.

Despite these insights, our study has limitations. The reliance on GBD data and aggregated metrics may introduce measurement bias and limit histology-specific analyses. For example, limited cancer registry data in low-income regions may underestimate the true disease burden, while variations in diagnostic criteria across regions can lead to inconsistencies. Regional differences in data quality, incomplete cancer registries, and diagnostic criteria variations can influence observed patterns. Although we adjusted for cancer registry completeness, estimates in low-SDI countries remain considerably less precise: the 95% UIs are approximately 3 times larger than in high-SDI settings (see Table 1 and Supplementary Tables). Such wider intervals may obscure true geographic differences in burden and should be interpreted with caution. Moreover, because detailed histology-specific data (e.g., on medulloblastoma vs. glioma) were unavailable, our aggregate estimates of childhood and adolescent brain cancer may conceal divergent trends by subtype, which could limit the specificity of policy recommendations. Future research should incorporate more detailed data, investigate genetic and environmental risk factors, and conduct longitudinal cohort studies to clarify causality. Additionally, qualitative research on healthcare access barriers in low-SDI settings can guide could inform context-specific interventions and policy reforms.

## Conclusion

This study highlights significant progress in reducing mortality and DALYs associated with CABC globally, especially in high-SDI regions. However, the increasing prevalence and burden in low- and low-middle SDI regions underscore ongoing and emerging challenges. Addressing these disparities requires targeted public health interventions, equitable resource allocation, and continued advancements in medical care. By understanding the complex dynamics of CABC burden across different socio-demographic contexts, stakeholders can develop informed strategies to mitigate the global impact of these devastating cancers on children and adolescents.

## Data Availability

The original contributions presented in the study are included in the article/Supplementary material, further inquiries can be directed to the corresponding authors.
