# Supplementary material for: Worldwide patterns and trends in childhood and adolescent brain cancers, 1990–2021: insights from the global burden of disease study 2021
Source: Front Public Health. 2025 Sep 12;13:1591309. doi: 10.3389/fpubh.2025.1591309 (PMC12463948; doi:10.3389/fpubh.2025.1591309)
Supplement: Supplementary file 1 [file Data_Sheet_1.docx]

**Contents**

[Supplementary Figure 1. Joinpoint regression analysis of incidence of CABCs at global and SDI levels (1990–2021). 2](#_Toc200551180)

[Supplementary Figure 2. Joinpoint regression analysis of mortality of CABCs at global and SDI levels (1990–2021). 3](#_Toc200551181)

[Supplementary Figure 3. Joinpoint regression analysis of DALYs of CABCs at global and SDI levels (1990–2021). 4](#_Toc200551182)

[Supplementary Figure 4. The number of incidence, prevalence, deaths and DALYs of CABCs at SDI levels in different age groups (1990–2021). 5](#_Toc200551183)

[Supplementary Figure 5. The number of incidence, prevalence, deaths and DALYs of CABCs at regional levels in different age groups (1990–2021). 6](#_Toc200551184)

[Supplementary Figure 6. The relative of ASR metrics and SDI in global and regional levels of CABCs from1990 to 2021. 8](#_Toc200551185)

[Supplementary Figure 7. The relative of ASPR and SDI in national level of CABCs in 2021. 9](#_Toc200551186)

[Supplementary Figure 8. The relative of ASIR and SDI in national level of CABCs in 2021. 10](#_Toc200551187)

[Supplementary Figure 9. The relative of ASMR and SDI in national level of CABCs in 2021. 11](#_Toc200551188)

[Supplementary Figure 11. Periods-specific trends in ASR metrics of CABCs by location and gender. 13](#_Toc200551189)

[Supplementary Figure 12. Age-annual change trends in ASR metrics of CABCs by location and gender in 2021. 14](#_Toc200551190)

[Supplementary Figure 13. The cohort effect in ASR metrics of CABCs by location and gender. 15](#_Toc200551191)

[Supplementary Figure 14. Frontier analysis of ASMR and ASDR of CABCs. (A, C) 16](#_Toc200551192)

[Supplementary Table 1. Incidence, Deaths and DALYs of CABCs between 1990 and 2021 at the Global and Regional Levels 17](#_Toc200551193)

[Supplementary Table 2. Prevalence, Incidence, Deaths and DALYs of CABCs between 1990 and 2021 at the National level 23](#_Toc200551194)

[Supplementary Table 3. Decomposition analysis of DALYs burden for CABCs in 2021 by location and gender 75](#_Toc200551195)


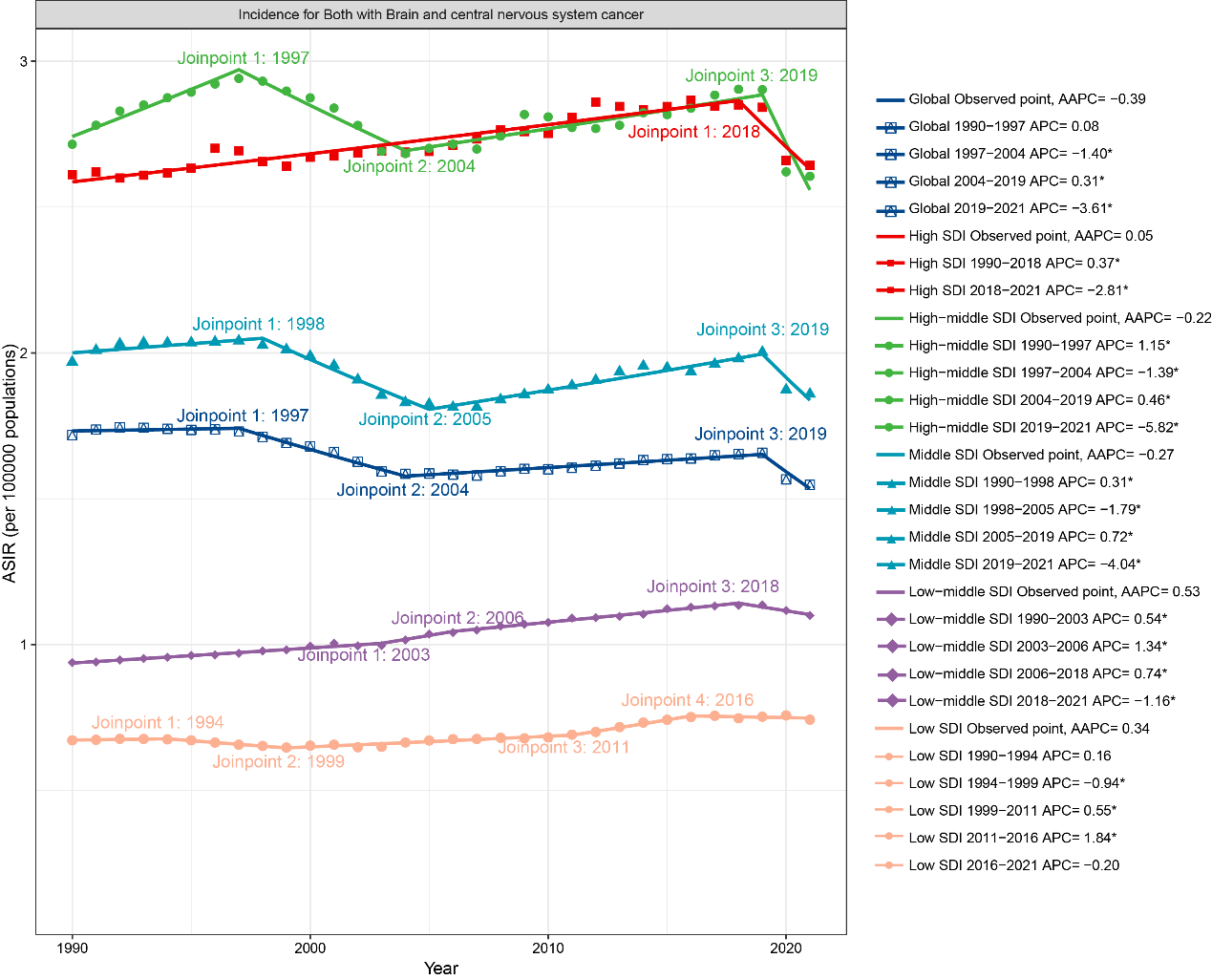
Supplementary Figure 1. Joinpoint regression analysis of incidence of CABCs at global and SDI levels (1990–2021). SDI, socio-demographic index. CABCs, childhood and adolescent brain and central nervous system cancers; ASPR, age-standardized prevalence rate; AAPC, average annual percentage changes; APC, annual percentage change.


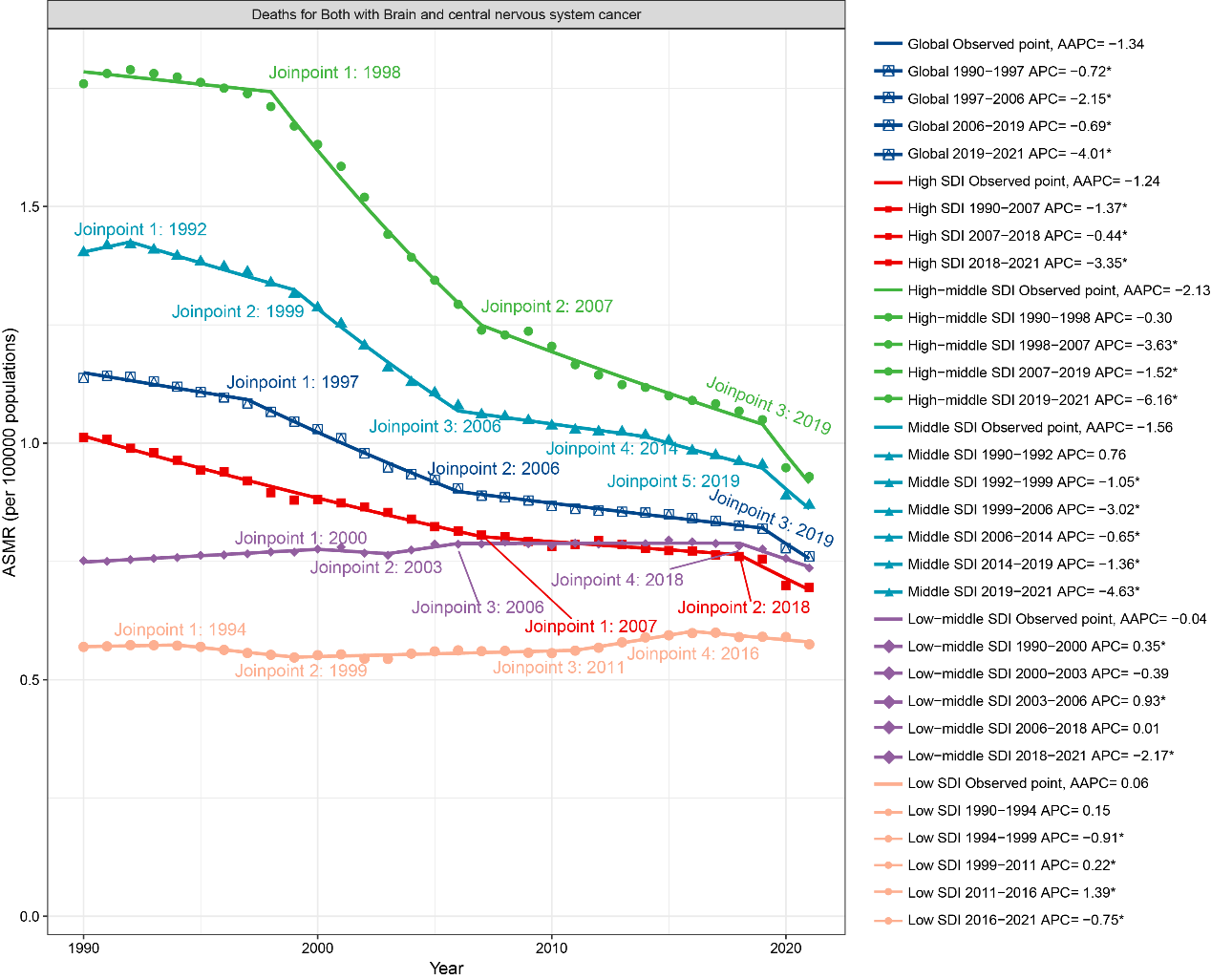


Supplementary Figure 2. Joinpoint regression analysis of mortality of CABCs at global and SDI levels (1990–2021). SDI, socio-demographic index. CABCs, childhood and adolescent brain and central nervous system cancers; ASPR, age-standardized prevalence rate; AAPC, average annual percentage changes; APC, annual percentage change.


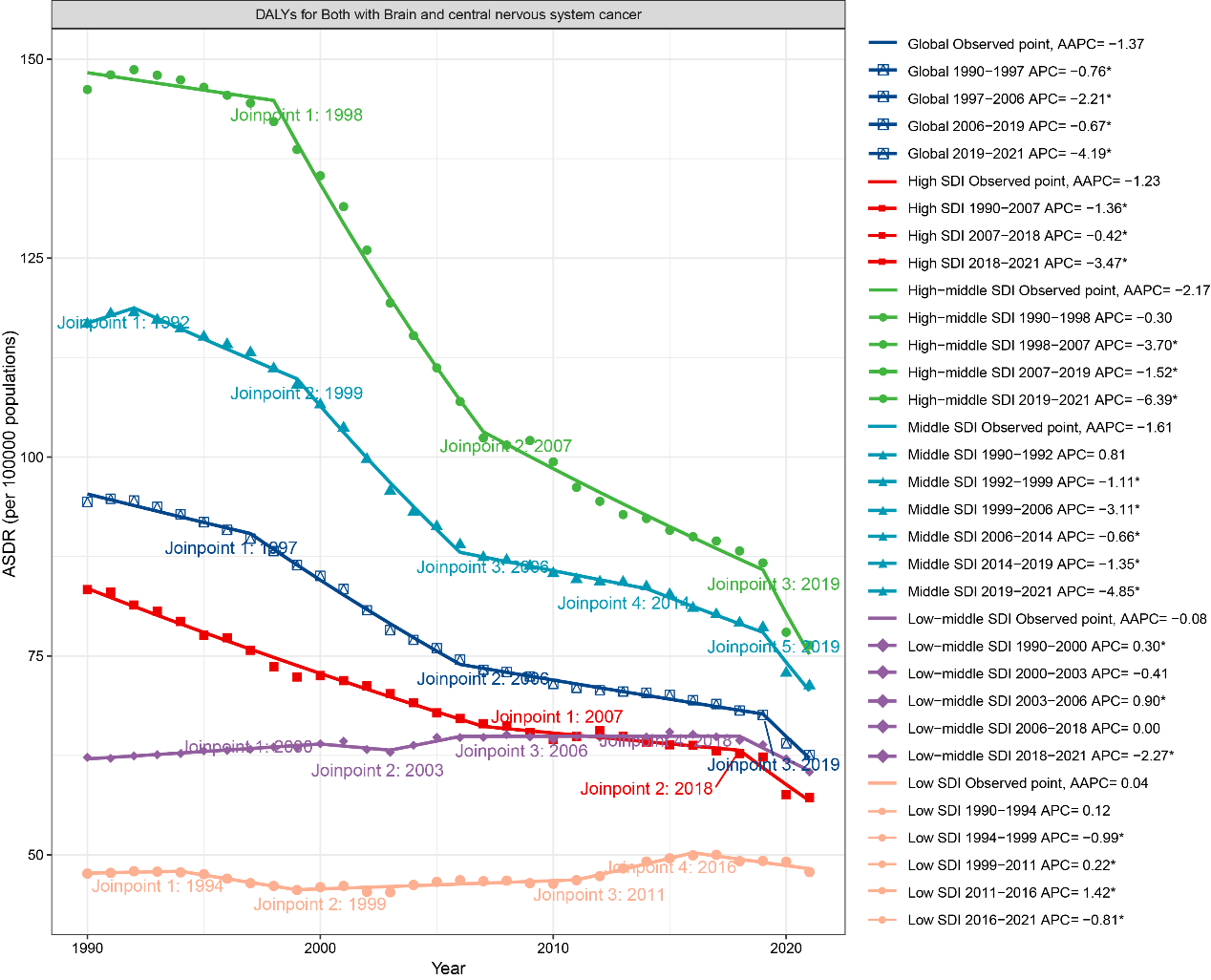


Supplementary Figure 3. Joinpoint regression analysis of DALYs of CABCs at global and SDI levels (1990–2021). SDI, socio-demographic index. CABCs, childhood and adolescent brain and central nervous system cancers; ASPR, age-standardized prevalence rate; AAPC, average annual percentage changes; APC, annual percentage change.


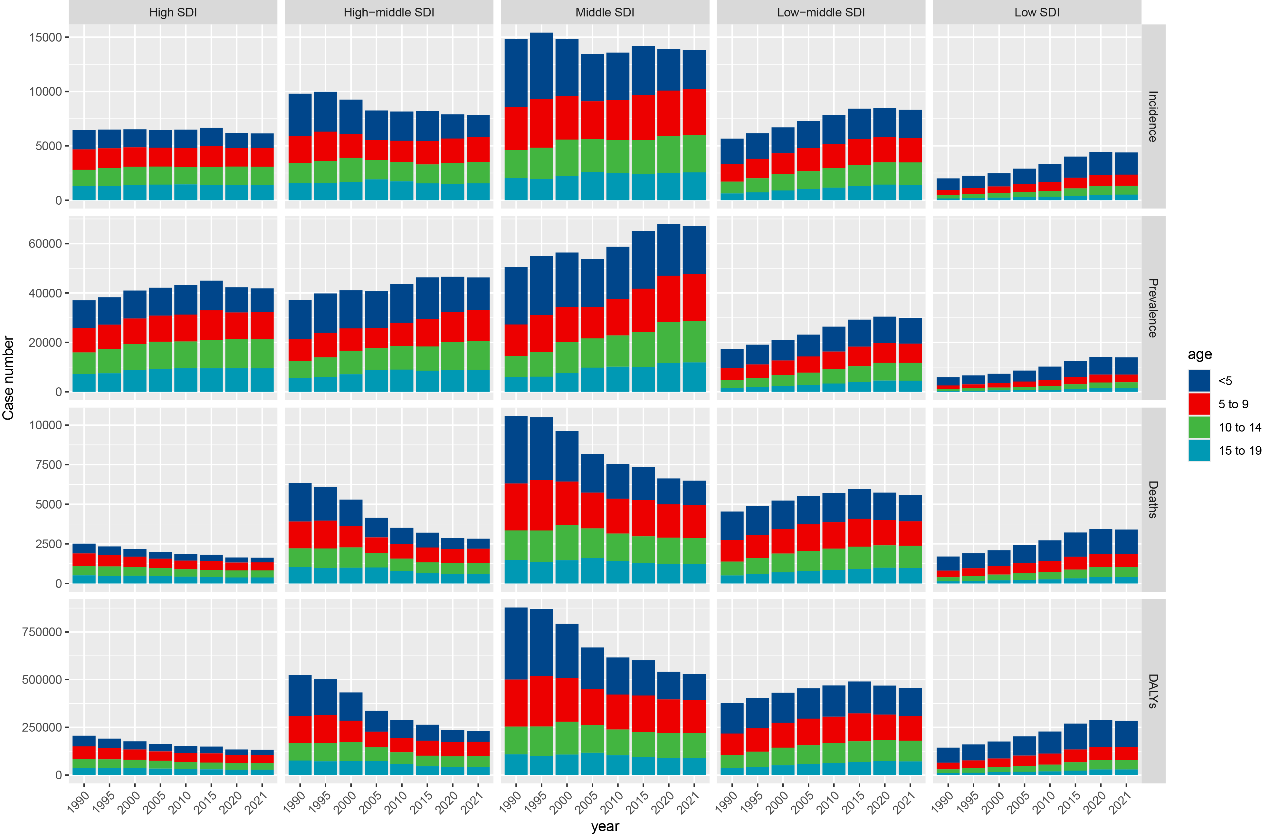


Supplementary Figure 4. The number of incidence, prevalence, deaths and DALYs of CABCs at SDI levels in different age groups (1990–2021). SDI, socio-demographic index. CABCs, childhood and adolescent brain and central nervous system cancers; ASPR, age-standardized prevalence rate; DALYs, disability adjusted life years.


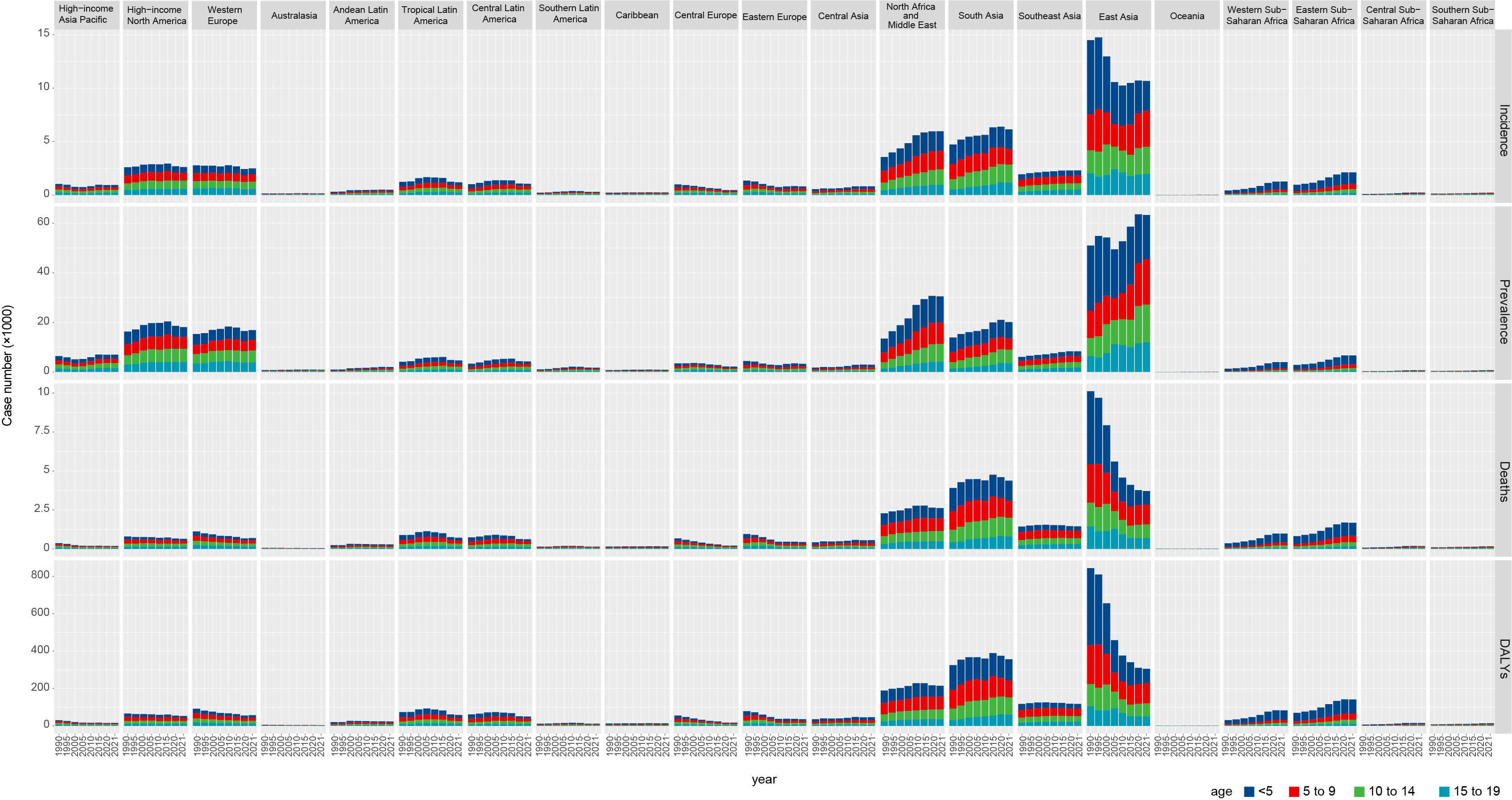


Supplementary Figure 5. The number of incidence, prevalence, deaths and DALYs of CABCs at regional levels in different age groups (1990–2021). SDI, socio-demographic index; CABCs, childhood and adolescent brain and central nervous system cancers; ASPR, age-standardized prevalence rate; DALYs, disability adjusted life years.


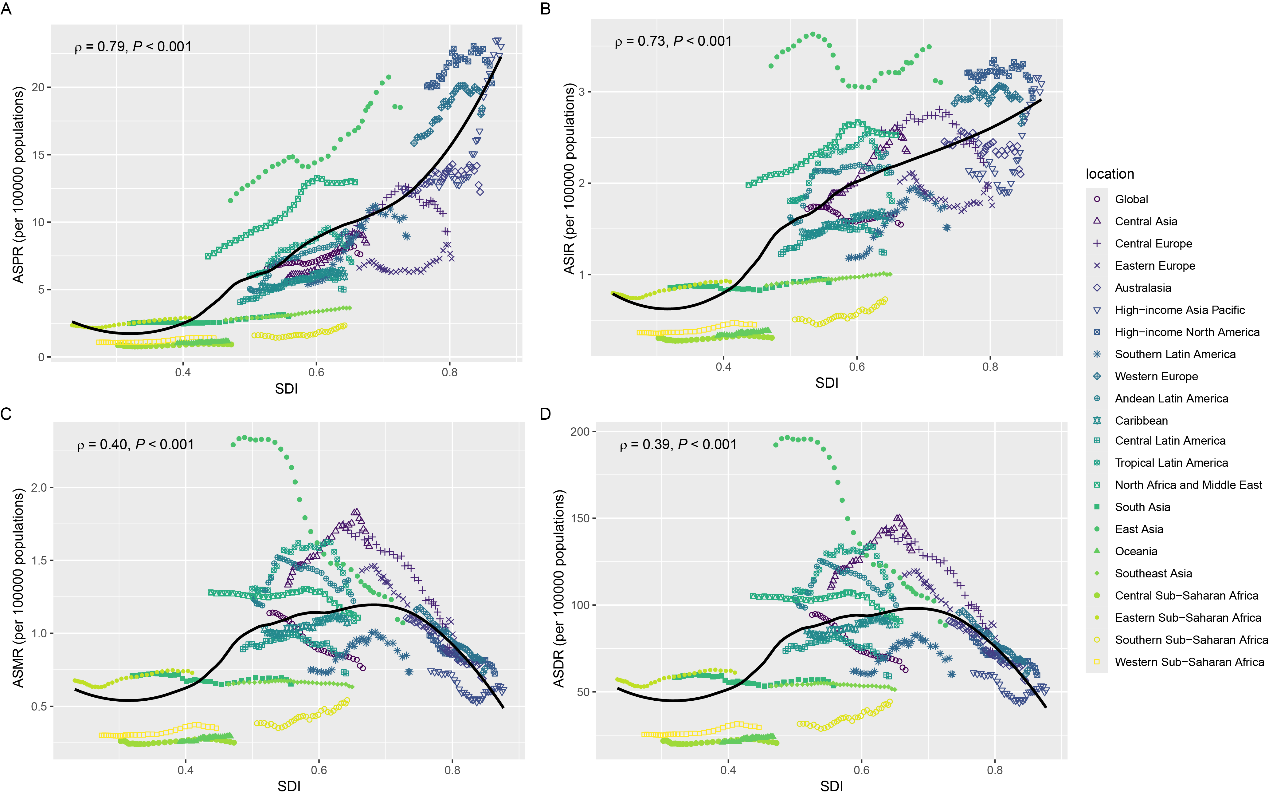


Supplementary Figure 6. The relative of ASR metrics and SDI in global and regional levels of CABCs from1990 to 2021. (A) ASPR, (B) ASIR, (C) ASMR, (D) ASDR. SDI, socio-demographic index. The black trend line indicates the association (positive/negative correlation). Regions are distinguished by color. The correlation coefficient (ρ) and its significance (p-value < 0.001) are shown in each panel's top-right corner (e.g., ρ = 0.79 indicates a strong positive correlation). CABCs, childhood and adolescent brain and central nervous system cancers; ASPR, age-standardized prevalence rate; ASIR, age-standardized incidence rate; ASMR, age-standardized mortality rate; ASDR, age-standardized DALY rate; DALYs, disability adjusted life years.


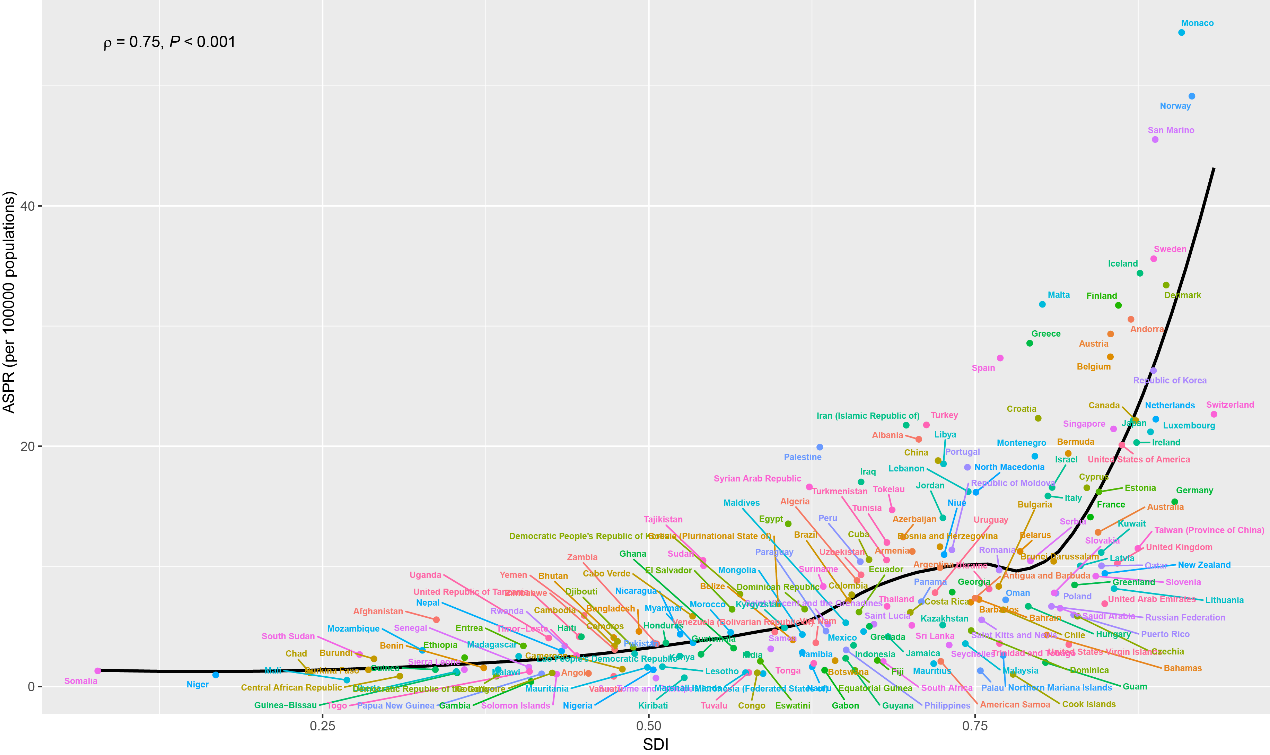


Supplementary Figure 7. The relative of ASPR and SDI in national level of CABCs in 2021. Each point represents a country. The black trend line, correlation coefficient (ρ), and p-value interpretation are as described above. SDI, socio-demographic index; CABCs, childhood and adolescent brain and central nervous system cancers; ASPR, age-standardized prevalence rate.


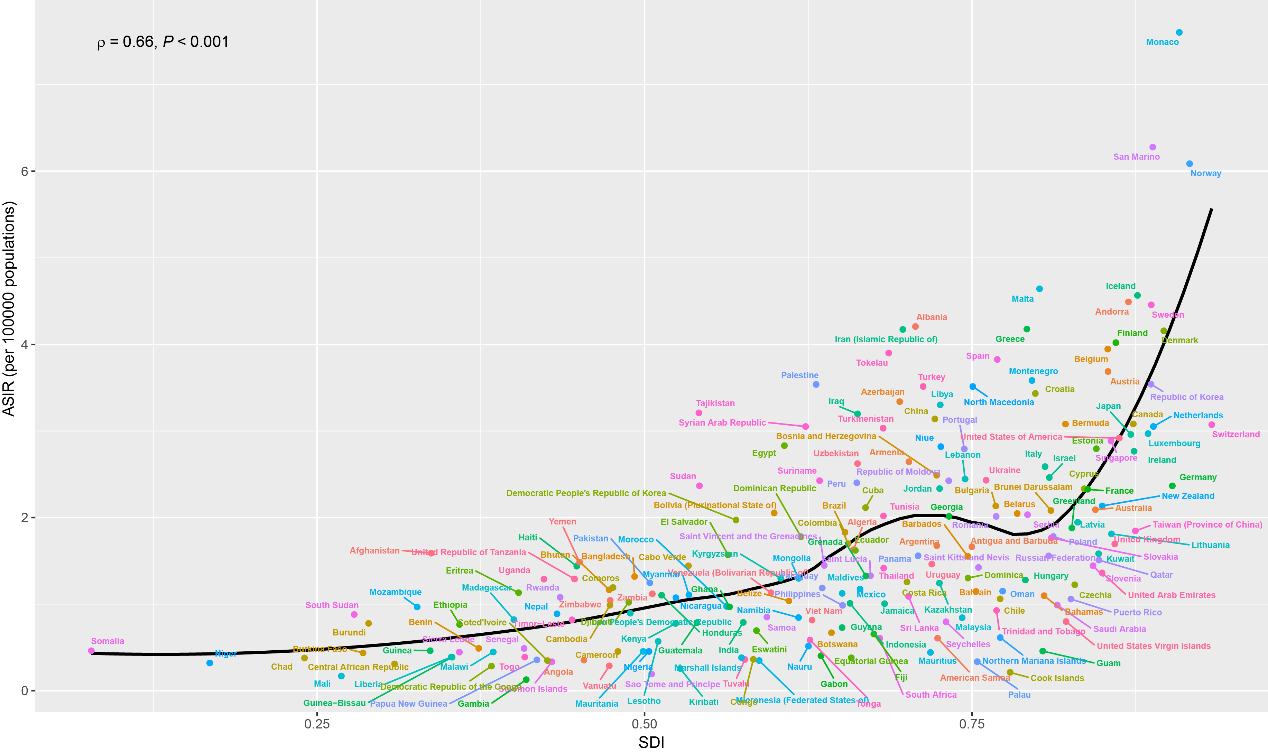


Supplementary Figure 8. The relative of ASIR and SDI in national level of CABCs in 2021. SDI, socio-demographic index. Each point represents a country. The black trend line, correlation coefficient (ρ), and p-value interpretation are as described above. CABCs, childhood and adolescent brain and central nervous system cancers; ASIR, age-standardized incidence rate.


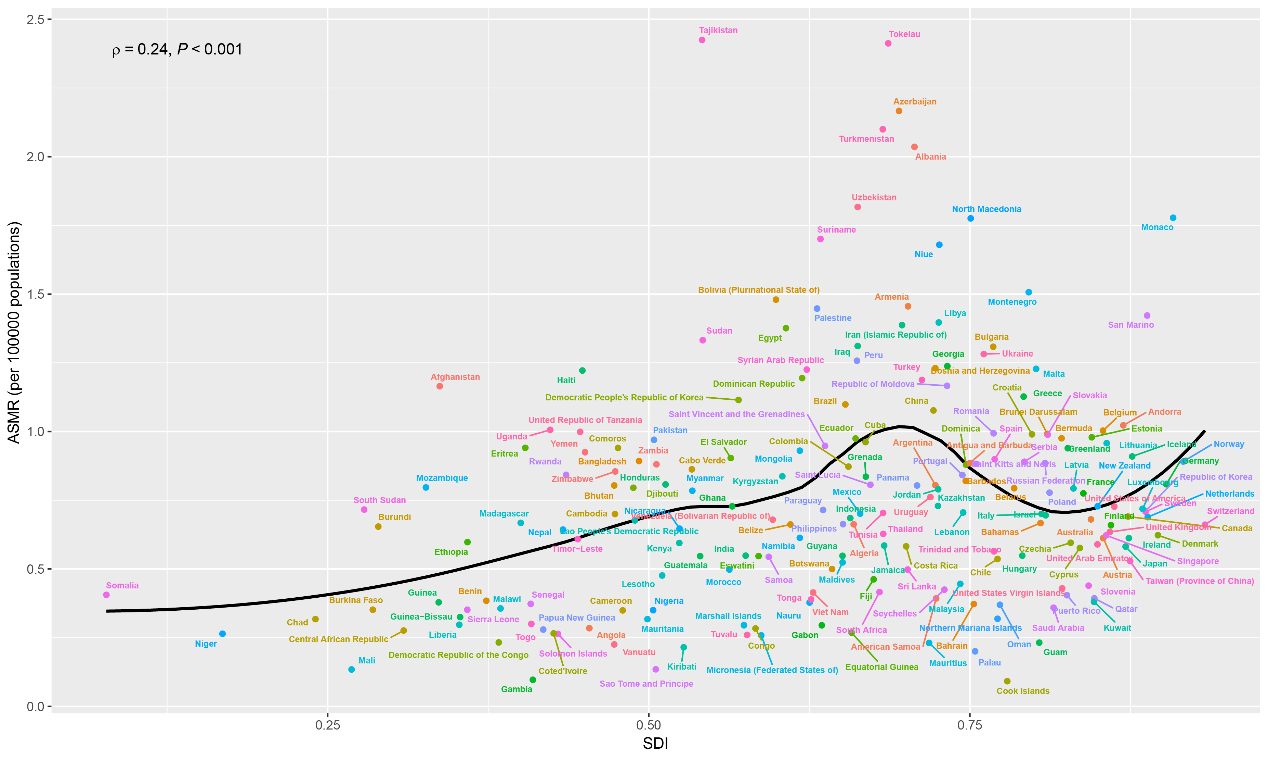


Supplementary Figure 9. The relative of ASMR and SDI in national level of CABCs in 2021. SDI, socio-demographic index; Each point represents a country. The black trend line, correlation coefficient (ρ), and p-value interpretation are as described above. CABCs, childhood and adolescent brain and central nervous system cancers; ASMR, age-standardized mortality rate.


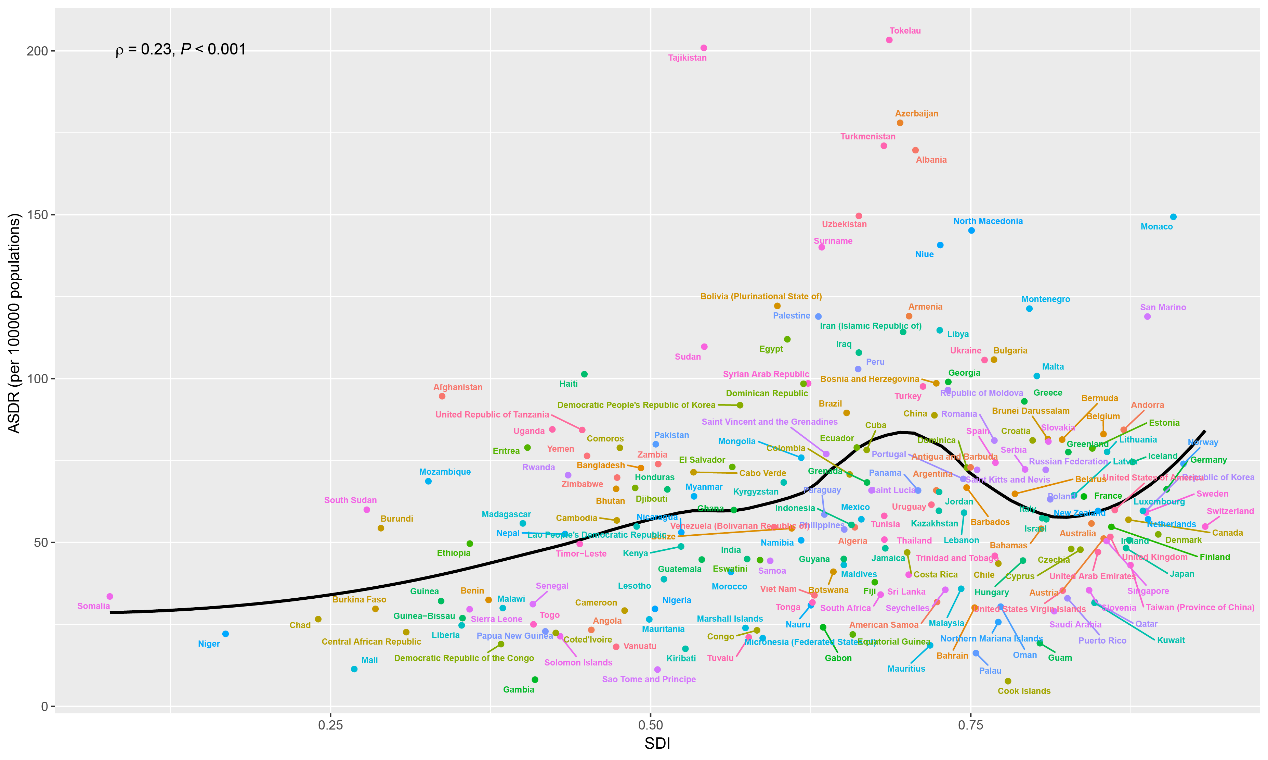


Supplementary Figure 10. The relative of ASDR and SDI in national level of CABCs in 2021. SDI, socio-demographic index. Each point represents a country. The black trend line, correlation coefficient (ρ), and p-value interpretation are as described above. CABCs, childhood and adolescent brain and central nervous system cancers; ASDR, age-standardized DALY rate; DALYs, disability adjusted life years.


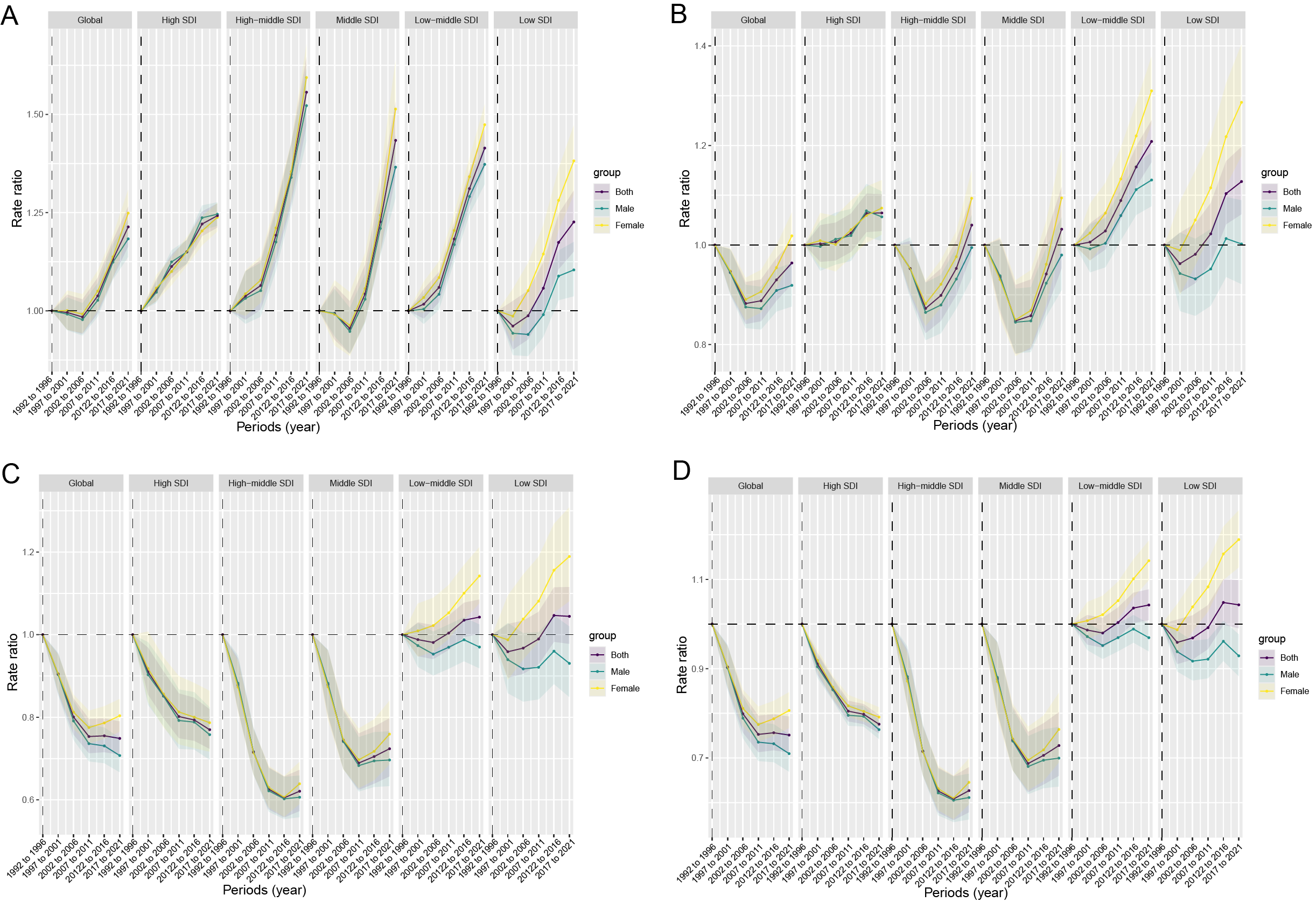


Supplementary Figure 11. Periods-specific trends in ASR metrics of CABCs by location and gender. (A) ASPR, (B) ASIR, (C) ASMR, (D) ASDR. SDI, socio-demographic index. Solid lines trace ASR estimates over successive 5-year periods—purple = both sexes, blue = males, yellow = females—with shaded ribbons indicating 95% UIs. Vertical dashed lines demarcate SDI quintiles (low to high), and a horizontal dashed line at RR = 1 marks the reference level. This layout highlights temporal changes in disease burden by development level and sex. CABCs, childhood and adolescent brain and central nervous system cancers; ASPR, age-standardized prevalence rate; ASIR, age-standardized incidence rate; ASMR, age-standardized mortality rate; ASDR, age-standardized DALY rate; DALYs, disability adjusted life years.


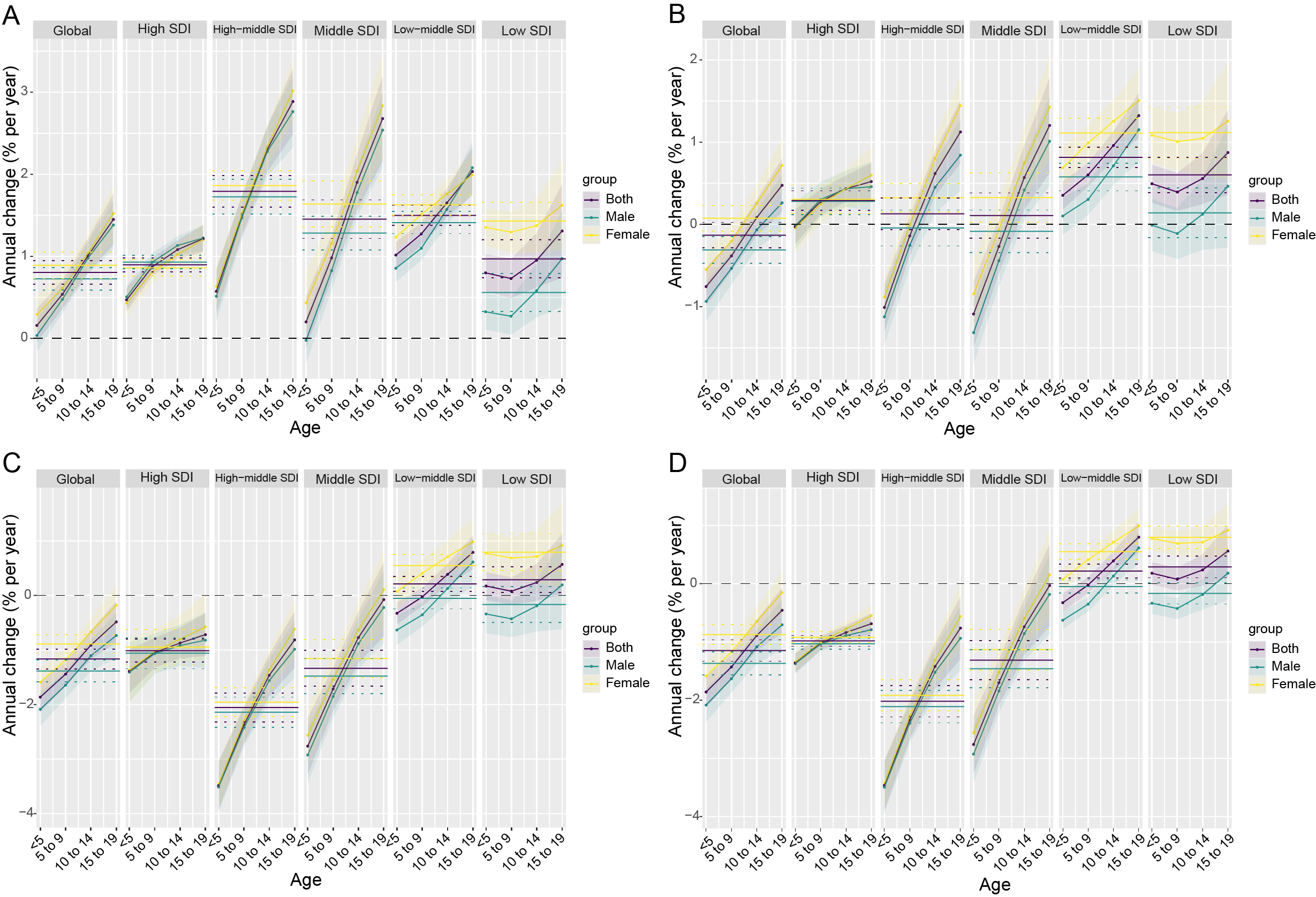


Supplementary Figure 12. Age-annual change trends in ASR metrics of CABCs by location and gender in 2021. (A) ASPR, (B) ASIR, (C) ASMR, (D) ASDR. SDI, socio-demographic index. Within each SDI facet (Global, High, High-middle, Middle, Low-middle, Low), solid lines (purple = both sexes, blue = males, yellow = females) show point estimates, with shaded ribbons for 95% UIs. Horizontal dashed lines in matching colors denote the overall average annual change for each sex-region combination; a black dashed line at 0% indicates no net change. This presentation emphasizes how annual trends vary by age, sex, and development level. CABCs, childhood and adolescent brain and central nervous system cancers; ASPR, age-standardized rates for prevalence; ASIR, age-standardized rates for incidence; ASMR, age-standardized mortality rate; ASDR, age-standardized DALY rate; DALYs, disability adjusted life years.


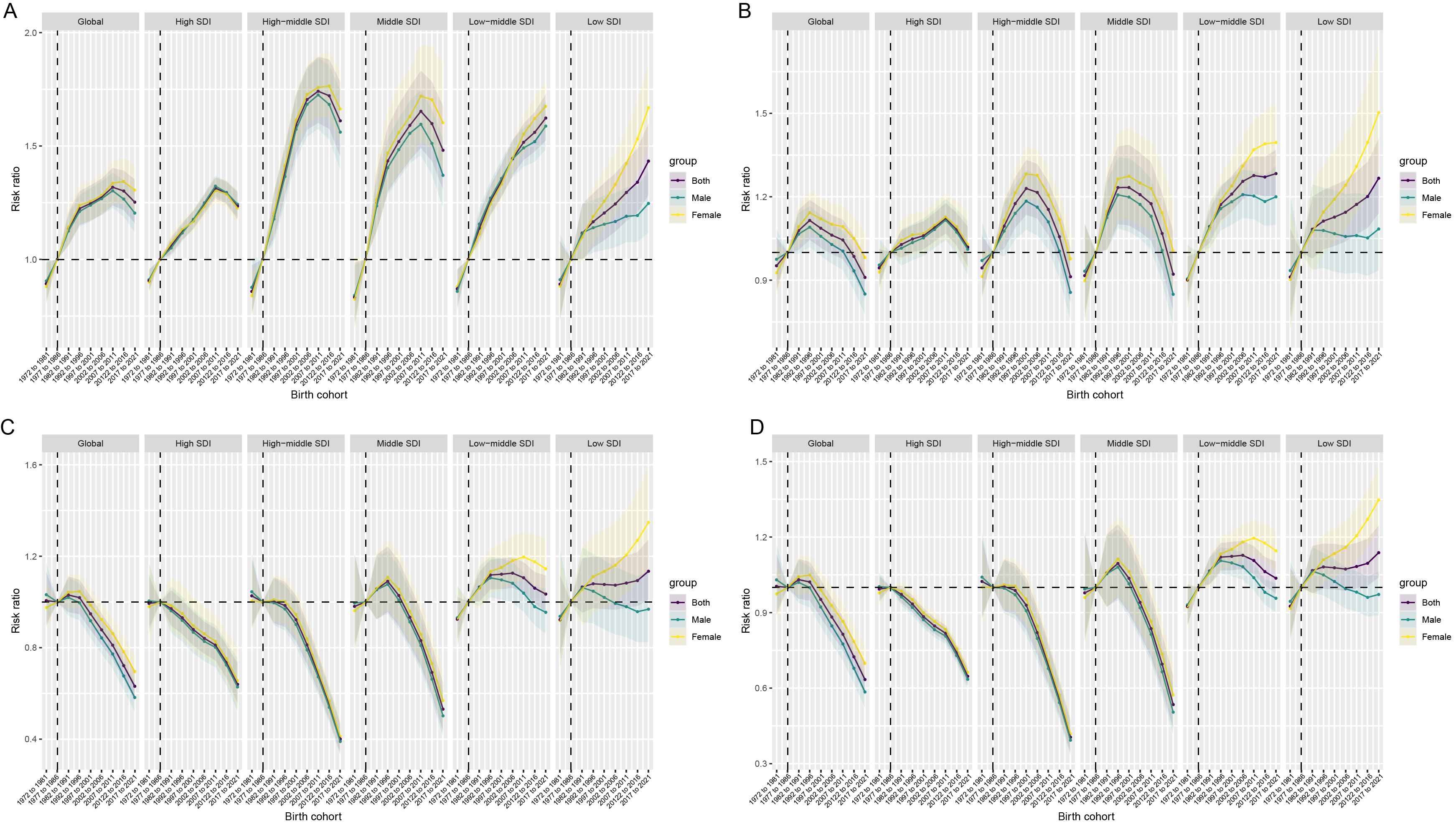


Supplementary Figure 13. The cohort effect in ASR metrics of CABCs by location and gender. (A) ASPR, (B) ASIR, (C) ASMR, (D) ASDR. In each SDI facet, solid lines (purple = both sexes, blue = males, yellow = females) trace rate ratio estimates for successive 5-year birth cohorts, with shaded bands indicating 95% UIs. A horizontal dashed line at RR = 1 marks the reference risk level, and a vertical dashed line indicates the reference cohort (1975–79). This layout illustrates generational shifts in disease burden across development settings and by sex. SDI, socio-demographic index; CABCs, childhood and adolescent brain and central nervous system cancers; ASPR, age-standardized prevalence rate; ASIR, age-standardized incidence rate; ASMR, age-standardized mortality rate; ASDR, age-standardized DALY rate; DALYs, disability adjusted life years.


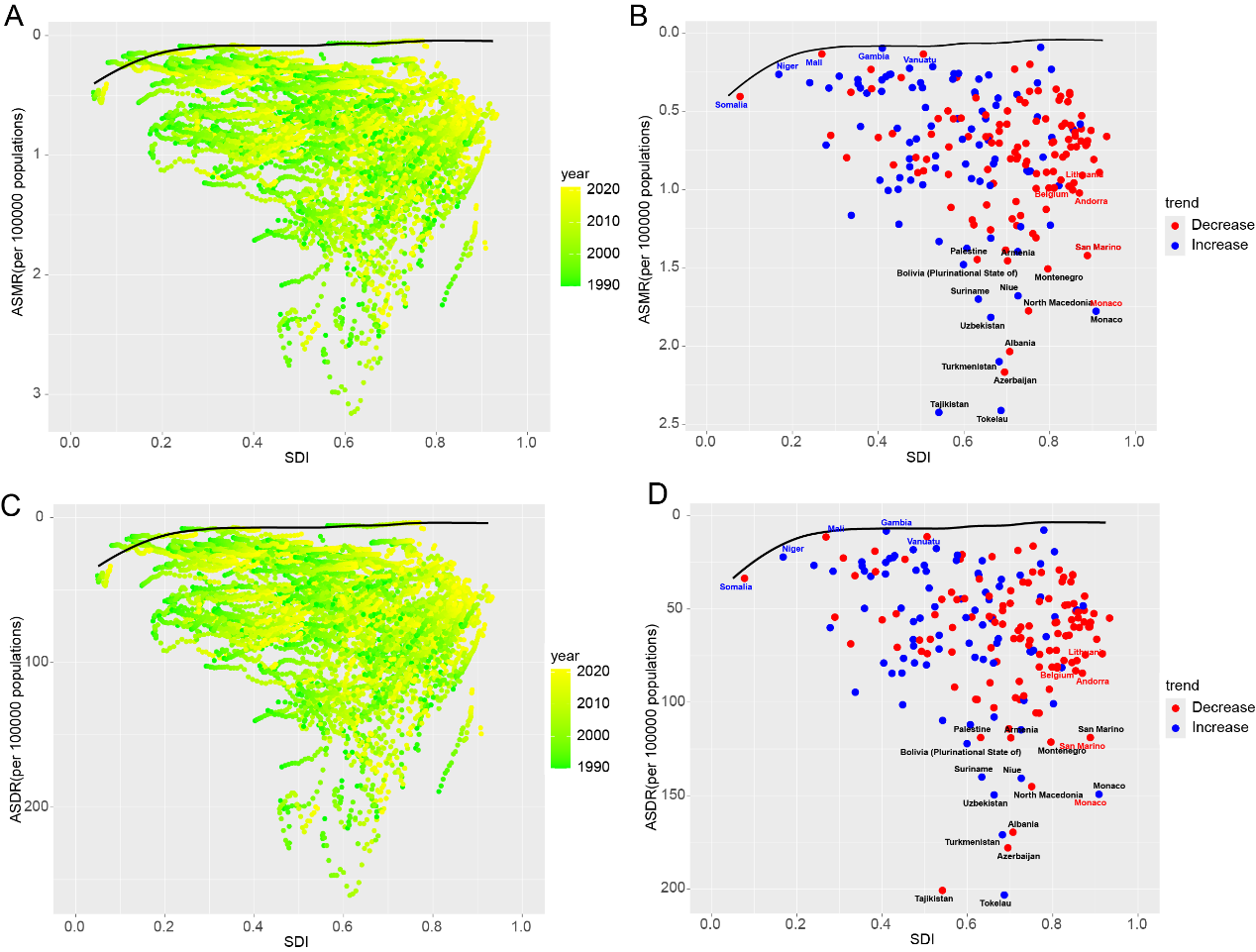


Supplementary Figure 14. Frontier analysis of ASMR and ASDR of CABCs. (A, C) Frontier analysis based on SDI and ASMR/ASDR of CABCs from 1990 to 2021. Color scale represents the years from 1990 depicted in green to 2016 depicted in yellow. The frontier is delineated in solid black color. (B, D) Frontier analysis based on SDI and ASMR/ASDR of CABCs in 2021. The top 15 countries with the largest effective difference (largest CABCs mortality/DALYs gap from the frontier) are labeled in black; examples of frontier countries with low SDI (<0.5) and low effective difference are labeled in blue, and examples of countries and territories with high SDI (>0.85) and relatively high effective difference for their level of development are labeled in red. Red dots indicate a decrease in ASMR/ASDR from 1990 to 2021; blue dots indicate an increase in ASMR/ASDR between 1990 and 2021. SDI, socio-demographic index. CABCs, childhood and adolescent brain and central nervous system cancers. ASMR, age-standardized mortality rate. ASDR, age-standardized DALY rate. DALYs, disability adjusted life years.

## Supplementary Table 1. Incidence, Deaths and DALYs of CABCs between 1990 and 2021 at the Global and Regional Levels

| Measure | Location | Case number in 1990 | ASR in 1990 (per 100000 populations) | Case number in 2021 | ASR in 2021(per 100000 populations) |
| --- | --- | --- | --- | --- | --- |
| Incidence | Global | 38816.6 (29857.6 to 48138.7) | 1.7 (1.3 to 2.1) | 40534.5 (32865.1 to 49430.4) | 1.5 (1.3 to 1.9) |
| Incidence | High SDI | 6470.5 (6137.0 to 6829.6) | 2.6 (2.5 to 2.8) | 6139.9 (5636.1 to 6661.7) | 2.6 (2.4 to 2.9) |
| Incidence | High-middle SDI | 9797.7 (7700.3 to 11847.4) | 2.7 (2.1 to 3.3) | 7848.4 (6207.5 to 10056.2) | 2.6 (2.1 to 3.4) |
| Incidence | Middle SDI | 14842.3 (10480.3 to 18328.7) | 2.0 (1.4 to 2.4) | 13806.3 (10579.5 to 17356.4) | 1.9 (1.4 to 2.3) |
| Incidence | Low-middle SDI | 5667.1 (3888.1 to 8615.6) | 0.9 (0.6 to 1.4) | 8316.2 (6359.1 to 10500.2) | 1.1 (0.8 to 1.4) |
| Incidence | Low SDI | 2007.2 (1189.9 to 3752.0) | 0.7 (0.4 to 1.2) | 4394.9 (2967.1 to 5858.9) | 0.7 (0.5 to 1.0) |
| Incidence | Andean Latin America | 310.3 (214.0 to 484.7) | 1.6 (1.1 to 2.5) | 499.0 (350.5 to 709.5) | 2.1 (1.5 to 3.0) |
| Incidence | Australasia | 151.6 (125.8 to 184.5) | 2.5 (2.0 to 3.0) | 158.1 (115.9 to 211.1) | 2.1 (1.5 to 2.8) |
| Incidence | Caribbean | 220.0 (163.9 to 374.5) | 1.5 (1.1 to 2.5) | 240.0 (174.0 to 351.5) | 1.6 (1.1 to 2.3) |
| Incidence | Central Asia | 555.4 (433.7 to 714.5) | 1.7 (1.4 to 2.2) | 822.0 (666.4 to 1016.1) | 2.3 (1.9 to 2.9) |
| Incidence | Central Europe | 992.6 (898.8 to 1106.8) | 2.6 (2.3 to 2.9) | 459.4 (389.1 to 535.9) | 1.9 (1.6 to 2.3) |
| Incidence | Central Latin America | 1012.6 (929.8 to 1109.3) | 1.2 (1.1 to 1.3) | 1056.7 (882.2 to 1273.0) | 1.2 (1.0 to 1.5) |
| Incidence | Central Sub-Saharan Africa | 99.3 (51.2 to 206.3) | 0.3 (0.2 to 0.6) | 228.8 (141.8 to 344.6) | 0.3 (0.2 to 0.5) |
| Incidence | East Asia | 14486.3 (9711.9 to 18635.6) | 3.3 (2.2 to 4.2) | 10667.4 (7894.9 to 14713.6) | 3.1 (2.3 to 4.3) |
| Incidence | Eastern Europe | 1346.8 (1228.5 to 1491.2) | 2.0 (1.8 to 2.2) | 802.5 (717.6 to 898.0) | 1.8 (1.6 to 2.0) |
| Incidence | Eastern Sub-Saharan Africa | 963.1 (621.7 to 1645.2) | 0.8 (0.5 to 1.3) | 2123.4 (1438.7 to 3066.5) | 0.9 (0.6 to 1.3) |
| Incidence | High-income Asia Pacific | 1041.1 (855.9 to 1249.9) | 2.1 (1.8 to 2.6) | 941.5 (719.0 to 1199.4) | 3.1 (2.3 to 3.9) |
| Incidence | High-income North America | 2601.5 (2420.2 to 2791.7) | 3.2 (3.0 to 3.4) | 2619.1 (2364.8 to 2903.7) | 2.9 (2.6 to 3.3) |
| Incidence | North Africa and Middle East | 3557.4 (2366.7 to 5344.1) | 2.0 (1.3 to 3.0) | 5965.5 (4195.1 to 7734.3) | 2.5 (1.8 to 3.3) |
| Incidence | Oceania | 11.2 (5.4 to 18.6) | 0.3 (0.2 to 0.5) | 24.6 (13.0 to 39.8) | 0.4 (0.2 to 0.6) |
| Incidence | South Asia | 4726.4 (2817.6 to 7348.8) | 0.9 (0.5 to 1.3) | 6144.9 (4658.0 to 8286.5) | 0.9 (0.7 to 1.2) |
| Incidence | Southeast Asia | 1939.0 (1165.5 to 2827.0) | 0.9 (0.5 to 1.3) | 2303.5 (1607.2 to 2928.8) | 1.0 (0.7 to 1.3) |
| Incidence | Southern Latin America | 227.7 (181.2 to 292.6) | 1.2 (0.9 to 1.5) | 293.7 (222.1 to 393.3) | 1.5 (1.1 to 2.0) |
| Incidence | Southern Sub-Saharan Africa | 135.2 (98.8 to 190.2) | 0.5 (0.4 to 0.7) | 227.4 (162.4 to 300.5) | 0.7 (0.5 to 1.0) |
| Incidence | Tropical Latin America | 1230.0 (1046.4 to 1419.2) | 1.8 (1.5 to 2.1) | 1195.3 (988.5 to 1399.3) | 1.8 (1.5 to 2.1) |
| Incidence | Western Europe | 2774.6 (2556.4 to 3018.8) | 2.9 (2.6 to 3.1) | 2498.8 (2223.2 to 2806.8) | 2.7 (2.4 to 3.1) |
| Incidence | Western Sub-Saharan Africa | 434.6 (279.2 to 649.0) | 0.4 (0.2 to 0.5) | 1262.8 (612.2 to 1716.1) | 0.5 (0.2 to 0.6) |
| Deaths | Global | 25697.8 (19107.0 to 32741.4) | 1.1 (0.8 to 1.4) | 19922.5 (15752.1 to 24599.3) | 0.8 (0.6 to 0.9) |
| Deaths | High SDI | 2513.2 (2397.9 to 2622.1) | 1.0 (1.0 to 1.1) | 1622.6 (1517.1 to 1735.4) | 0.7 (0.6 to 0.7) |
| Deaths | High-middle SDI | 6350.0 (4962.9 to 7710.4) | 1.8 (1.4 to 2.1) | 2820.8 (2311.4 to 3530.9) | 0.9 (0.8 to 1.2) |
| Deaths | Middle SDI | 10577.9 (7437.2 to 13168.5) | 1.4 (1.0 to 1.7) | 6484.9 (4994.1 to 8033.8) | 0.9 (0.7 to 1.1) |
| Deaths | Low-middle SDI | 4536.0 (3081.2 to 6988.9) | 0.8 (0.5 to 1.2) | 5575.8 (4305.7 to 7079.6) | 0.7 (0.6 to 0.9) |
| Deaths | Low SDI | 1699.4 (1008.6 to 3188.5) | 0.6 (0.3 to 1.1) | 3402.5 (2266.2 to 4578.6) | 0.6 (0.4 to 0.8) |
| Deaths | Andean Latin America | 240.0 (167.3 to 371.0) | 1.3 (0.9 to 1.9) | 288.1 (211.1 to 389.0) | 1.2 (0.9 to 1.7) |
| Deaths | Australasia | 68.4 (61.2 to 75.9) | 1.1 (1.0 to 1.2) | 52.1 (44.1 to 61.1) | 0.7 (0.6 to 0.8) |
| Deaths | Caribbean | 153.6 (110.5 to 280.4) | 1.0 (0.7 to 1.9) | 161.4 (114.9 to 251.5) | 1.1 (0.8 to 1.7) |
| Deaths | Central Asia | 427.0 (336.2 to 545.1) | 1.3 (1.1 to 1.7) | 559.4 (455.3 to 688.2) | 1.6 (1.3 to 2.0) |
| Deaths | Central Europe | 672.8 (620.5 to 735.7) | 1.8 (1.6 to 1.9) | 212.2 (186.4 to 241.2) | 0.9 (0.8 to 1.0) |
| Deaths | Central Latin America | 737.5 (683.6 to 801.0) | 0.9 (0.8 to 1.0) | 617.8 (523.0 to 735.5) | 0.7 (0.6 to 0.9) |
| Deaths | Central Sub-Saharan Africa | 85.4 (44.6 to 176.1) | 0.3 (0.1 to 0.5) | 185.9 (116.1 to 278.8) | 0.3 (0.2 to 0.4) |
| Deaths | East Asia | 10113.7 (6746.0 to 13052.9) | 2.3 (1.5 to 3.0) | 3711.7 (2727.2 to 5062.2) | 1.1 (0.8 to 1.5) |
| Deaths | Eastern Europe | 957.5 (892.4 to 1034.2) | 1.4 (1.3 to 1.5) | 439.8 (399.0 to 482.1) | 1.0 (0.9 to 1.0) |
| Deaths | Eastern Sub-Saharan Africa | 816.3 (523.7 to 1412.8) | 0.7 (0.4 to 1.2) | 1678.3 (1135.1 to 2434.8) | 0.7 (0.5 to 1.1) |
| Deaths | High-income Asia Pacific | 369.7 (301.4 to 425.6) | 0.7 (0.6 to 0.9) | 190.1 (163.5 to 212.2) | 0.6 (0.5 to 0.7) |
| Deaths | High-income North America | 799.0 (777.5 to 821.0) | 1.0 (1.0 to 1.0) | 649.3 (603.6 to 696.4) | 0.7 (0.7 to 0.8) |
| Deaths | North Africa and Middle East | 2285.1 (1543.5 to 3467.0) | 1.3 (0.9 to 1.9) | 2623.7 (1867.0 to 3330.6) | 1.1 (0.8 to 1.4) |
| Deaths | Oceania | 8.6 (4.2 to 14.4) | 0.3 (0.1 to 0.4) | 18.8 (9.8 to 30.3) | 0.3 (0.2 to 0.5) |
| Deaths | South Asia | 3910.3 (2331.2 to 6127.4) | 0.7 (0.4 to 1.1) | 4377.3 (3316.8 to 5897.7) | 0.7 (0.5 to 0.9) |
| Deaths | Southeast Asia | 1433.6 (849.1 to 2132.4) | 0.7 (0.4 to 1.0) | 1451.1 (1009.6 to 1846.4) | 0.6 (0.4 to 0.8) |
| Deaths | Southern Latin America | 144.5 (122.1 to 170.9) | 0.7 (0.6 to 0.9) | 143.9 (120.9 to 170.0) | 0.7 (0.6 to 0.9) |
| Deaths | Southern Sub-Saharan Africa | 101.8 (74.8 to 142.7) | 0.4 (0.3 to 0.5) | 169.1 (121.0 to 222.8) | 0.5 (0.4 to 0.7) |
| Deaths | Tropical Latin America | 892.1 (760.5 to 1025.6) | 1.3 (1.1 to 1.5) | 717.8 (595.7 to 834.8) | 1.1 (0.9 to 1.3) |
| Deaths | Western Europe | 1122.6 (1083.0 to 1162.9) | 1.2 (1.1 to 1.2) | 694.4 (644.8 to 748.9) | 0.8 (0.7 to 0.8) |
| Deaths | Western Sub-Saharan Africa | 358.4 (232.5 to 548.7) | 0.3 (0.2 to 0.5) | 980.3 (475.7 to 1329.4) | 0.3 (0.2 to 0.5) |
| DALYs | Global | 2131492.7 (1580756.0 to 2723340.0) | 94.4 (70.0 to 120.5) | 1632939.7 (1288499.1 to 2019216.2) | 62.6 (49.3 to 77.5) |
| DALYs | High SDI | 205846.5 (196394.0 to 214896.5) | 83.3 (79.5 to 87.0) | 132616.3 (123793.3 to 141956.8) | 57.2 (53.3 to 61.3) |
| DALYs | High-middle SDI | 525020.0 (408891.3 to 637848.3) | 146.2 (113.6 to 177.8) | 230556.9 (188562.2 to 289040.9) | 76.3 (62.3 to 96.0) |
| DALYs | Middle SDI | 878233.2 (615548.3 to 1095621.9) | 116.7 (81.7 to 145.7) | 529028.1 (406362.1 to 656859.1) | 71.3 (54.6 to 88.7) |
| DALYs | Low-middle SDI | 377290.4 (255875.8 to 584813.3) | 62.3 (42.3 to 95.9) | 455732.8 (351158.7 to 579588.9) | 60.4 (46.5 to 76.9) |
| DALYs | Low SDI | 143346.8 (85024.9 to 270443.8) | 47.6 (28.3 to 88.6) | 283698.9 (188405.9 to 382650.3) | 47.8 (31.8 to 64.5) |
| DALYs | Andean Latin America | 19911.3 (13832.9 to 30944.1) | 104.3 (72.5 to 161.8) | 23519.0 (17191.3 to 31854.6) | 100.0 (72.9 to 135.6) |
| DALYs | Australasia | 5586.9 (4999.4 to 6194.8) | 90.8 (81.4 to 100.6) | 4253.5 (3590.6 to 4993.2) | 56.5 (47.6 to 66.4) |
| DALYs | Caribbean | 12718.1 (9085.3 to 23568.8) | 85.0 (60.8 to 157.3) | 13269.9 (9386.5 to 20823.3) | 88.5 (62.3 to 139.8) |
| DALYs | Central Asia | 35410.7 (27801.9 to 45398.7) | 109.8 (86.7 to 139.9) | 46143.9 (37458.3 to 56872.3) | 131.1 (106.6 to 161.4) |
| DALYs | Central Europe | 55012.9 (50669.3 to 60219.2) | 144.1 (132.5 to 157.9) | 17149.7 (15040.4 to 19525.8) | 73.1 (63.9 to 83.5) |
| DALYs | Central Latin America | 61010.5 (56491.2 to 66335.4) | 73.7 (68.2 to 80.1) | 49913.6 (42092.1 to 59670.2) | 58.9 (49.4 to 70.9) |
| DALYs | Central Sub-Saharan Africa | 7153.5 (3695.7 to 14919.6) | 21.8 (11.6 to 43.8) | 15232.9 (9486.2 to 22917.7) | 20.5 (12.8 to 30.7) |
| DALYs | East Asia | 843690.5 (560233.9 to 1090208.8) | 192.2 (127.3 to 248.5) | 304984.3 (223208.4 to 416932.6) | 88.4 (64.4 to 121.1) |
| DALYs | Eastern Europe | 78362.3 (72987.4 to 84648.3) | 117.3 (109.2 to 126.7) | 35712.6 (32357.5 to 39191.9) | 78.0 (70.3 to 85.9) |
| DALYs | Eastern Sub-Saharan Africa | 69454.1 (44533.8 to 120735.5) | 57.1 (36.7 to 98.2) | 140813.4 (94876.3 to 205143.8) | 61.1 (41.2 to 88.8) |
| DALYs | High-income Asia Pacific | 29967.5 (24478.7 to 34480.2) | 61.1 (50.0 to 70.4) | 15644.6 (13440.1 to 17525.9) | 51.2 (43.8 to 57.7) |
| DALYs | High-income North America | 65855.4 (64025.8 to 67783.3) | 81.4 (79.1 to 83.7) | 53063.0 (49243.4 to 56980.7) | 59.6 (55.2 to 64.1) |
| DALYs | North Africa and Middle East | 188781.8 (127086.8 to 288011.6) | 105.3 (71.1 to 160.0) | 214697.5 (152438.0 to 272770.0) | 90.8 (64.4 to 115.3) |
| DALYs | Oceania | 703.5 (343.9 to 1190.2) | 20.7 (10.1 to 34.9) | 1543.0 (809.0 to 2496.6) | 24.0 (12.5 to 38.6) |
| DALYs | South Asia | 324621.3 (193097.4 to 511330.1) | 58.5 (34.9 to 91.6) | 356399.5 (269500.0 to 481473.2) | 53.6 (40.4 to 72.7) |
| DALYs | Southeast Asia | 117052.5 (68927.2 to 175611.0) | 53.2 (31.3 to 80.0) | 117172.8 (81426.0 to 149494.0) | 51.3 (35.6 to 65.7) |
| DALYs | Southern Latin America | 11919.0 (10100.7 to 14072.7) | 61.8 (52.4 to 73.0) | 11658.3 (9795.9 to 13784.9) | 60.1 (50.4 to 71.2) |
| DALYs | Southern Sub-Saharan Africa | 8392.4 (6166.9 to 11814.2) | 31.6 (23.2 to 44.3) | 13820.3 (9873.7 to 18237.7) | 44.4 (31.7 to 58.6) |
| DALYs | Tropical Latin America | 73789.8 (62755.7 to 85001.3) | 108.4 (91.9 to 125.1) | 58307.4 (48126.1 to 68134.6) | 88.3 (72.6 to 103.5) |
| DALYs | Western Europe | 91540.8 (88271.1 to 94879.9) | 95.6 (92.2 to 99.1) | 56753.3 (52599.6 to 61226.9) | 62.3 (57.6 to 67.3) |
| DALYs | Western Sub-Saharan Africa | 30558.0 (19810.1 to 46958.0) | 25.5 (16.6 to 38.7) | 82887.5 (39945.5 to 112540.3) | 29.5 (14.3 to 39.9) |

SDI, socio-demographic index; DALYs, disability adjusted life years.

## Supplementary Table 2. Prevalence, Incidence, Deaths and DALYs of CABCs between 1990 and 2021 at the National level

| Measure | Location | Case number in 1990 | ASR in 1990 (per 100000 populations) | Case number in 2021 | ASR in 2021(per 100000 populations) | AAPC between 1990-2021 (95% CI) |
| --- | --- | --- | --- | --- | --- | --- |
| Prevalence | Afghanistan | 193.6 (75.9 to 619.3) | 3.5 (1.4 to 10.7) | 1003.8 (496.4 to 1812.3) | 5.6 (2.8 to 9.9) | 1.57 (0.84 to 2.31) |
| Prevalence | Albania | 163.2 (102.5 to 234.2) | 11.3 (7.1 to 16.1) | 121.1 (63.7 to 200.4) | 20.6 (10.8 to 34) | 1.8 (1.38 to 2.22) |
| Prevalence | Algeria | 818.2 (400.6 to 1313.2) | 6 (2.9 to 9.6) | 1489.3 (686.9 to 2471.8) | 8.8 (4.1 to 14.6) | 1.29 (1.14 to 1.45) |
| Prevalence | American Samoa | 0.3 (0.2 to 0.5) | 1.4 (0.9 to 2.3) | 0.4 (0.2 to 0.6) | 2.1 (1.2 to 3.3) | 1.3 (0.72 to 1.89) |
| Prevalence | Andorra | 4.9 (2.4 to 9.4) | 38.6 (18.6 to 76.4) | 4.4 (2.3 to 7.5) | 30.6 (16.4 to 52.8) | -0.68 (-1.29 to -0.06) |
| Prevalence | Angola | 59.3 (24.9 to 152.6) | 1 (0.4 to 2.4) | 208.3 (114.9 to 348.8) | 1.1 (0.6 to 1.8) | 0.44 (0.12 to 0.77) |
| Prevalence | Antigua and Barbuda | 0.9 (0.7 to 1.4) | 4 (2.8 to 5.8) | 1.7 (1.2 to 2.4) | 7.3 (5.1 to 10.5) | 2.08 (1.59 to 2.56) |
| Prevalence | Argentina | 853.9 (591.9 to 1204.1) | 6.6 (4.6 to 9.3) | 1337.5 (877.5 to 2026.9) | 9.9 (6.5 to 15) | 1.55 (1.25 to 1.85) |
| Prevalence | Armenia | 114.2 (76.2 to 161.7) | 8.5 (5.7 to 12) | 85.2 (59.3 to 121.6) | 11.2 (7.8 to 16.1) | 0.77 (-0.22 to 1.77) |
| Prevalence | Australia | 666.5 (516.3 to 855.5) | 13.2 (10.2 to 16.9) | 798.7 (538.4 to 1147.5) | 12.8 (8.6 to 18.4) | -0.09 (-0.63 to 0.46) |
| Prevalence | Austria | 477.3 (323.8 to 690.8) | 26.2 (17.7 to 37.9) | 513.3 (340.4 to 718.3) | 29.3 (19.5 to 41) | 0.43 (-0.3 to 1.17) |
| Prevalence | Azerbaijan | 277.7 (165.9 to 437.6) | 8.8 (5.3 to 13.9) | 376.4 (224 to 602.6) | 12.4 (7.4 to 20) | 1.11 (0.73 to 1.49) |
| Prevalence | Bahamas | 3.1 (2.2 to 4.3) | 2.9 (2 to 4.1) | 4.9 (3.2 to 7) | 4.3 (2.8 to 6.2) | 1.44 (-0.02 to 2.92) |
| Prevalence | Bahrain | 9.4 (5.9 to 15.1) | 4.6 (2.9 to 7.4) | 29.2 (15.8 to 51.5) | 7.2 (3.9 to 12.8) | 1.5 (0.89 to 2.12) |
| Prevalence | Bangladesh | 1924.4 (1011.1 to 4108.9) | 3.1 (1.6 to 6.4) | 2772.2 (1703.9 to 4346.4) | 4.6 (2.8 to 7.2) | 1.33 (1.01 to 1.65) |
| Prevalence | Barbados | 5.3 (3.7 to 7.3) | 6.3 (4.4 to 8.7) | 4.6 (2.9 to 7) | 7 (4.4 to 10.7) | 0.27 (-0.7 to 1.26) |
| Prevalence | Belarus | 147.6 (105 to 201.8) | 4.7 (3.4 to 6.5) | 226.5 (142.7 to 343.1) | 11.2 (7 to 17.1) | 2.81 (0.76 to 4.89) |
| Prevalence | Belgium | 536.3 (372.5 to 772.2) | 22.1 (15.3 to 31.8) | 697 (459.3 to 1029.8) | 27.4 (18.1 to 40.6) | 0.34 (-1.29 to 2) |
| Prevalence | Belize | 4.2 (3 to 5.8) | 4 (2.8 to 5.5) | 6.4 (4.7 to 8.7) | 3.9 (2.8 to 5.3) | -0.08 (-0.63 to 0.48) |
| Prevalence | Benin | 29.6 (18.2 to 45.6) | 0.9 (0.5 to 1.4) | 125.4 (54.4 to 210.5) | 1.5 (0.7 to 2.6) | 1.86 (1.36 to 2.37) |
| Prevalence | Bermuda | 1.2 (0.7 to 1.9) | 7.4 (4.4 to 11.8) | 2.1 (1.3 to 3.3) | 19.4 (11.8 to 30.5) | 3.15 (2.79 to 3.51) |
| Prevalence | Bhutan | 7.9 (2.4 to 15) | 2.3 (0.7 to 4.4) | 10 (5.8 to 16.9) | 4.1 (2.3 to 6.9) | 1.93 (1.68 to 2.17) |
| Prevalence | Bolivia (Plurinational State of) | 148.5 (86 to 267.5) | 4.3 (2.5 to 7.7) | 310.5 (186.7 to 482.1) | 6.9 (4.1 to 10.7) | 1.56 (1.47 to 1.65) |
| Prevalence | Bosnia and Herzegovina | 114.6 (78.6 to 169.7) | 7.8 (5.3 to 11.6) | 79.9 (49.6 to 122.8) | 11.6 (7.2 to 17.9) | 1.25 (0.56 to 1.94) |
| Prevalence | Botswana | 9.4 (5.7 to 15.1) | 1.3 (0.8 to 2) | 19.5 (11.7 to 31.4) | 2.1 (1.3 to 3.5) | 1.76 (1.31 to 2.22) |
| Prevalence | Brazil | 4048.1 (3424.4 to 4691) | 6.2 (5.2 to 7.1) | 4540.3 (3715.2 to 5388.6) | 7.1 (5.8 to 8.5) | 0.43 (0.06 to 0.8) |
| Prevalence | Brunei Darussalam | 9.4 (5.8 to 15.4) | 8.2 (5 to 13.4) | 13.1 (7.9 to 20.5) | 10.4 (6.3 to 16.3) | 0.74 (0.45 to 1.04) |
| Prevalence | Bulgaria | 201.7 (161.1 to 250) | 8.8 (7.1 to 10.9) | 107.7 (77.2 to 146.1) | 8.3 (6 to 11.3) | -0.05 (-2.46 to 2.41) |
| Prevalence | Burkina Faso | 49.2 (29.9 to 78.3) | 0.8 (0.5 to 1.2) | 193.8 (86.3 to 303.7) | 1.4 (0.6 to 2.2) | 1.98 (1.67 to 2.3) |
| Prevalence | Burundi | 91.3 (50.8 to 154.5) | 2.6 (1.4 to 4.4) | 172.8 (79.6 to 300.2) | 2.3 (1.1 to 4) | -0.4 (-0.88 to 0.08) |
| Prevalence | Cabo Verde | 4.4 (2.5 to 8.9) | 2.2 (1.2 to 4.3) | 10.8 (5.7 to 18.7) | 5.9 (3.1 to 10.2) | 3.31 (3.01 to 3.61) |
| Prevalence | Cambodia | 135.8 (67.4 to 273.5) | 2.3 (1.2 to 4.6) | 222.5 (131.8 to 349.1) | 3.4 (2 to 5.3) | 1.21 (1.08 to 1.35) |
| Prevalence | Cameroon | 58.1 (36.7 to 87.5) | 0.9 (0.5 to 1.3) | 249.7 (111.3 to 403.2) | 1.4 (0.6 to 2.3) | 1.65 (1.05 to 2.24) |
| Prevalence | Canada | 1737.7 (1196.5 to 2451.7) | 22.8 (15.7 to 32.2) | 1838 (1221.4 to 2684.9) | 22.1 (14.7 to 32.4) | -0.05 (-0.74 to 0.64) |
| Prevalence | Central African Republic | 13.5 (6.6 to 31.6) | 0.8 (0.4 to 1.9) | 25.1 (13 to 51.7) | 0.9 (0.4 to 1.7) | -0.11 (-0.34 to 0.12) |
| Prevalence | Chad | 24.9 (14.7 to 44) | 0.6 (0.4 to 1.1) | 134.4 (72.3 to 211) | 1.1 (0.6 to 1.8) | 2.02 (1.7 to 2.35) |
| Prevalence | Chile | 117.1 (85.9 to 159.8) | 2.2 (1.6 to 3.1) | 312.1 (203.2 to 488.9) | 6.4 (4.2 to 10) | 3.62 (2.27 to 4.99) |
| Prevalence | China | 49603 (33010.5 to 64073.3) | 11.7 (7.8 to 15.1) | 62313 (45832.5 to 86130) | 18.8 (13.8 to 26.1) | 1.42 (1.17 to 1.68) |
| Prevalence | Colombia | 713.2 (559.5 to 887.3) | 4.7 (3.7 to 5.9) | 1107.9 (758.2 to 1582.9) | 7.6 (5.2 to 10.9) | 1.51 (0.99 to 2.04) |
| Prevalence | Comoros | 8.3 (4 to 12.4) | 2.9 (1.4 to 4.4) | 11.5 (6.5 to 18.4) | 3.8 (2.1 to 6) | 0.63 (-0.86 to 2.14) |
| Prevalence | Congo | 13.2 (7 to 24.3) | 1 (0.5 to 1.8) | 28.2 (16.7 to 43.7) | 1.1 (0.7 to 1.8) | 0.57 (0.06 to 1.09) |
| Prevalence | Cook Islands | 0 (0 to 0.1) | 0.4 (0.2 to 0.6) | 0 (0 to 0.1) | 1 (0.5 to 1.8) | 3.54 (2.31 to 4.78) |
| Prevalence | Costa Rica | 72.5 (54.7 to 95.4) | 5.1 (3.8 to 6.7) | 85.6 (58.7 to 123.2) | 6.2 (4.2 to 8.9) | 0.71 (-0.35 to 1.77) |
| Prevalence | Coted'Ivoire | 68.3 (33 to 103) | 0.9 (0.4 to 1.3) | 170.8 (70.7 to 285.6) | 1.1 (0.5 to 1.9) | 0.77 (0.25 to 1.3) |
| Prevalence | Croatia | 210.7 (147.2 to 299.2) | 15.9 (11.1 to 22.6) | 181.9 (115.2 to 281.5) | 22.3 (14.1 to 34.6) | 0.95 (-0.82 to 2.75) |
| Prevalence | Cuba | 278.4 (197.8 to 393.1) | 7.8 (5.5 to 11) | 253.7 (171.9 to 361.1) | 10.6 (7.1 to 15.1) | 1.04 (0.43 to 1.65) |
| Prevalence | Cyprus | 26.3 (16 to 43.7) | 10.2 (6.2 to 17) | 47.1 (25.2 to 80.4) | 16.5 (8.9 to 28.2) | 1.51 (0.95 to 2.08) |
| Prevalence | Czechia | 172.8 (137.8 to 216.2) | 5.8 (4.7 to 7.3) | 130.4 (89.5 to 185.6) | 5.9 (4 to 8.3) | -0.01 (-0.65 to 0.63) |
| Prevalence | Democratic People's Republic of Korea | 598.1 (322.4 to 976.4) | 7.5 (4 to 12.2) | 489.5 (266.5 to 821.5) | 7.7 (4.2 to 12.9) | 0.09 (-0.07 to 0.25) |
| Prevalence | Democratic Republic of the Congo | 194.4 (95 to 427.8) | 0.8 (0.4 to 1.8) | 412.2 (238.1 to 663.4) | 0.9 (0.5 to 1.4) | 0.04 (-0.19 to 0.28) |
| Prevalence | Denmark | 408.4 (280.1 to 586) | 33.9 (23.4 to 48.7) | 430.8 (298.3 to 577.3) | 33.4 (23.1 to 44.9) | 0.17 (-0.52 to 0.86) |
| Prevalence | Djibouti | 7.8 (4.7 to 11.7) | 3.4 (2 to 5.1) | 17.4 (9.5 to 29.1) | 3.3 (1.8 to 5.5) | 0.08 (-0.67 to 0.82) |
| Prevalence | Dominica | 0.7 (0.4 to 1.1) | 2.2 (1.3 to 3.3) | 0.8 (0.5 to 1.3) | 4.7 (2.7 to 7.2) | 2.5 (2.35 to 2.66) |
| Prevalence | Dominican Republic | 192.5 (112.4 to 309) | 5.5 (3.2 to 8.8) | 247.5 (151.8 to 378) | 6.4 (3.9 to 9.8) | 0.56 (0.32 to 0.8) |
| Prevalence | Ecuador | 46 (34.3 to 59.9) | 0.9 (0.7 to 1.2) | 409.4 (282.5 to 586.8) | 6.2 (4.3 to 8.9) | 6.68 (4.35 to 9.07) |
| Prevalence | Egypt | 1548.2 (949.3 to 3227.3) | 5.4 (3.3 to 11.2) | 6354.5 (3890 to 9846.1) | 13.5 (8.3 to 21) | 3.08 (2.65 to 3.5) |
| Prevalence | El Salvador | 111.8 (77.8 to 161.8) | 4 (2.8 to 5.8) | 153.3 (91.6 to 243.4) | 6.4 (3.8 to 10.2) | 1.44 (1.02 to 1.85) |
| Prevalence | Equatorial Guinea | 2.2 (1 to 4.6) | 0.9 (0.4 to 1.8) | 10.2 (4.4 to 20.9) | 1.3 (0.6 to 2.7) | 1.53 (1.08 to 1.98) |
| Prevalence | Eritrea | 48.1 (28.2 to 81.1) | 2.3 (1.3 to 3.8) | 111.1 (63.2 to 179.4) | 3.4 (1.9 to 5.4) | 1.37 (1.07 to 1.67) |
| Prevalence | Estonia | 38.2 (28.2 to 51) | 8.4 (6.2 to 11.2) | 45.6 (30.2 to 67.1) | 16.2 (10.7 to 23.9) | 2.31 (0.22 to 4.44) |
| Prevalence | Eswatini | 6.5 (4 to 11.3) | 1.3 (0.8 to 2.3) | 11.2 (6.8 to 17.3) | 2.1 (1.3 to 3.2) | 1.46 (1.23 to 1.7) |
| Prevalence | Ethiopia | 557.8 (257.1 to 1848.4) | 1.7 (0.8 to 5.6) | 1380.6 (754.3 to 2642) | 2.4 (1.3 to 4.6) | 0.97 (0.66 to 1.29) |
| Prevalence | Fiji | 7.4 (3.1 to 11.7) | 2 (0.8 to 3.2) | 7.6 (3.9 to 12.2) | 2.2 (1.1 to 3.5) | 0.23 (-0.21 to 0.68) |
| Prevalence | Finland | 331.5 (219.9 to 480.9) | 26.1 (17.3 to 37.9) | 372.7 (258.9 to 509.3) | 31.7 (22 to 43.4) | 0.47 (0.08 to 0.87) |
| Prevalence | France | 1476.3 (1099.4 to 2005) | 9.3 (6.9 to 12.6) | 2175.6 (1517.9 to 3036.7) | 14.1 (9.8 to 19.8) | 1.32 (0.79 to 1.86) |
| Prevalence | Gabon | 5.1 (2.9 to 8.6) | 1 (0.6 to 1.6) | 11.2 (6.1 to 18.7) | 1.4 (0.7 to 2.3) | 1.03 (0.48 to 1.57) |
| Prevalence | Gambia | 2.2 (1.1 to 3.4) | 0.3 (0.2 to 0.5) | 5.4 (2.4 to 8.9) | 0.4 (0.2 to 0.7) | 0.56 (-0.69 to 1.83) |
| Prevalence | Georgia | 65.1 (41.1 to 99.6) | 3.6 (2.3 to 5.6) | 72 (50.4 to 99.1) | 7.9 (5.5 to 10.8) | 2.71 (1.37 to 4.07) |
| Prevalence | Germany | 1770.9 (1380.8 to 2314.2) | 10.4 (8.1 to 13.5) | 2433.7 (1769.7 to 3329.2) | 15.4 (11.2 to 21) | 1.28 (0.81 to 1.75) |
| Prevalence | Ghana | 313.8 (187.2 to 479.4) | 3.6 (2.1 to 5.5) | 526 (285 to 859.9) | 3.2 (1.7 to 5.2) | -0.39 (-0.61 to -0.17) |
| Prevalence | Greece | 856 (593.1 to 1210.7) | 30.9 (21.5 to 43.6) | 540.6 (377.9 to 787.3) | 28.6 (19.9 to 41.5) | -0.13 (-1.93 to 1.71) |
| Prevalence | Greenland | 2.4 (1.3 to 3.8) | 13.4 (7.2 to 20.9) | 1.3 (0.8 to 2.1) | 8.4 (5 to 14) | -1.47 (-1.81 to -1.13) |
| Prevalence | Grenada | 1.4 (0.9 to 2.1) | 3.2 (2.1 to 4.8) | 1.5 (1 to 2.1) | 5 (3.5 to 7) | 1.57 (0.65 to 2.51) |
| Prevalence | Guam | 0.8 (0.5 to 1.3) | 1.6 (1 to 2.4) | 1 (0.4 to 1.6) | 2 (0.9 to 3.4) | 0.93 (-0.92 to 2.82) |
| Prevalence | Guatemala | 140.7 (117.3 to 168.3) | 2.7 (2.2 to 3.2) | 173.5 (129.3 to 233.1) | 2.7 (2 to 3.6) | -0.06 (-0.76 to 0.65) |
| Prevalence | Guinea | 57.5 (30.8 to 87.3) | 1.5 (0.8 to 2.2) | 112 (43.5 to 195.6) | 1.4 (0.6 to 2.4) | -0.07 (-0.58 to 0.43) |
| Prevalence | Guinea-Bissau | 5.3 (3.1 to 8.7) | 0.8 (0.5 to 1.4) | 13 (6 to 21.2) | 1.1 (0.5 to 1.8) | 1.16 (-0.21 to 2.55) |
| Prevalence | Guyana | 4.4 (3.3 to 5.9) | 1.2 (0.9 to 1.5) | 6.5 (4.7 to 9) | 2.3 (1.7 to 3.2) | 2.47 (0.24 to 4.75) |
| Prevalence | Haiti | 131.4 (56.1 to 485.4) | 3.7 (1.6 to 13.3) | 233 (115 to 536.8) | 4.1 (2 to 9.5) | 0.43 (0.06 to 0.8) |
| Prevalence | Honduras | 121.3 (81.7 to 170.2) | 4.3 (2.9 to 6.1) | 155.2 (76.2 to 266.2) | 3.6 (1.8 to 6.2) | -0.58 (-0.68 to -0.48) |
| Prevalence | Hungary | 200.5 (158.7 to 255.7) | 7.1 (5.6 to 9) | 126.4 (87.8 to 177.9) | 6.7 (4.6 to 9.4) | -0.31 (-1.37 to 0.76) |
| Prevalence | Iceland | 34.9 (23.7 to 50.8) | 41.3 (28 to 60.2) | 31.6 (18.9 to 48.3) | 34.4 (20.6 to 52.5) | -0.23 (-1.45 to 1.02) |
| Prevalence | India | 10044.5 (6073 to 15289.6) | 2.4 (1.5 to 3.6) | 13027.9 (9494.5 to 17978.6) | 2.7 (1.9 to 3.7) | 0.39 (0.09 to 0.7) |
| Prevalence | Indonesia | 2170.4 (1221.9 to 3521.2) | 2.5 (1.4 to 4.1) | 3089.5 (1997.7 to 4355.1) | 3.4 (2.2 to 4.9) | 1.09 (0.9 to 1.28) |
| Prevalence | Iran (Islamic Republic of) | 3318.5 (1851.3 to 5399.9) | 10.3 (5.7 to 16.6) | 5547.9 (2760.6 to 9014.8) | 21.8 (10.7 to 35.8) | 2.46 (2.04 to 2.88) |
| Prevalence | Iraq | 764.8 (443.6 to 1426.4) | 7.3 (4.2 to 13.5) | 2927.3 (1692.7 to 4935.2) | 17 (9.8 to 28.8) | 2.68 (2.31 to 3.05) |
| Prevalence | Ireland | 255.3 (175.7 to 365.5) | 19.4 (13.4 to 27.8) | 267.4 (172.4 to 396) | 20.3 (13.1 to 30.1) | 0.1 (-0.74 to 0.95) |
| Prevalence | Israel | 325 (227.9 to 464.3) | 16.4 (11.5 to 23.4) | 559 (369.7 to 827.5) | 16.6 (11 to 24.5) | 0.24 (-1.02 to 1.52) |
| Prevalence | Italy | 2111.2 (1496 to 2912.6) | 16.2 (11.5 to 22.3) | 1652.2 (1114.2 to 2395) | 15.9 (10.6 to 23) | -0.4 (-0.84 to 0.04) |
| Prevalence | Jamaica | 40.6 (26.3 to 57.7) | 3.8 (2.4 to 5.4) | 32.3 (21.4 to 47.7) | 4.1 (2.8 to 6.1) | 0.4 (-0.74 to 1.56) |
| Prevalence | Japan | 4574.4 (3729.9 to 5638.7) | 14.5 (11.8 to 17.9) | 4675.7 (3288.2 to 6251.7) | 22.2 (15.6 to 29.6) | 1.46 (0.98 to 1.95) |
| Prevalence | Jordan | 150.9 (93.9 to 233.2) | 7.1 (4.4 to 11) | 658.8 (365.6 to 1106.4) | 14 (7.7 to 23.5) | 2.22 (1.93 to 2.52) |
| Prevalence | Kazakhstan | 291.8 (215.9 to 384.6) | 4.4 (3.2 to 5.8) | 346.9 (245.3 to 486.2) | 5.1 (3.6 to 7.2) | 0.51 (-0.3 to 1.32) |
| Prevalence | Kenya | 272 (178.8 to 478.4) | 1.9 (1.2 to 3.3) | 610.5 (411.4 to 981.7) | 2.5 (1.7 to 4.1) | 0.98 (0.44 to 1.53) |
| Prevalence | Kiribati | 0.2 (0.1 to 0.4) | 0.7 (0.3 to 1.1) | 0.4 (0.2 to 0.7) | 0.7 (0.3 to 1.3) | 0.23 (-0.2 to 0.67) |
| Prevalence | Kuwait | 83.8 (57.1 to 121.6) | 11.8 (8.1 to 17.1) | 121.3 (74.3 to 187.9) | 11.2 (6.8 to 17.3) | -0.19 (-1.61 to 1.25) |
| Prevalence | Kyrgyzstan | 41.5 (29.9 to 59.1) | 1.9 (1.4 to 2.7) | 138.9 (99.4 to 186.9) | 4.9 (3.5 to 6.6) | 3.17 (2.25 to 4.1) |
| Prevalence | Lao People's Democratic Republic | 46 (21.7 to 118.6) | 2 (1 to 5) | 81.1 (46.5 to 129.6) | 2.7 (1.6 to 4.4) | 1.08 (0.78 to 1.39) |
| Prevalence | Latvia | 55.8 (41.1 to 75.4) | 7.5 (5.5 to 10.1) | 38.4 (24.8 to 58.4) | 10 (6.5 to 15.3) | 1.19 (-0.74 to 3.15) |
| Prevalence | Lebanon | 84.1 (47.3 to 138.4) | 6.2 (3.5 to 10.1) | 261.8 (121.4 to 473.2) | 16.2 (7.4 to 29.5) | 3.21 (2.83 to 3.59) |
| Prevalence | Lesotho | 8.5 (5.3 to 14.2) | 1 (0.6 to 1.7) | 13.6 (8.2 to 22.5) | 1.6 (1 to 2.7) | 1.67 (1.28 to 2.06) |
| Prevalence | Liberia | 11.8 (6.6 to 20.7) | 0.8 (0.4 to 1.3) | 34.8 (14.8 to 56.6) | 1.2 (0.5 to 2) | 1.45 (0.39 to 2.52) |
| Prevalence | Libya | 234.2 (124.9 to 375.6) | 10.1 (5.4 to 16.2) | 362.2 (178.2 to 625.7) | 18.5 (8.9 to 32.3) | 1.93 (1.51 to 2.35) |
| Prevalence | Lithuania | 62 (47.6 to 80.2) | 5.7 (4.4 to 7.3) | 43.5 (30.8 to 60.2) | 8.1 (5.8 to 11.3) | 1.47 (0.2 to 2.75) |
| Prevalence | Luxembourg | 20.3 (14.2 to 29.3) | 23.1 (16.1 to 33.3) | 28.5 (18.1 to 43.7) | 21.2 (13.5 to 32.5) | -0.84 (-1.13 to -0.55) |
| Prevalence | Madagascar | 184.2 (119.5 to 283.6) | 2.5 (1.6 to 3.8) | 377 (215 to 598.5) | 2.5 (1.4 to 4) | 0.03 (-0.4 to 0.46) |
| Prevalence | Malawi | 84.3 (45.1 to 133.8) | 1.3 (0.7 to 2.1) | 144.1 (63.2 to 249.7) | 1.4 (0.6 to 2.4) | 0.21 (-0.22 to 0.64) |
| Prevalence | Malaysia | 206.2 (98.7 to 333.7) | 2.5 (1.2 to 4) | 370.2 (186.7 to 592.8) | 3.6 (1.8 to 5.7) | 1.33 (0.68 to 1.99) |
| Prevalence | Maldives | 3.2 (1.7 to 7.7) | 2.4 (1.3 to 5.7) | 6.9 (4 to 11.1) | 5.3 (3.1 to 8.6) | 2.55 (1.98 to 3.13) |
| Prevalence | Mali | 31.8 (12.7 to 52) | 0.5 (0.2 to 0.9) | 82.9 (28.1 to 146.6) | 0.5 (0.2 to 0.9) | -0.04 (-0.92 to 0.84) |
| Prevalence | Malta | 16.1 (10.7 to 23.8) | 14.1 (9.4 to 20.8) | 26.7 (16.6 to 41.2) | 31.8 (19.8 to 49.1) | 2.85 (0.98 to 4.75) |
| Prevalence | Marshall Islands | 0.2 (0.1 to 0.3) | 0.7 (0.4 to 1.1) | 0.3 (0.1 to 0.4) | 1.1 (0.6 to 1.8) | 1.69 (1.37 to 2.01) |
| Prevalence | Mauritania | 9.8 (6.2 to 15.6) | 0.8 (0.5 to 1.2) | 37.7 (16.7 to 60.6) | 1.6 (0.7 to 2.6) | 2.19 (1.67 to 2.72) |
| Prevalence | Mauritius | 7.7 (5.8 to 10.6) | 1.8 (1.3 to 2.4) | 5.8 (4.2 to 7.9) | 1.9 (1.4 to 2.6) | 0.61 (-1.93 to 3.21) |
| Prevalence | Mexico | 1861.5 (1672.3 to 2092) | 4.3 (3.9 to 4.8) | 1956 (1639.1 to 2356.7) | 4.6 (3.8 to 5.6) | 0.13 (-0.26 to 0.54) |
| Prevalence | Micronesia (Federated States of) | 0.5 (0.3 to 0.8) | 0.9 (0.5 to 1.5) | 0.4 (0.2 to 0.7) | 1.1 (0.6 to 1.7) | 0.54 (0.21 to 0.87) |
| Prevalence | Monaco | 1.6 (0.9 to 2.7) | 33.8 (18.5 to 56.5) | 3.6 (1.9 to 5.9) | 54.4 (29.3 to 89.9) | 1.94 (1.48 to 2.41) |
| Prevalence | Mongolia | 27.4 (15.9 to 58) | 2.3 (1.4 to 4.9) | 57.7 (36.2 to 85.9) | 4.3 (2.7 to 6.4) | 1.87 (1.31 to 2.42) |
| Prevalence | Montenegro | 46.4 (30.5 to 69) | 21.9 (14.4 to 32.6) | 29.4 (16 to 50.1) | 19.2 (10.2 to 32.9) | -0.39 (-1.93 to 1.18) |
| Prevalence | Morocco | 358.4 (181.1 to 611.2) | 2.8 (1.4 to 4.8) | 567.9 (245.6 to 983.3) | 4.5 (1.9 to 7.8) | 1.45 (1.23 to 1.67) |
| Prevalence | Mozambique | 248 (146.3 to 424.7) | 2.9 (1.7 to 5) | 562.8 (272.2 to 1016.6) | 3 (1.5 to 5.5) | 0.3 (-0.49 to 1.09) |
| Prevalence | Myanmar | 496.7 (227.7 to 1147.4) | 2.6 (1.2 to 6.1) | 747.4 (436.7 to 1206.7) | 3.6 (2.1 to 5.9) | 1.08 (0.8 to 1.37) |
| Prevalence | Namibia | 11.4 (5.8 to 18) | 1.5 (0.7 to 2.3) | 30 (15.8 to 50.4) | 2.8 (1.5 to 4.8) | 2.1 (1.69 to 2.51) |
| Prevalence | Nauru | 0.1 (0 to 0.1) | 1.2 (0.8 to 1.9) | 0.1 (0 to 0.1) | 1.6 (0.9 to 2.6) | 0.94 (0.74 to 1.15) |
| Prevalence | Nepal | 256.9 (134.5 to 533.1) | 2.4 (1.2 to 4.8) | 360.9 (213.2 to 595.9) | 2.9 (1.7 to 4.9) | 0.78 (0.46 to 1.1) |
| Prevalence | Netherlands | 716.8 (515.9 to 981.5) | 19.4 (14 to 26.5) | 811.4 (545 to 1167.7) | 22.2 (14.9 to 32) | 0.5 (-0.09 to 1.09) |
| Prevalence | New Zealand | 98.3 (72.2 to 134) | 9.1 (6.7 to 12.4) | 121.9 (81.7 to 181.9) | 9.4 (6.3 to 14) | -0.08 (-1.34 to 1.19) |
| Prevalence | Nicaragua | 87.8 (58.4 to 134.3) | 3.8 (2.5 to 5.8) | 111.9 (65.1 to 180.9) | 4.3 (2.5 to 7) | 0.44 (0.33 to 0.56) |
| Prevalence | Niger | 44 (25.6 to 80) | 0.8 (0.5 to 1.4) | 162.6 (65.5 to 271.2) | 1 (0.4 to 1.6) | 0.67 (0.01 to 1.33) |
| Prevalence | Nigeria | 514.7 (297.7 to 995) | 0.9 (0.6 to 1.8) | 1847.8 (753.1 to 2806.1) | 1.4 (0.6 to 2.1) | 1.24 (1.04 to 1.43) |
| Prevalence | Niue | 0 (0 to 0) | 1.5 (0.9 to 2.3) | 0.1 (0 to 0.1) | 11 (6.1 to 18.1) | 7.52 (7.1 to 7.94) |
| Prevalence | North Macedonia | 85.7 (57.9 to 127.3) | 12.6 (8.5 to 18.7) | 71.5 (42.2 to 111.3) | 16.2 (9.4 to 25.3) | 0.78 (0.03 to 1.53) |
| Prevalence | Northern Mariana Islands | 0.3 (0.1 to 0.6) | 1.8 (0.9 to 3.6) | 0.4 (0.2 to 0.7) | 2.6 (1.5 to 4.4) | 0.95 (0.55 to 1.36) |
| Prevalence | Norway | 584.6 (413.8 to 774) | 53.5 (37.9 to 70.9) | 626.1 (499.6 to 752.3) | 49.1 (39 to 59.3) | -0.3 (-1.56 to 0.98) |
| Prevalence | Oman | 34.6 (16.9 to 59.3) | 3.3 (1.6 to 5.6) | 109.9 (53.2 to 192.1) | 7.2 (3.5 to 12.6) | 2.53 (2.11 to 2.95) |
| Prevalence | Pakistan | 1660.2 (919.3 to 2716) | 2.6 (1.5 to 4.3) | 3954.7 (2607.2 to 5893.9) | 3.6 (2.4 to 5.4) | 1.02 (0.76 to 1.27) |
| Prevalence | Palau | 0.1 (0 to 0.1) | 1.2 (0.7 to 1.9) | 0.1 (0 to 0.1) | 1.3 (0.7 to 2.2) | 0.4 (0.16 to 0.64) |
| Prevalence | Palestine | 142.1 (83.8 to 246.5) | 11.4 (6.7 to 19.7) | 476 (275.8 to 825.6) | 19.9 (11.5 to 34.6) | 1.85 (1.3 to 2.4) |
| Prevalence | Panama | 96 (70.7 to 127.7) | 8.9 (6.6 to 11.9) | 105.4 (71.8 to 153.8) | 7.1 (4.8 to 10.3) | -0.9 (-2.17 to 0.39) |
| Prevalence | Papua New Guinea | 16.9 (6.6 to 35.3) | 0.8 (0.3 to 1.6) | 51.9 (25.3 to 92.7) | 1 (0.5 to 1.9) | 0.92 (0.28 to 1.56) |
| Prevalence | Paraguay | 50 (33.4 to 77.8) | 2.3 (1.6 to 3.6) | 120.7 (66.8 to 191.4) | 4.6 (2.5 to 7.3) | 2.39 (2.09 to 2.68) |
| Prevalence | Peru | 772.4 (497.8 to 1242.2) | 7.2 (4.7 to 11.6) | 1291.8 (762.8 to 2124.1) | 10.4 (6.1 to 17.1) | 1.21 (1.05 to 1.36) |
| Prevalence | Philippines | 945.4 (603.5 to 1440.9) | 2.9 (1.9 to 4.5) | 1352.7 (951.9 to 1839.9) | 3 (2.1 to 4.1) | 0.11 (-0.17 to 0.39) |
| Prevalence | Poland | 1067.4 (896.2 to 1283.1) | 8.7 (7.3 to 10.5) | 600.2 (440.3 to 834.9) | 7.8 (5.7 to 10.8) | -0.38 (-0.79 to 0.03) |
| Prevalence | Portugal | 503.6 (366.3 to 681.8) | 17.9 (13.1 to 24.2) | 340.8 (228.7 to 494.1) | 18.2 (12.3 to 26.5) | 0 (-1.08 to 1.1) |
| Prevalence | Puerto Rico | 65 (46.2 to 91.6) | 4.9 (3.5 to 6.9) | 38.8 (25.5 to 57.4) | 6 (4 to 8.7) | 0.6 (-0.14 to 1.35) |
| Prevalence | Qatar | 6.8 (3.9 to 11.6) | 4.4 (2.5 to 7.5) | 59.6 (30.1 to 108.4) | 10 (5.1 to 18.2) | 2.78 (2.03 to 3.53) |
| Prevalence | Republic of Korea | 1762 (1040.2 to 2553.9) | 11.2 (6.6 to 16.3) | 2094.2 (1101.5 to 3315.3) | 26.3 (13.5 to 41.9) | 2.79 (2.35 to 3.24) |
| Prevalence | Republic of Moldova | 174.5 (134.8 to 222.9) | 11 (8.5 to 14) | 75.4 (52.1 to 107.1) | 11.4 (7.8 to 16.2) | -0.03 (-0.89 to 0.83) |
| Prevalence | Romania | 661.9 (535.9 to 808.2) | 9.2 (7.5 to 11.2) | 391 (279.4 to 541) | 9.7 (6.9 to 13.4) | 0.46 (-0.02 to 0.94) |
| Prevalence | Russian Federation | 2953.1 (2825.3 to 3096.4) | 6.6 (6.3 to 6.9) | 2202.9 (1988.1 to 2414.4) | 6.7 (6 to 7.3) | 0.04 (-0.87 to 0.97) |
| Prevalence | Rwanda | 137.2 (82.8 to 216.7) | 3 (1.8 to 4.8) | 217 (122 to 343.2) | 3.4 (1.9 to 5.3) | 0.69 (0.42 to 0.96) |
| Prevalence | Saint Kitts and Nevis | 0.5 (0.4 to 0.7) | 2.9 (2.1 to 3.8) | 0.8 (0.5 to 1.1) | 5.5 (3.8 to 7.8) | 2.38 (1.07 to 3.7) |
| Prevalence | Saint Lucia | 1.9 (1.4 to 2.6) | 2.8 (2.1 to 4) | 2.1 (1.5 to 3) | 5.2 (3.5 to 7.4) | 1.87 (1.26 to 2.49) |
| Prevalence | Saint Vincent and the Grenadines | 1.8 (1.3 to 2.4) | 3.3 (2.4 to 4.5) | 1.8 (1.3 to 2.4) | 5.2 (3.7 to 7.2) | 1.49 (-0.56 to 3.59) |
| Prevalence | Samoa | 2.4 (1.4 to 3.9) | 2.6 (1.5 to 4.3) | 3.2 (1.6 to 5.5) | 3.1 (1.6 to 5.4) | 0.6 (0.16 to 1.04) |
| Prevalence | San Marino | 3.2 (1.9 to 5.2) | 57.6 (33.5 to 93.2) | 2.7 (1.5 to 4.7) | 45.5 (24.1 to 78.6) | -0.79 (-1.26 to -0.31) |
| Prevalence | Sao Tome and Principe | 0.6 (0.4 to 0.9) | 0.8 (0.5 to 1.3) | 0.7 (0.3 to 1.3) | 0.7 (0.3 to 1.3) | -0.58 (-2.25 to 1.11) |
| Prevalence | Saudi Arabia | 431.9 (242.6 to 713.6) | 5.1 (2.9 to 8.5) | 661 (336.9 to 1288.3) | 6.5 (3.3 to 12.7) | 0.78 (0.57 to 0.98) |
| Prevalence | Senegal | 49.8 (32.9 to 75.5) | 1 (0.7 to 1.5) | 131.9 (62.7 to 206) | 1.6 (0.8 to 2.5) | 1.44 (0.58 to 2.32) |
| Prevalence | Serbia | 363.8 (224.1 to 578.2) | 12.9 (7.9 to 20.6) | 198.6 (117.8 to 319) | 10.5 (6.2 to 16.9) | -0.52 (-1.48 to 0.45) |
| Prevalence | Seychelles | 1.3 (0.8 to 1.9) | 4 (2.5 to 6.1) | 1 (0.6 to 1.7) | 3.4 (1.9 to 5.8) | -0.4 (-1.89 to 1.11) |
| Prevalence | Sierra Leone | 21.8 (12.9 to 36) | 0.8 (0.5 to 1.4) | 66.2 (29.7 to 105.4) | 1.4 (0.6 to 2.2) | 1.72 (0.7 to 2.75) |
| Prevalence | Singapore | 70.1 (49.7 to 100) | 8 (5.7 to 11.4) | 220.2 (140.2 to 323.2) | 21.4 (13.6 to 31.5) | 2.53 (0.68 to 4.41) |
| Prevalence | Slovakia | 90.3 (62.6 to 128) | 5.2 (3.6 to 7.4) | 86.6 (52.5 to 133.9) | 7.8 (4.7 to 12) | 1.31 (1.09 to 1.53) |
| Prevalence | Slovenia | 39.4 (29.1 to 52.9) | 7.1 (5.3 to 9.5) | 37.6 (23.9 to 58.2) | 9.2 (5.8 to 14.2) | 0.69 (-0.75 to 2.16) |
| Prevalence | Solomon Islands | 1.3 (0.5 to 2.3) | 0.7 (0.3 to 1.2) | 3.4 (1.5 to 5.4) | 1 (0.5 to 1.6) | 1.31 (0.55 to 2.07) |
| Prevalence | Somalia | 80.1 (39.8 to 154.2) | 1.5 (0.8 to 2.9) | 173.4 (73.8 to 303.2) | 1.3 (0.6 to 2.2) | -0.43 (-0.96 to 0.1) |
| Prevalence | South Africa | 280.2 (205.4 to 433.6) | 1.6 (1.2 to 2.5) | 409.1 (300.7 to 568.5) | 2.1 (1.5 to 2.9) | 0.81 (0.49 to 1.14) |
| Prevalence | South Sudan | 86.9 (50 to 153.5) | 2.4 (1.4 to 4.3) | 148.1 (86.9 to 240.7) | 2.7 (1.6 to 4.3) | 0.33 (-0.35 to 1.02) |
| Prevalence | Spain | 2004.7 (1431.5 to 2822.7) | 18.5 (13.2 to 26) | 2414.8 (1602.3 to 3553.7) | 27.3 (18.1 to 40.3) | 1.11 (0.86 to 1.37) |
| Prevalence | Sri Lanka | 210 (134.9 to 327.1) | 2.9 (1.9 to 4.5) | 358.2 (199.5 to 592.7) | 5.1 (2.8 to 8.4) | 1.98 (1.67 to 2.29) |
| Prevalence | Sudan | 630.2 (239.4 to 1704.6) | 5.4 (2.1 to 14.3) | 2136.4 (1118.2 to 3737.9) | 10 (5.3 to 17.6) | 2.05 (1.91 to 2.19) |
| Prevalence | Suriname | 10.4 (5.7 to 15.7) | 6.1 (3.4 to 9.3) | 15.4 (9.5 to 23.9) | 8.3 (5.1 to 12.9) | 0.95 (0.31 to 1.6) |
| Prevalence | Sweden | 889.2 (644.1 to 1191.7) | 42.5 (30.8 to 56.9) | 866.3 (627.6 to 1129.5) | 35.6 (25.8 to 46.5) | -0.58 (-1.87 to 0.73) |
| Prevalence | Switzerland | 312.1 (218.4 to 439.7) | 19.9 (13.9 to 28) | 399.4 (261.1 to 579.6) | 22.7 (14.8 to 32.9) | 0.46 (-0.46 to 1.38) |
| Prevalence | Syrian Arab Republic | 740.9 (426.5 to 1314) | 9.9 (5.7 to 17.4) | 899.1 (519.2 to 1534.9) | 16.6 (9.4 to 28.8) | 1.73 (1.36 to 2.1) |
| Prevalence | Taiwan (Province of China) | 775.3 (588.3 to 1020.8) | 10.6 (8.1 to 13.9) | 468.4 (306.9 to 702) | 11.5 (7.5 to 17.2) | 0.28 (-0.25 to 0.82) |
| Prevalence | Tajikistan | 282.2 (152.4 to 523.2) | 9.2 (5.1 to 16.9) | 482.4 (285.3 to 783.8) | 10.5 (6.2 to 17) | 0.41 (0.18 to 0.64) |
| Prevalence | Thailand | 1132 (611.5 to 1741.2) | 5 (2.7 to 7.7) | 922.6 (539.9 to 1460.3) | 6.7 (3.9 to 10.6) | 0.91 (0.44 to 1.39) |
| Prevalence | Timor-Leste | 8.6 (3.8 to 23.5) | 2 (0.9 to 5.2) | 17.5 (10.1 to 28.2) | 2.6 (1.5 to 4.1) | 0.71 (0.22 to 1.19) |
| Prevalence | Togo | 19 (12.3 to 28.4) | 0.8 (0.5 to 1.2) | 52.6 (23.7 to 84.9) | 1.2 (0.6 to 2) | 1.37 (0.83 to 1.91) |
| Prevalence | Tokelau | 0 (0 to 0) | 1.2 (0.7 to 1.9) | 0.1 (0 to 0.1) | 14.7 (6.7 to 23.5) | 9.89 (9.42 to 10.35) |
| Prevalence | Tonga | 0.8 (0.4 to 1.4) | 1.4 (0.7 to 2.6) | 1 (0.5 to 1.7) | 1.9 (1 to 3.5) | 0.95 (0.61 to 1.29) |
| Prevalence | Trinidad and Tobago | 14.8 (10.8 to 19.6) | 2.8 (2.1 to 3.8) | 12.9 (9 to 18.2) | 3.6 (2.5 to 5.1) | 0.76 (-0.44 to 1.98) |
| Prevalence | Tunisia | 335.5 (166.6 to 553) | 8.4 (4.2 to 13.9) | 424.5 (204 to 735) | 12 (5.7 to 20.8) | 1.14 (0.86 to 1.42) |
| Prevalence | Turkey | 3228.3 (1822.8 to 5544.4) | 12.3 (6.9 to 21.1) | 5217 (2945.5 to 8431.8) | 21.8 (12.2 to 35.1) | 1.88 (1.64 to 2.12) |
| Prevalence | Turkmenistan | 49.1 (38.1 to 62.3) | 2.5 (1.9 to 3.2) | 206.5 (153 to 279.1) | 10.5 (7.8 to 14.2) | 4.82 (3.96 to 5.69) |
| Prevalence | Tuvalu | 0 (0 to 0.1) | 0.9 (0.5 to 1.8) | 0.1 (0 to 0.1) | 1.2 (0.6 to 1.8) | 0.84 (0.57 to 1.11) |
| Prevalence | Uganda | 348.4 (187.5 to 560.8) | 2.9 (1.6 to 4.7) | 1042.1 (529.9 to 1749) | 4 (2.1 to 6.7) | 1.03 (0.2 to 1.88) |
| Prevalence | Ukraine | 971.8 (655 to 1372.9) | 6.5 (4.4 to 9.2) | 652.3 (458.5 to 894.6) | 8.1 (5.7 to 11.2) | 0.73 (0.11 to 1.36) |
| Prevalence | United Arab Emirates | 34.3 (16.7 to 73.9) | 4.9 (2.4 to 10.4) | 115.8 (61.3 to 216.4) | 6.9 (3.6 to 12.9) | 1.04 (0.36 to 1.71) |
| Prevalence | United Kingdom | 1620.3 (1468 to 1793.1) | 11.1 (10 to 12.2) | 1605.8 (1415.9 to 1830) | 10.2 (9 to 11.7) | -0.02 (-0.52 to 0.48) |
| Prevalence | United Republic of Tanzania | 553.9 (326.7 to 816.1) | 3.4 (2 to 5) | 1318.9 (764.2 to 2102.1) | 4.2 (2.4 to 6.6) | 0.67 (0.48 to 0.85) |
| Prevalence | United States of America | 14532.5 (13503.8 to 15685.5) | 19.8 (18.4 to 21.3) | 16247.7 (14582 to 18110.6) | 20.1 (18 to 22.5) | 0.09 (-0.33 to 0.51) |
| Prevalence | United States Virgin Islands | 1.5 (0.9 to 2.6) | 3.5 (2 to 6.1) | 0.6 (0.3 to 1.2) | 3.5 (1.6 to 6.6) | -0.01 (-0.75 to 0.73) |
| Prevalence | Uruguay | 68.7 (50.2 to 95.3) | 6.5 (4.7 to 8.9) | 70.5 (46.5 to 105.5) | 7.8 (5.1 to 11.7) | 0.41 (-0.17 to 0.98) |
| Prevalence | Uzbekistan | 643.7 (420.9 to 919.6) | 5.8 (3.8 to 8.3) | 1198.8 (882.1 to 1603.4) | 9.3 (6.8 to 12.4) | 1.5 (1.17 to 1.84) |
| Prevalence | Vanuatu | 0.5 (0.2 to 0.9) | 0.6 (0.3 to 1) | 1.2 (0.7 to 2) | 0.8 (0.4 to 1.3) | 1.03 (-0.67 to 2.76) |
| Prevalence | Venezuela (Bolivarian Republic of) | 155.8 (125.9 to 196.2) | 1.7 (1.4 to 2.2) | 398.8 (264.9 to 578.3) | 4.5 (3 to 6.6) | 3.28 (2.24 to 4.34) |
| Prevalence | Viet Nam | 702.6 (373.4 to 1119.9) | 2.1 (1.1 to 3.3) | 1148 (615.9 to 1890.2) | 3.6 (1.9 to 6) | 1.82 (1.51 to 2.12) |
| Prevalence | Yemen | 311.9 (125.1 to 793.3) | 3.4 (1.3 to 8.4) | 1041.3 (536.5 to 1746.4) | 5.9 (3 to 9.9) | 1.72 (1.29 to 2.16) |
| Prevalence | Zambia | 164.6 (99.3 to 255.1) | 3.2 (1.9 to 4.9) | 377.8 (204.4 to 593.8) | 3.6 (1.9 to 5.6) | 0.44 (0.14 to 0.74) |
| Prevalence | Zimbabwe | 110.6 (62.2 to 167.1) | 1.8 (1 to 2.7) | 246.7 (144.6 to 377.6) | 3.1 (1.8 to 4.7) | 1.41 (0.13 to 2.7) |
| Incidence | Afghanistan | 64.7 (26.1 to 208.5) | 1.2 (0.5 to 3.6) | 283.7 (145.5 to 504) | 1.6 (0.8 to 2.8) | 1.03 (0.38 to 1.69) |
| Incidence | Albania | 48.8 (31.1 to 68.8) | 3.4 (2.2 to 4.7) | 24.7 (13.4 to 40) | 4.2 (2.3 to 6.8) | 0.54 (-0.15 to 1.23) |
| Incidence | Algeria | 211.9 (106 to 329.6) | 1.5 (0.8 to 2.4) | 273.8 (131.6 to 442.3) | 1.6 (0.8 to 2.6) | 0.17 (0.05 to 0.28) |
| Incidence | American Samoa | 0.1 (0.1 to 0.2) | 0.4 (0.3 to 0.7) | 0.1 (0.1 to 0.2) | 0.6 (0.4 to 1) | 1.26 (0.64 to 1.89) |
| Incidence | Andorra | 0.8 (0.4 to 1.5) | 6.3 (3.1 to 12.2) | 0.6 (0.3 to 1.1) | 4.5 (2.4 to 7.7) | -1.02 (-1.52 to -0.51) |
| Incidence | Angola | 20.7 (8.9 to 53) | 0.3 (0.2 to 0.8) | 67.3 (37.8 to 111.5) | 0.4 (0.2 to 0.6) | 0.16 (-0.25 to 0.58) |
| Incidence | Antigua and Barbuda | 0.2 (0.2 to 0.3) | 1 (0.7 to 1.4) | 0.4 (0.3 to 0.5) | 1.7 (1.2 to 2.3) | 1.58 (1.07 to 2.09) |
| Incidence | Argentina | 182.6 (137.2 to 245.5) | 1.4 (1.1 to 1.9) | 228.2 (159.9 to 324.9) | 1.7 (1.2 to 2.4) | 0.78 (0.51 to 1.04) |
| Incidence | Armenia | 33.2 (22.8 to 46.2) | 2.5 (1.7 to 3.4) | 20.1 (14.6 to 28.1) | 2.6 (1.9 to 3.7) | 0.08 (-0.93 to 1.11) |
| Incidence | Australia | 124.7 (99.7 to 156.5) | 2.4 (2 to 3.1) | 130.5 (90.7 to 181.3) | 2.1 (1.5 to 2.9) | -0.43 (-1.01 to 0.15) |
| Incidence | Austria | 67.3 (47 to 95.4) | 3.7 (2.6 to 5.2) | 64.5 (42.9 to 90) | 3.7 (2.4 to 5.1) | -0.01 (-0.74 to 0.73) |
| Incidence | Azerbaijan | 88.5 (53.5 to 139.6) | 2.8 (1.7 to 4.4) | 101.5 (60.7 to 159.2) | 3.3 (2 to 5.2) | 0.6 (0.28 to 0.91) |
| Incidence | Bahamas | 0.9 (0.6 to 1.2) | 0.8 (0.6 to 1.1) | 1.3 (0.9 to 1.7) | 1.1 (0.7 to 1.5) | 1.15 (-0.3 to 2.61) |
| Incidence | Bahrain | 2.2 (1.4 to 3.4) | 1.1 (0.7 to 1.7) | 4.7 (2.7 to 7.9) | 1.1 (0.7 to 1.9) | 0.19 (-0.37 to 0.74) |
| Incidence | Bangladesh | 666.3 (350.1 to 1407.9) | 1.1 (0.6 to 2.2) | 801.9 (496.2 to 1217.9) | 1.3 (0.8 to 2) | 0.69 (0.4 to 0.98) |
| Incidence | Barbados | 1.4 (1 to 1.8) | 1.6 (1.2 to 2.2) | 1 (0.7 to 1.5) | 1.6 (1 to 2.3) | -0.26 (-1.3 to 0.79) |
| Incidence | Belarus | 36.8 (27.3 to 48.5) | 1.2 (0.9 to 1.5) | 41.5 (27 to 60.5) | 2 (1.3 to 3) | 1.74 (-0.18 to 3.71) |
| Incidence | Belgium | 88 (63.1 to 122.3) | 3.6 (2.6 to 5) | 100.2 (66.9 to 146.6) | 3.9 (2.6 to 5.8) | -0.12 (-1.82 to 1.61) |
| Incidence | Belize | 1.2 (0.9 to 1.6) | 1.1 (0.8 to 1.5) | 1.7 (1.3 to 2.3) | 1 (0.8 to 1.4) | -0.35 (-0.93 to 0.23) |
| Incidence | Benin | 9.7 (6.1 to 14.6) | 0.3 (0.2 to 0.4) | 39.3 (17.1 to 65) | 0.5 (0.2 to 0.8) | 1.71 (1.2 to 2.22) |
| Incidence | Bermuda | 0.3 (0.2 to 0.4) | 1.6 (1 to 2.4) | 0.3 (0.2 to 0.5) | 3.1 (1.9 to 4.8) | 2.16 (1.77 to 2.54) |
| Incidence | Bhutan | 2.7 (0.8 to 5.1) | 0.8 (0.2 to 1.5) | 2.9 (1.7 to 4.9) | 1.2 (0.7 to 2) | 1.53 (0.87 to 2.19) |
| Incidence | Bolivia (Plurinational State of) | 50.9 (29.7 to 90.7) | 1.5 (0.9 to 2.6) | 92.6 (55.8 to 141) | 2.1 (1.2 to 3.1) | 1.07 (1 to 1.14) |
| Incidence | Bosnia and Herzegovina | 33.2 (23.4 to 48.8) | 2.3 (1.6 to 3.3) | 17.1 (10.9 to 25.3) | 2.5 (1.6 to 3.7) | 0.29 (-0.36 to 0.94) |
| Incidence | Botswana | 3.1 (1.9 to 5.1) | 0.4 (0.3 to 0.7) | 6.1 (3.7 to 9.7) | 0.7 (0.4 to 1.1) | 1.49 (1.08 to 1.9) |
| Incidence | Brazil | 1215.5 (1031.2 to 1405.3) | 1.8 (1.6 to 2.1) | 1164.1 (962.6 to 1363.4) | 1.8 (1.5 to 2.1) | -0.07 (-0.45 to 0.31) |
| Incidence | Brunei Darussalam | 2.2 (1.4 to 3.6) | 2 (1.2 to 3.2) | 2.6 (1.6 to 4) | 2.1 (1.3 to 3.2) | 0.15 (-0.18 to 0.48) |
| Incidence | Bulgaria | 61.7 (50.6 to 74.3) | 2.7 (2.2 to 3.2) | 27.7 (21 to 36.1) | 2.1 (1.6 to 2.8) | -0.85 (-3.07 to 1.41) |
| Incidence | Burkina Faso | 15.9 (9.6 to 25.7) | 0.2 (0.2 to 0.4) | 60.6 (27.2 to 95.1) | 0.4 (0.2 to 0.7) | 1.88 (1.57 to 2.19) |
| Incidence | Burundi | 31.3 (17.3 to 53) | 0.9 (0.5 to 1.5) | 58.2 (27.4 to 99.8) | 0.8 (0.4 to 1.3) | -0.49 (-0.85 to -0.14) |
| Incidence | Cabo Verde | 1.3 (0.7 to 2.6) | 0.6 (0.4 to 1.3) | 2.7 (1.5 to 4.5) | 1.4 (0.8 to 2.4) | 2.71 (2.43 to 2.98) |
| Incidence | Cambodia | 46.5 (23.2 to 94.1) | 0.8 (0.4 to 1.6) | 65.5 (39.2 to 101.4) | 1 (0.6 to 1.5) | 0.71 (0.53 to 0.89) |
| Incidence | Cameroon | 18.8 (11.8 to 28.2) | 0.3 (0.2 to 0.4) | 78.4 (35 to 124.5) | 0.5 (0.2 to 0.7) | 1.51 (0.89 to 2.14) |
| Incidence | Canada | 261.9 (186.6 to 361.9) | 3.4 (2.5 to 4.8) | 255.8 (171.5 to 369.4) | 3.1 (2.1 to 4.4) | -0.35 (-1.19 to 0.49) |
| Incidence | Central African Republic | 4.8 (2.4 to 11.1) | 0.3 (0.2 to 0.7) | 9 (4.7 to 18.1) | 0.3 (0.2 to 0.6) | -0.1 (-0.33 to 0.13) |
| Incidence | Chad | 8.3 (4.9 to 14.5) | 0.2 (0.1 to 0.4) | 44.9 (23.8 to 70.8) | 0.4 (0.2 to 0.6) | 2.03 (1.78 to 2.29) |
| Incidence | Chile | 29.1 (22.6 to 37.9) | 0.6 (0.4 to 0.7) | 52.2 (36.1 to 78.6) | 1.1 (0.7 to 1.6) | 2.22 (1.06 to 3.4) |
| Incidence | China | 14171.3 (9458.9 to 18317.9) | 3.3 (2.2 to 4.3) | 10465.9 (7722.2 to 14473.9) | 3.1 (2.3 to 4.4) | -0.29 (-0.5 to -0.09) |
| Incidence | Colombia | 209.9 (168.6 to 257.2) | 1.4 (1.1 to 1.7) | 246.7 (176.4 to 339.1) | 1.7 (1.2 to 2.3) | 0.48 (-0.15 to 1.11) |
| Incidence | Comoros | 2.7 (1.3 to 4.1) | 1 (0.5 to 1.5) | 3.7 (2.1 to 5.9) | 1.2 (0.7 to 1.9) | 0.47 (-1.07 to 2.03) |
| Incidence | Congo | 4.5 (2.4 to 8.3) | 0.3 (0.2 to 0.6) | 9 (5.4 to 13.8) | 0.4 (0.2 to 0.6) | 0.36 (-0.18 to 0.9) |
| Incidence | Cook Islands | 0 (0 to 0) | 0.1 (0.1 to 0.2) | 0 (0 to 0) | 0.2 (0.1 to 0.4) | 2.55 (1.41 to 3.71) |
| Incidence | Costa Rica | 17.4 (13.6 to 22.1) | 1.2 (0.9 to 1.5) | 17.5 (12.6 to 24.5) | 1.3 (0.9 to 1.8) | 0.18 (-0.8 to 1.17) |
| Incidence | Coted'Ivoire | 22.1 (10.7 to 33.4) | 0.3 (0.1 to 0.4) | 52.7 (22.5 to 87.6) | 0.3 (0.2 to 0.6) | 0.61 (0.09 to 1.13) |
| Incidence | Croatia | 41.7 (30.6 to 57.1) | 3.2 (2.3 to 4.3) | 27.9 (18.3 to 42.1) | 3.4 (2.3 to 5.2) | 0.14 (-1.49 to 1.79) |
| Incidence | Cuba | 65.5 (49.2 to 89.4) | 1.8 (1.4 to 2.5) | 51 (35.6 to 70.9) | 2.1 (1.5 to 3) | 0.41 (-0.13 to 0.96) |
| Incidence | Cyprus | 4.9 (3.1 to 8.1) | 1.9 (1.2 to 3.1) | 6.6 (3.6 to 11.2) | 2.3 (1.3 to 3.9) | 0.6 (0.2 to 1.01) |
| Incidence | Czechia | 50.7 (41.8 to 61.7) | 1.7 (1.4 to 2.1) | 27.3 (19.5 to 37.9) | 1.2 (0.9 to 1.7) | -1.05 (-1.73 to -0.37) |
| Incidence | Democratic People's Republic of Korea | 173 (95.5 to 274.2) | 2.2 (1.2 to 3.4) | 126.3 (70.6 to 206.2) | 2 (1.1 to 3.2) | -0.29 (-0.42 to -0.17) |
| Incidence | Democratic Republic of the Congo | 66.8 (33.2 to 136.9) | 0.3 (0.1 to 0.6) | 137.2 (79.7 to 217.2) | 0.3 (0.2 to 0.5) | -0.1 (-0.33 to 0.14) |
| Incidence | Denmark | 56.3 (39.4 to 79.6) | 4.7 (3.3 to 6.6) | 53.5 (37.1 to 72.1) | 4.2 (2.9 to 5.6) | -0.14 (-0.76 to 0.48) |
| Incidence | Djibouti | 2.5 (1.5 to 3.8) | 1.1 (0.7 to 1.7) | 5.4 (2.9 to 9) | 1 (0.6 to 1.7) | -0.07 (-0.8 to 0.68) |
| Incidence | Dominica | 0.2 (0.1 to 0.3) | 0.6 (0.4 to 1) | 0.2 (0.1 to 0.4) | 1.3 (0.8 to 2) | 2.33 (1.98 to 2.67) |
| Incidence | Dominican Republic | 59.5 (35.5 to 94) | 1.7 (1 to 2.7) | 68.5 (43.1 to 101.4) | 1.8 (1.1 to 2.6) | 0.22 (-0.01 to 0.45) |
| Incidence | Ecuador | 14.7 (11.2 to 18.9) | 0.3 (0.2 to 0.4) | 107.4 (77.5 to 149) | 1.6 (1.2 to 2.3) | 5.85 (3.45 to 8.3) |
| Incidence | Egypt | 441.6 (279 to 906.4) | 1.6 (1 to 3.2) | 1324.3 (849.4 to 1950.4) | 2.8 (1.8 to 4.2) | 2.01 (1.69 to 2.34) |
| Incidence | El Salvador | 35.5 (25.1 to 50.8) | 1.3 (0.9 to 1.8) | 37.5 (23.6 to 57.9) | 1.6 (1 to 2.4) | 0.57 (0.06 to 1.07) |
| Incidence | Equatorial Guinea | 0.8 (0.4 to 1.6) | 0.3 (0.2 to 0.6) | 2.9 (1.3 to 6.1) | 0.4 (0.2 to 0.8) | 0.76 (0.24 to 1.28) |
| Incidence | Eritrea | 16.7 (9.9 to 28.4) | 0.8 (0.5 to 1.3) | 37.1 (21.5 to 59.9) | 1.1 (0.7 to 1.8) | 1.22 (0.94 to 1.5) |
| Incidence | Estonia | 9.4 (7.2 to 12.2) | 2.1 (1.6 to 2.7) | 7.9 (5.4 to 11.4) | 2.8 (1.9 to 4) | 1.16 (-0.96 to 3.32) |
| Incidence | Eswatini | 2.2 (1.3 to 3.7) | 0.5 (0.3 to 0.8) | 3.7 (2.2 to 5.7) | 0.7 (0.4 to 1.1) | 1.41 (1.14 to 1.68) |
| Incidence | Ethiopia | 197.7 (91.7 to 628.4) | 0.6 (0.3 to 1.9) | 439.7 (244.7 to 834.2) | 0.8 (0.4 to 1.4) | 0.64 (0.32 to 0.96) |
| Incidence | Fiji | 2.3 (1 to 3.6) | 0.6 (0.3 to 1) | 2.3 (1.2 to 3.6) | 0.7 (0.3 to 1) | 0.16 (-0.34 to 0.66) |
| Incidence | Finland | 46.4 (31.3 to 66.4) | 3.7 (2.5 to 5.2) | 47.2 (33 to 64.5) | 4 (2.8 to 5.5) | 0.04 (-0.39 to 0.47) |
| Incidence | France | 300.9 (236.1 to 386.7) | 1.9 (1.5 to 2.4) | 359 (256 to 495.1) | 2.3 (1.7 to 3.2) | 0.68 (-0.01 to 1.37) |
| Incidence | Gabon | 1.7 (1 to 2.8) | 0.3 (0.2 to 0.5) | 3.3 (1.9 to 5.5) | 0.4 (0.2 to 0.7) | 0.63 (0.03 to 1.23) |
| Incidence | Gambia | 0.7 (0.3 to 1.1) | 0.1 (0.1 to 0.2) | 1.6 (0.8 to 2.7) | 0.1 (0.1 to 0.2) | 0.5 (-0.89 to 1.91) |
| Incidence | Georgia | 17.9 (11.6 to 26.8) | 1 (0.6 to 1.5) | 18.5 (13.5 to 24.6) | 2 (1.5 to 2.7) | 2.49 (1.21 to 3.78) |
| Incidence | Germany | 401 (325.2 to 502.2) | 2.3 (1.9 to 2.9) | 375.1 (276.4 to 510.5) | 2.4 (1.7 to 3.2) | -0.13 (-0.63 to 0.38) |
| Incidence | Ghana | 100.8 (60 to 151.9) | 1.2 (0.7 to 1.8) | 159.3 (86.2 to 262.3) | 1 (0.5 to 1.6) | -0.6 (-0.99 to -0.21) |
| Incidence | Greece | 131.1 (92.5 to 183.4) | 4.7 (3.3 to 6.6) | 78.9 (55.8 to 114.2) | 4.2 (2.9 to 6) | -0.26 (-2.17 to 1.68) |
| Incidence | Greenland | 0.6 (0.4 to 1) | 3.6 (1.9 to 5.5) | 0.3 (0.2 to 0.5) | 1.9 (1.2 to 3) | -2.09 (-2.42 to -1.75) |
| Incidence | Grenada | 0.4 (0.3 to 0.6) | 1 (0.6 to 1.4) | 0.4 (0.3 to 0.5) | 1.3 (1 to 1.8) | 1.22 (0.72 to 1.71) |
| Incidence | Guam | 0.2 (0.1 to 0.3) | 0.4 (0.2 to 0.6) | 0.2 (0.1 to 0.4) | 0.5 (0.2 to 0.8) | 0.58 (-1.12 to 2.32) |
| Incidence | Guatemala | 47.4 (40.1 to 55.9) | 0.9 (0.8 to 1.1) | 51.3 (39.3 to 66.5) | 0.8 (0.6 to 1) | -0.54 (-1.26 to 0.18) |
| Incidence | Guinea | 19.2 (10.3 to 29.2) | 0.5 (0.3 to 0.7) | 36.7 (14.5 to 63.8) | 0.5 (0.2 to 0.8) | -0.15 (-0.63 to 0.34) |
| Incidence | Guinea-Bissau | 1.8 (1.1 to 3) | 0.3 (0.2 to 0.5) | 4.4 (2.1 to 7.2) | 0.4 (0.2 to 0.6) | 1.11 (-0.22 to 2.46) |
| Incidence | Guyana | 1.5 (1.1 to 1.9) | 0.4 (0.3 to 0.5) | 2 (1.5 to 2.7) | 0.7 (0.5 to 1) | 2.28 (-0.02 to 4.64) |
| Incidence | Haiti | 46.6 (20.3 to 169.9) | 1.3 (0.6 to 4.7) | 80.9 (40.3 to 185.3) | 1.4 (0.7 to 3.3) | 0.31 (0.02 to 0.61) |
| Incidence | Honduras | 40.5 (27.2 to 56.4) | 1.5 (1 to 2) | 47.4 (23.8 to 80.7) | 1.1 (0.6 to 1.9) | -0.9 (-1 to -0.8) |
| Incidence | Hungary | 53.3 (43.9 to 65.1) | 1.9 (1.5 to 2.3) | 24.2 (17.5 to 32.5) | 1.3 (0.9 to 1.7) | -1.24 (-2.15 to -0.32) |
| Incidence | Iceland | 5.2 (3.6 to 7.5) | 6.2 (4.3 to 8.9) | 4.2 (2.5 to 6.5) | 4.6 (2.8 to 7) | -0.62 (-1.78 to 0.55) |
| Incidence | India | 3380.7 (2036.9 to 5152.5) | 0.8 (0.5 to 1.2) | 3865.5 (2829.3 to 5318.4) | 0.8 (0.6 to 1.1) | -0.08 (-0.37 to 0.21) |
| Incidence | Indonesia | 721.9 (403.8 to 1146.1) | 0.8 (0.5 to 1.3) | 913.1 (593 to 1284.1) | 1 (0.7 to 1.4) | 0.68 (0.45 to 0.9) |
| Incidence | Iran (Islamic Republic of) | 852.6 (496.7 to 1303.4) | 2.6 (1.5 to 4) | 1068.8 (563.3 to 1678.5) | 4.2 (2.2 to 6.6) | 1.45 (0.99 to 1.91) |
| Incidence | Iraq | 202.5 (120.7 to 375.5) | 1.9 (1.2 to 3.6) | 554.4 (332.2 to 902.8) | 3.2 (1.9 to 5.2) | 1.58 (1.26 to 1.89) |
| Incidence | Ireland | 42.6 (30.8 to 59.6) | 3.2 (2.3 to 4.5) | 36.4 (23.7 to 53.7) | 2.8 (1.8 to 4.1) | -0.52 (-1.3 to 0.26) |
| Incidence | Israel | 58.5 (42.7 to 81.1) | 3 (2.2 to 4.1) | 83.1 (55.7 to 122.1) | 2.5 (1.6 to 3.6) | -0.45 (-1.56 to 0.67) |
| Incidence | Italy | 434.2 (329.5 to 576.6) | 3.3 (2.5 to 4.4) | 270.8 (190.6 to 380.4) | 2.6 (1.8 to 3.7) | -0.93 (-1.6 to -0.26) |
| Incidence | Jamaica | 10.6 (7.2 to 15) | 1 (0.7 to 1.4) | 7.9 (5.4 to 11.3) | 1 (0.7 to 1.4) | 0.2 (-0.7 to 1.1) |
| Incidence | Japan | 645.8 (531.6 to 793.3) | 2 (1.7 to 2.5) | 625.6 (446.8 to 821.2) | 3 (2.1 to 3.9) | 1.23 (0.2 to 2.27) |
| Incidence | Jordan | 36.8 (23.7 to 54.8) | 1.7 (1.1 to 2.6) | 110.8 (65.3 to 181.1) | 2.3 (1.4 to 3.8) | 0.95 (0.72 to 1.18) |
| Incidence | Kazakhstan | 88.8 (66.8 to 115.7) | 1.3 (1 to 1.7) | 84.3 (62 to 111.9) | 1.2 (0.9 to 1.6) | -0.52 (-1.33 to 0.3) |
| Incidence | Kenya | 87.4 (57.2 to 152.5) | 0.6 (0.4 to 1) | 188.3 (127.9 to 297.9) | 0.8 (0.5 to 1.2) | 0.79 (0.3 to 1.28) |
| Incidence | Kiribati | 0.1 (0 to 0.1) | 0.2 (0.1 to 0.4) | 0.1 (0.1 to 0.2) | 0.3 (0.1 to 0.5) | 0.14 (-0.27 to 0.56) |
| Incidence | Kuwait | 15.1 (10.7 to 21) | 2.1 (1.5 to 3) | 17.3 (10.9 to 26.1) | 1.6 (1 to 2.4) | -0.72 (-2.22 to 0.81) |
| Incidence | Kyrgyzstan | 12.8 (9.4 to 17.7) | 0.6 (0.4 to 0.8) | 37 (27 to 48.8) | 1.3 (1 to 1.7) | 2.65 (1.94 to 3.37) |
| Incidence | Lao People's Democratic Republic | 16.6 (7.9 to 42.7) | 0.7 (0.4 to 1.8) | 26.6 (15.4 to 42.5) | 0.9 (0.5 to 1.4) | 0.72 (0.47 to 0.97) |
| Incidence | Latvia | 13.8 (10.6 to 18.3) | 1.8 (1.4 to 2.4) | 7.5 (5.1 to 10.8) | 1.9 (1.3 to 2.8) | 0.15 (-1.41 to 1.74) |
| Incidence | Lebanon | 18.4 (10.7 to 29.8) | 1.4 (0.8 to 2.2) | 39.7 (19.7 to 71.2) | 2.4 (1.2 to 4.4) | 1.95 (1.6 to 2.31) |
| Incidence | Lesotho | 2.9 (1.8 to 4.8) | 0.3 (0.2 to 0.6) | 4.8 (2.9 to 7.7) | 0.6 (0.3 to 0.9) | 1.65 (1.23 to 2.07) |
| Incidence | Liberia | 4 (2.2 to 6.9) | 0.3 (0.1 to 0.4) | 10.9 (4.7 to 17.4) | 0.4 (0.2 to 0.6) | 1.19 (0.18 to 2.2) |
| Incidence | Libya | 55.2 (30.3 to 85.3) | 2.4 (1.3 to 3.7) | 65.7 (34.5 to 110.1) | 3.3 (1.7 to 5.6) | 1.05 (0.49 to 1.61) |
| Incidence | Lithuania | 16.8 (13.5 to 21) | 1.5 (1.2 to 1.9) | 9.7 (7.2 to 12.9) | 1.8 (1.3 to 2.4) | 0.79 (-0.5 to 2.1) |
| Incidence | Luxembourg | 3.5 (2.5 to 4.9) | 4 (2.9 to 5.6) | 4 (2.5 to 6.1) | 3 (1.9 to 4.5) | -0.95 (-1.82 to -0.07) |
| Incidence | Madagascar | 61.7 (40.1 to 95.1) | 0.8 (0.5 to 1.3) | 123.4 (70.9 to 196.7) | 0.8 (0.5 to 1.3) | -0.07 (-0.51 to 0.37) |
| Incidence | Malawi | 28.2 (15.1 to 44.4) | 0.4 (0.2 to 0.7) | 46.3 (20.8 to 78.9) | 0.4 (0.2 to 0.8) | 0.06 (-0.46 to 0.57) |
| Incidence | Malaysia | 61.4 (29.6 to 98.6) | 0.7 (0.4 to 1.2) | 87.7 (44 to 137.2) | 0.8 (0.4 to 1.3) | 0.55 (-0.09 to 1.2) |
| Incidence | Maldives | 1 (0.5 to 2.4) | 0.8 (0.4 to 1.8) | 1.5 (0.9 to 2.3) | 1.1 (0.7 to 1.8) | 1.07 (0.79 to 1.35) |
| Incidence | Mali | 10.5 (4.3 to 17.1) | 0.2 (0.1 to 0.3) | 26.3 (9.2 to 46.9) | 0.2 (0.1 to 0.3) | -0.17 (-1.02 to 0.69) |
| Incidence | Malta | 2.8 (1.9 to 3.9) | 2.4 (1.6 to 3.5) | 3.9 (2.4 to 5.9) | 4.6 (2.9 to 7) | 2.34 (0.43 to 4.29) |
| Incidence | Marshall Islands | 0.1 (0 to 0.1) | 0.2 (0.1 to 0.4) | 0.1 (0 to 0.1) | 0.4 (0.2 to 0.6) | 1.58 (1.28 to 1.88) |
| Incidence | Mauritania | 3.2 (2 to 5.1) | 0.3 (0.2 to 0.4) | 10.7 (5 to 17.4) | 0.5 (0.2 to 0.7) | 1.68 (1.21 to 2.15) |
| Incidence | Mauritius | 2 (1.5 to 2.5) | 0.5 (0.4 to 0.6) | 1.3 (1 to 1.8) | 0.4 (0.3 to 0.6) | 0.33 (-1.52 to 2.21) |
| Incidence | Mexico | 564.3 (510.2 to 627.8) | 1.3 (1.2 to 1.5) | 505.2 (426.2 to 600.9) | 1.2 (1 to 1.4) | -0.43 (-0.8 to -0.05) |
| Incidence | Micronesia (Federated States of) | 0.2 (0.1 to 0.3) | 0.3 (0.2 to 0.5) | 0.1 (0.1 to 0.2) | 0.3 (0.2 to 0.5) | 0.31 (0 to 0.62) |
| Incidence | Monaco | 0.2 (0.1 to 0.4) | 5 (2.8 to 8.3) | 0.5 (0.3 to 0.8) | 7.6 (4.1 to 12.5) | 1.7 (1.27 to 2.14) |
| Incidence | Mongolia | 9.5 (5.6 to 19.8) | 0.8 (0.5 to 1.7) | 17.3 (10.8 to 25.3) | 1.3 (0.8 to 1.9) | 1.46 (0.86 to 2.07) |
| Incidence | Montenegro | 10.1 (6.9 to 14.8) | 4.8 (3.2 to 7) | 5.5 (3 to 9.2) | 3.6 (1.9 to 6) | -0.87 (-2.44 to 0.74) |
| Incidence | Morocco | 102.2 (53 to 169.5) | 0.8 (0.4 to 1.3) | 123.8 (55.7 to 210.5) | 1 (0.4 to 1.7) | 0.55 (0.27 to 0.83) |
| Incidence | Mozambique | 81.8 (48 to 142.2) | 1 (0.6 to 1.7) | 180.4 (86.6 to 325.4) | 1 (0.5 to 1.8) | 0.2 (-0.58 to 0.99) |
| Incidence | Myanmar | 174.9 (80.7 to 395.8) | 0.9 (0.4 to 2.1) | 228 (135.8 to 358.3) | 1.1 (0.7 to 1.7) | 0.58 (0.32 to 0.83) |
| Incidence | Namibia | 3.8 (1.9 to 5.9) | 0.5 (0.2 to 0.8) | 9 (4.9 to 14.8) | 0.8 (0.5 to 1.4) | 1.71 (1.31 to 2.11) |
| Incidence | Nauru | 0 (0 to 0) | 0.4 (0.3 to 0.6) | 0 (0 to 0) | 0.5 (0.3 to 0.8) | 0.79 (0.61 to 0.97) |
| Incidence | Nepal | 88.3 (46.6 to 182.5) | 0.8 (0.4 to 1.7) | 109.2 (65.2 to 182) | 0.9 (0.5 to 1.5) | 0.3 (-0.02 to 0.63) |
| Incidence | Netherlands | 114 (84.1 to 153.7) | 3.1 (2.3 to 4.1) | 111.3 (74.9 to 158.5) | 3.1 (2.1 to 4.3) | 0.08 (-0.17 to 0.34) |
| Incidence | New Zealand | 26.9 (20.8 to 35.2) | 2.5 (1.9 to 3.3) | 27.7 (19.6 to 39.3) | 2.1 (1.5 to 3) | -0.62 (-2.22 to 1) |
| Incidence | Nicaragua | 26.2 (18 to 39.5) | 1.1 (0.8 to 1.7) | 27.7 (16.8 to 43.9) | 1.1 (0.7 to 1.7) | -0.19 (-0.31 to -0.07) |
| Incidence | Niger | 14.8 (8.7 to 26.6) | 0.3 (0.2 to 0.5) | 53.7 (22.1 to 90.7) | 0.3 (0.1 to 0.5) | 0.61 (-0.04 to 1.27) |
| Incidence | Nigeria | 174.4 (102 to 339.1) | 0.3 (0.2 to 0.6) | 602.7 (252.3 to 907.7) | 0.5 (0.2 to 0.7) | 1.07 (0.79 to 1.35) |
| Incidence | Niue | 0 (0 to 0) | 0.5 (0.3 to 0.7) | 0 (0 to 0) | 2.8 (1.6 to 4.5) | 6.88 (6.33 to 7.43) |
| Incidence | North Macedonia | 24.9 (17.2 to 36.8) | 3.7 (2.5 to 5.4) | 15.5 (9.7 to 23.7) | 3.5 (2.2 to 5.4) | -0.3 (-1.08 to 0.5) |
| Incidence | Northern Mariana Islands | 0.1 (0 to 0.1) | 0.4 (0.2 to 0.9) | 0.1 (0.1 to 0.2) | 0.6 (0.4 to 1) | 1.15 (0.78 to 1.53) |
| Incidence | Norway | 79.2 (57.3 to 103.8) | 7.3 (5.3 to 9.5) | 77.5 (61.9 to 93) | 6.1 (4.8 to 7.3) | -0.59 (-1.87 to 0.71) |
| Incidence | Oman | 8 (4.1 to 13.1) | 0.8 (0.4 to 1.3) | 17.5 (8.8 to 29.4) | 1.2 (0.6 to 1.9) | 1.27 (0.71 to 1.83) |
| Incidence | Pakistan | 588.4 (327.3 to 954.9) | 0.9 (0.5 to 1.5) | 1365.4 (908.2 to 2046.2) | 1.2 (0.8 to 1.9) | 0.91 (0.65 to 1.16) |
| Incidence | Palau | 0 (0 to 0) | 0.3 (0.2 to 0.5) | 0 (0 to 0) | 0.3 (0.2 to 0.5) | 0.09 (-0.07 to 0.26) |
| Incidence | Palestine | 34.4 (20.8 to 59.3) | 2.8 (1.7 to 4.8) | 84.8 (51.5 to 140.6) | 3.5 (2.1 to 5.9) | 0.81 (0.33 to 1.29) |
| Incidence | Panama | 25.9 (19.7 to 33.5) | 2.4 (1.8 to 3.1) | 23.4 (16.6 to 33) | 1.6 (1.1 to 2.2) | -1.5 (-2.9 to -0.08) |
| Incidence | Papua New Guinea | 5.8 (2.3 to 12.3) | 0.3 (0.1 to 0.6) | 17.6 (8.5 to 31.5) | 0.4 (0.2 to 0.6) | 0.86 (0.22 to 1.5) |
| Incidence | Paraguay | 14.5 (9.8 to 22.3) | 0.7 (0.5 to 1.1) | 31.2 (17.9 to 48.8) | 1.2 (0.7 to 1.9) | 2.04 (1.79 to 2.29) |
| Incidence | Peru | 244.7 (159 to 382.2) | 2.3 (1.5 to 3.6) | 299 (178.4 to 471) | 2.4 (1.4 to 3.8) | 0.18 (-0.08 to 0.45) |
| Incidence | Philippines | 320.5 (203.4 to 487) | 1 (0.6 to 1.5) | 439.8 (312.7 to 589.3) | 1 (0.7 to 1.3) | -0.04 (-0.36 to 0.28) |
| Incidence | Poland | 323.2 (280.7 to 377.7) | 2.6 (2.3 to 3.1) | 137.7 (105.7 to 185.3) | 1.8 (1.4 to 2.4) | -1.32 (-1.76 to -0.88) |
| Incidence | Portugal | 103.4 (79.5 to 135.4) | 3.6 (2.8 to 4.8) | 52.1 (35.9 to 74) | 2.8 (1.9 to 4) | -0.95 (-1.98 to 0.08) |
| Incidence | Puerto Rico | 15 (10.9 to 20.4) | 1.1 (0.8 to 1.5) | 7 (4.7 to 10.1) | 1.1 (0.7 to 1.5) | -0.08 (-1.1 to 0.94) |
| Incidence | Qatar | 1.5 (0.9 to 2.4) | 1 (0.6 to 1.6) | 8.9 (4.7 to 15.8) | 1.5 (0.8 to 2.7) | 1.43 (0.89 to 1.98) |
| Incidence | Republic of Korea | 380.9 (238.4 to 538.3) | 2.4 (1.5 to 3.4) | 283.6 (150.6 to 448.7) | 3.5 (1.8 to 5.6) | 1.26 (0.84 to 1.68) |
| Incidence | Republic of Moldova | 47.4 (37.9 to 59.4) | 3 (2.4 to 3.7) | 16.3 (11.8 to 22.5) | 2.4 (1.7 to 3.4) | -0.82 (-1.61 to -0.03) |
| Incidence | Romania | 195.2 (162.5 to 232.6) | 2.7 (2.3 to 3.2) | 81.1 (60.7 to 109.3) | 2 (1.5 to 2.7) | -0.74 (-1.21 to -0.28) |
| Incidence | Russian Federation | 900.9 (864.6 to 940.8) | 2 (1.9 to 2.1) | 522 (474.6 to 566.8) | 1.6 (1.4 to 1.7) | -1.03 (-2.13 to 0.08) |
| Incidence | Rwanda | 47.8 (29 to 75.5) | 1.1 (0.6 to 1.7) | 69.3 (38.9 to 109.8) | 1.1 (0.6 to 1.7) | 0 (-1.17 to 1.19) |
| Incidence | Saint Kitts and Nevis | 0.2 (0.1 to 0.2) | 0.9 (0.7 to 1.2) | 0.2 (0.1 to 0.3) | 1.4 (1 to 1.9) | 1.64 (0.25 to 3.05) |
| Incidence | Saint Lucia | 0.6 (0.4 to 0.8) | 0.8 (0.6 to 1.1) | 0.5 (0.4 to 0.8) | 1.3 (0.9 to 1.8) | 1.46 (0.85 to 2.06) |
| Incidence | Saint Vincent and the Grenadines | 0.5 (0.4 to 0.7) | 1 (0.7 to 1.3) | 0.5 (0.4 to 0.7) | 1.4 (1.1 to 1.9) | 1.34 (-0.56 to 3.28) |
| Incidence | Samoa | 0.7 (0.4 to 1.2) | 0.8 (0.5 to 1.3) | 0.9 (0.5 to 1.5) | 0.9 (0.5 to 1.4) | 0.3 (-0.06 to 0.65) |
| Incidence | San Marino | 0.5 (0.3 to 0.8) | 8.5 (5 to 13.6) | 0.4 (0.2 to 0.6) | 6.3 (3.4 to 10.7) | -0.92 (-1.43 to -0.41) |
| Incidence | Sao Tome and Principe | 0.2 (0.1 to 0.3) | 0.3 (0.2 to 0.4) | 0.2 (0.1 to 0.4) | 0.2 (0.1 to 0.4) | -0.99 (-2.27 to 0.31) |
| Incidence | Saudi Arabia | 93.9 (55.1 to 151.2) | 1.1 (0.7 to 1.8) | 100.5 (53.3 to 187.8) | 1 (0.5 to 1.9) | -0.4 (-0.52 to -0.29) |
| Incidence | Senegal | 15.8 (10.7 to 23.6) | 0.3 (0.2 to 0.5) | 40.1 (19.2 to 63) | 0.5 (0.2 to 0.8) | 1.28 (0.45 to 2.12) |
| Incidence | Serbia | 97.7 (61 to 150.7) | 3.4 (2.1 to 5.3) | 38.5 (23.6 to 61.1) | 2 (1.2 to 3.2) | -1.66 (-2.58 to -0.73) |
| Incidence | Seychelles | 0.4 (0.2 to 0.5) | 1.1 (0.7 to 1.7) | 0.2 (0.1 to 0.4) | 0.8 (0.4 to 1.3) | -1 (-2.71 to 0.74) |
| Incidence | Sierra Leone | 7.1 (4.2 to 11.5) | 0.3 (0.2 to 0.4) | 21 (9.3 to 33.4) | 0.4 (0.2 to 0.7) | 1.61 (0.63 to 2.6) |
| Incidence | Singapore | 12.2 (8.8 to 16.9) | 1.4 (1 to 1.9) | 29.6 (19.1 to 43.1) | 2.9 (1.9 to 4.2) | 2.05 (-0.04 to 4.18) |
| Incidence | Slovakia | 27.4 (19.5 to 38.7) | 1.6 (1.1 to 2.2) | 19.7 (12.5 to 29.9) | 1.8 (1.1 to 2.7) | 0.37 (0.14 to 0.61) |
| Incidence | Slovenia | 8.9 (6.9 to 11.6) | 1.6 (1.2 to 2.1) | 5.9 (3.8 to 9) | 1.4 (0.9 to 2.2) | -0.51 (-2.02 to 1.02) |
| Incidence | Solomon Islands | 0.4 (0.2 to 0.8) | 0.2 (0.1 to 0.4) | 1.1 (0.5 to 1.8) | 0.3 (0.2 to 0.5) | 1.18 (0.57 to 1.8) |
| Incidence | Somalia | 27.9 (13.9 to 53.9) | 0.5 (0.3 to 1) | 61.3 (26.2 to 108.1) | 0.5 (0.2 to 0.8) | -0.38 (-0.9 to 0.15) |
| Incidence | South Africa | 87.8 (64.2 to 135.8) | 0.5 (0.4 to 0.8) | 120.2 (89 to 164.9) | 0.6 (0.4 to 0.8) | 0.61 (0.24 to 0.98) |
| Incidence | South Sudan | 28.9 (16.6 to 50) | 0.8 (0.5 to 1.4) | 48.9 (28.9 to 78.6) | 0.9 (0.5 to 1.4) | 0.31 (-0.39 to 1.01) |
| Incidence | Spain | 340.3 (251.3 to 464.3) | 3.1 (2.3 to 4.3) | 338 (228.2 to 484.4) | 3.8 (2.6 to 5.5) | 0.47 (0.32 to 0.62) |
| Incidence | Sri Lanka | 60.5 (39.6 to 92.1) | 0.8 (0.5 to 1.3) | 76.7 (44.5 to 125.3) | 1.1 (0.6 to 1.8) | 0.9 (0.56 to 1.24) |
| Incidence | Sudan | 190.4 (75.4 to 502.4) | 1.6 (0.7 to 4.2) | 504.2 (271.1 to 854.6) | 2.4 (1.3 to 4) | 1.18 (0.99 to 1.37) |
| Incidence | Suriname | 3.3 (1.8 to 4.9) | 1.9 (1.1 to 2.9) | 4.5 (2.9 to 6.8) | 2.4 (1.5 to 3.6) | 0.72 (-0.11 to 1.55) |
| Incidence | Sweden | 118.8 (86.1 to 159.7) | 5.7 (4.1 to 7.6) | 108.4 (78.7 to 140.9) | 4.5 (3.2 to 5.8) | -0.75 (-2.3 to 0.82) |
| Incidence | Switzerland | 46.5 (33.1 to 64.1) | 3 (2.1 to 4.1) | 54.2 (35.5 to 79) | 3.1 (2 to 4.5) | 0.14 (-0.81 to 1.1) |
| Incidence | Syrian Arab Republic | 190.9 (115.2 to 326.7) | 2.6 (1.6 to 4.4) | 169.1 (104.8 to 275.3) | 3.1 (1.9 to 5.1) | 0.53 (0.1 to 0.97) |
| Incidence | Taiwan (Province of China) | 142 (112.4 to 181.3) | 1.9 (1.5 to 2.5) | 75.2 (50.6 to 108.3) | 1.8 (1.2 to 2.7) | -0.06 (-1.34 to 1.23) |
| Incidence | Tajikistan | 90.3 (49.9 to 164.5) | 3 (1.7 to 5.4) | 146.9 (88.1 to 234.4) | 3.2 (1.9 to 5.1) | 0.22 (-0.03 to 0.46) |
| Incidence | Thailand | 319.2 (179.5 to 478.9) | 1.4 (0.8 to 2.1) | 195.9 (119.4 to 299.3) | 1.4 (0.9 to 2.2) | -0.01 (-0.46 to 0.43) |
| Incidence | Timor-Leste | 3 (1.3 to 7.6) | 0.7 (0.3 to 1.7) | 5.6 (3.3 to 8.9) | 0.8 (0.5 to 1.3) | 0.45 (-0.03 to 0.94) |
| Incidence | Togo | 6.1 (4 to 9) | 0.3 (0.2 to 0.4) | 16.4 (7.4 to 26.5) | 0.4 (0.2 to 0.6) | 1.24 (0.74 to 1.73) |
| Incidence | Tokelau | 0 (0 to 0) | 0.4 (0.2 to 0.6) | 0 (0 to 0) | 3.9 (1.8 to 6.2) | 9.11 (8.45 to 9.77) |
| Incidence | Tonga | 0.2 (0.1 to 0.4) | 0.5 (0.2 to 0.8) | 0.3 (0.1 to 0.5) | 0.6 (0.3 to 1) | 0.73 (0.42 to 1.04) |
| Incidence | Trinidad and Tobago | 4.4 (3.3 to 5.6) | 0.8 (0.6 to 1.1) | 3.4 (2.4 to 4.6) | 0.9 (0.7 to 1.3) | 0.34 (-0.82 to 1.5) |
| Incidence | Tunisia | 76.4 (39.6 to 121.9) | 1.9 (1 to 3.1) | 71.9 (35.1 to 120.9) | 2 (1 to 3.4) | 0.2 (-0.04 to 0.44) |
| Incidence | Turkey | 854.6 (502.9 to 1406) | 3.2 (1.9 to 5.3) | 851.2 (495.8 to 1336.4) | 3.5 (2 to 5.5) | 0.26 (0.04 to 0.47) |
| Incidence | Turkmenistan | 15.5 (12.1 to 19.2) | 0.8 (0.6 to 1) | 59.4 (44.5 to 77.5) | 3 (2.3 to 4) | 4.48 (3.6 to 5.36) |
| Incidence | Tuvalu | 0 (0 to 0) | 0.3 (0.2 to 0.6) | 0 (0 to 0) | 0.4 (0.2 to 0.6) | 0.5 (0.3 to 0.7) |
| Incidence | Uganda | 113.6 (60.9 to 179.8) | 1 (0.5 to 1.5) | 331.9 (170 to 552.9) | 1.3 (0.7 to 2.1) | 0.94 (0.09 to 1.8) |
| Incidence | Ukraine | 321.7 (219.9 to 449.5) | 2.2 (1.5 to 3) | 197.6 (142.2 to 270.2) | 2.4 (1.7 to 3.3) | 0.4 (-0.26 to 1.07) |
| Incidence | United Arab Emirates | 8.9 (4.6 to 18.1) | 1.3 (0.7 to 2.6) | 22.9 (12.7 to 40.5) | 1.4 (0.8 to 2.4) | 0.15 (-0.77 to 1.09) |
| Incidence | United Kingdom | 325.9 (299.3 to 355.5) | 2.2 (2 to 2.4) | 266.2 (237 to 298.2) | 1.7 (1.5 to 1.9) | -0.69 (-1.2 to -0.18) |
| Incidence | United Republic of Tanzania | 179.5 (107.8 to 267.3) | 1.1 (0.7 to 1.7) | 408.7 (238.1 to 645.3) | 1.3 (0.8 to 2) | 0.45 (0.21 to 0.69) |
| Incidence | United States of America | 2338.9 (2174.4 to 2509) | 3.2 (3 to 3.4) | 2363 (2128.7 to 2619.6) | 2.9 (2.6 to 3.2) | -0.25 (-0.66 to 0.16) |
| Incidence | United States Virgin Islands | 0.4 (0.2 to 0.7) | 0.9 (0.6 to 1.6) | 0.1 (0.1 to 0.3) | 0.8 (0.4 to 1.5) | -0.65 (-1.31 to 0.01) |
| Incidence | Uruguay | 15.9 (12.2 to 21.2) | 1.5 (1.1 to 2) | 13.3 (9.2 to 19.3) | 1.5 (1 to 2.1) | -0.39 (-0.97 to 0.21) |
| Incidence | Uzbekistan | 198.9 (131.9 to 284.7) | 1.8 (1.2 to 2.6) | 337.1 (254.6 to 442) | 2.6 (2 to 3.4) | 1.14 (0.74 to 1.53) |
| Incidence | Vanuatu | 0.2 (0.1 to 0.3) | 0.2 (0.1 to 0.4) | 0.4 (0.2 to 0.7) | 0.3 (0.2 to 0.5) | 1.02 (-0.7 to 2.77) |
| Incidence | Venezuela (Bolivarian Republic of) | 45.4 (38 to 54.9) | 0.5 (0.4 to 0.6) | 99.9 (69 to 139.3) | 1.1 (0.8 to 1.6) | 2.73 (1.93 to 3.53) |
| Incidence | Viet Nam | 208.4 (111.7 to 325.7) | 0.6 (0.3 to 1) | 258.3 (141.2 to 409.7) | 0.8 (0.4 to 1.3) | 0.91 (0.59 to 1.23) |
| Incidence | Yemen | 93.1 (37.8 to 235.3) | 1 (0.4 to 2.5) | 261.9 (136.2 to 427.9) | 1.5 (0.8 to 2.4) | 1.2 (0.94 to 1.47) |
| Incidence | Zambia | 54.8 (32.7 to 84) | 1.1 (0.6 to 1.6) | 118.8 (65 to 186.5) | 1.1 (0.6 to 1.8) | 0.22 (-0.06 to 0.5) |
| Incidence | Zimbabwe | 35.4 (19.6 to 52.3) | 0.6 (0.3 to 0.9) | 83.7 (49.2 to 127.7) | 1 (0.6 to 1.6) | 1.59 (0.31 to 2.89) |
| Deaths | Afghanistan | 54.3 (22.3 to 179.8) | 1 (0.4 to 3.1) | 206.7 (108 to 361.8) | 1.2 (0.6 to 2) | 0.59 (-0.03 to 1.22) |
| Deaths | Albania | 36.3 (23.2 to 50.5) | 2.5 (1.6 to 3.5) | 12 (6.7 to 18.3) | 2 (1.1 to 3.1) | -0.64 (-0.86 to -0.41) |
| Deaths | Algeria | 134 (68.7 to 203.4) | 1 (0.5 to 1.5) | 110.7 (58.2 to 165.9) | 0.7 (0.4 to 1) | -1.25 (-1.36 to -1.14) |
| Deaths | American Samoa | 0.1 (0 to 0.1) | 0.3 (0.2 to 0.5) | 0.1 (0 to 0.1) | 0.4 (0.2 to 0.6) | 1.16 (0.53 to 1.79) |
| Deaths | Andorra | 0.2 (0.1 to 0.4) | 1.9 (1 to 3.3) | 0.1 (0.1 to 0.2) | 1 (0.6 to 1.6) | -2 (-2.42 to -1.58) |
| Deaths | Angola | 18.1 (7.8 to 45.8) | 0.3 (0.1 to 0.7) | 54.1 (30.8 to 89) | 0.3 (0.2 to 0.5) | -0.09 (-0.47 to 0.29) |
| Deaths | Antigua and Barbuda | 0.2 (0.1 to 0.2) | 0.6 (0.5 to 0.8) | 0.2 (0.2 to 0.2) | 0.9 (0.7 to 1.1) | 1.2 (0.69 to 1.7) |
| Deaths | Argentina | 113.2 (91.7 to 138.9) | 0.9 (0.7 to 1.1) | 110.3 (89.5 to 133.6) | 0.8 (0.7 to 1) | -0.27 (-0.77 to 0.23) |
| Deaths | Armenia | 23.3 (16.6 to 31.3) | 1.7 (1.2 to 2.3) | 11.1 (8.6 to 14.3) | 1.5 (1.1 to 1.9) | -0.73 (-1.77 to 0.33) |
| Deaths | Australia | 55.2 (48.2 to 62.7) | 1.1 (0.9 to 1.2) | 42.7 (35 to 51.1) | 0.7 (0.6 to 0.8) | -1.49 (-2.11 to -0.87) |
| Deaths | Austria | 17.5 (14.8 to 20.5) | 1 (0.8 to 1.1) | 10.8 (8.8 to 13.2) | 0.6 (0.5 to 0.8) | -1.34 (-2.35 to -0.32) |
| Deaths | Azerbaijan | 69.3 (42.7 to 107.1) | 2.2 (1.4 to 3.4) | 66.1 (40.2 to 102.9) | 2.2 (1.3 to 3.4) | -0.05 (-0.43 to 0.33) |
| Deaths | Bahamas | 0.6 (0.4 to 0.8) | 0.5 (0.4 to 0.7) | 0.8 (0.6 to 1) | 0.7 (0.5 to 0.9) | 0.81 (-0.59 to 2.23) |
| Deaths | Bahrain | 1.2 (0.8 to 1.8) | 0.6 (0.4 to 0.9) | 1.5 (1 to 2.4) | 0.4 (0.2 to 0.6) | -1.57 (-1.92 to -1.23) |
| Deaths | Bangladesh | 561.3 (296.2 to 1164.1) | 0.9 (0.5 to 1.8) | 543.5 (348.4 to 826.2) | 0.9 (0.6 to 1.4) | -0.01 (-0.31 to 0.28) |
| Deaths | Barbados | 0.8 (0.6 to 1.1) | 1 (0.8 to 1.3) | 0.5 (0.4 to 0.8) | 0.8 (0.6 to 1.1) | -0.79 (-1.88 to 0.32) |
| Deaths | Belarus | 21.8 (17.2 to 26.9) | 0.7 (0.5 to 0.9) | 16.2 (12.1 to 21.2) | 0.8 (0.6 to 1) | 0.38 (-1.32 to 2.11) |
| Deaths | Belgium | 29.9 (25.5 to 34.9) | 1.2 (1.1 to 1.4) | 25.5 (20.7 to 31.2) | 1 (0.8 to 1.2) | -1.06 (-2.96 to 0.88) |
| Deaths | Belize | 0.8 (0.6 to 1.1) | 0.8 (0.6 to 1) | 1.1 (0.9 to 1.4) | 0.7 (0.5 to 0.8) | -0.65 (-1.24 to -0.06) |
| Deaths | Benin | 8.1 (5.1 to 12.3) | 0.2 (0.2 to 0.4) | 30.9 (13.4 to 51.1) | 0.4 (0.2 to 0.6) | 1.51 (0.99 to 2.04) |
| Deaths | Bermuda | 0.1 (0.1 to 0.2) | 0.8 (0.6 to 1.1) | 0.1 (0.1 to 0.1) | 1 (0.7 to 1.3) | 0.48 (0.14 to 0.82) |
| Deaths | Bhutan | 2.2 (0.7 to 4.1) | 0.7 (0.2 to 1.2) | 2 (1.2 to 3.3) | 0.8 (0.5 to 1.3) | 0.98 (0.31 to 1.65) |
| Deaths | Bolivia (Plurinational State of) | 43 (25.2 to 77.6) | 1.2 (0.7 to 2.2) | 66.8 (40.8 to 100.9) | 1.5 (0.9 to 2.2) | 0.55 (0.5 to 0.6) |
| Deaths | Bosnia and Herzegovina | 23.3 (16.7 to 33.5) | 1.6 (1.1 to 2.3) | 8.4 (5.8 to 11.7) | 1.2 (0.8 to 1.7) | -0.91 (-1.33 to -0.49) |
| Deaths | Botswana | 2.5 (1.6 to 4.1) | 0.3 (0.2 to 0.5) | 4.5 (2.8 to 7.1) | 0.5 (0.3 to 0.8) | 1.24 (0.84 to 1.64) |
| Deaths | Brazil | 881.9 (750 to 1015.8) | 1.3 (1.1 to 1.5) | 699 (580.5 to 812.9) | 1.1 (0.9 to 1.3) | -0.66 (-1 to -0.32) |
| Deaths | Brunei Darussalam | 1.3 (0.9 to 2.1) | 1.2 (0.8 to 1.8) | 1.3 (0.8 to 1.8) | 1 (0.7 to 1.4) | -0.59 (-0.96 to -0.23) |
| Deaths | Bulgaria | 46.4 (39.1 to 53.9) | 2 (1.7 to 2.3) | 17 (13.7 to 21) | 1.3 (1 to 1.6) | -1.5 (-3.71 to 0.76) |
| Deaths | Burkina Faso | 13.2 (8 to 21.5) | 0.2 (0.1 to 0.3) | 48.8 (21.9 to 75.5) | 0.4 (0.2 to 0.5) | 1.79 (1.47 to 2.12) |
| Deaths | Burundi | 27.2 (15.1 to 45.5) | 0.8 (0.4 to 1.3) | 48.9 (23.2 to 84.9) | 0.7 (0.3 to 1.1) | -0.61 (-1.07 to -0.15) |
| Deaths | Cabo Verde | 0.9 (0.6 to 1.8) | 0.5 (0.3 to 0.9) | 1.6 (0.9 to 2.6) | 0.9 (0.5 to 1.4) | 2.13 (1.63 to 2.64) |
| Deaths | Cambodia | 39 (19.6 to 79.6) | 0.7 (0.3 to 1.3) | 46.4 (28 to 70.6) | 0.7 (0.4 to 1.1) | 0.16 (0.03 to 0.3) |
| Deaths | Cameroon | 15.3 (9.6 to 22.8) | 0.2 (0.1 to 0.3) | 60.4 (27.7 to 94.7) | 0.3 (0.2 to 0.5) | 1.35 (0.77 to 1.93) |
| Deaths | Canada | 74.2 (66 to 83) | 1 (0.9 to 1.1) | 57.5 (47 to 69.9) | 0.7 (0.6 to 0.8) | -1.17 (-1.78 to -0.54) |
| Deaths | Central African Republic | 4.3 (2.2 to 9.9) | 0.3 (0.1 to 0.6) | 8 (4.2 to 16.1) | 0.3 (0.1 to 0.5) | -0.11 (-0.33 to 0.11) |
| Deaths | Chad | 7.1 (4.2 to 12.1) | 0.2 (0.1 to 0.3) | 37.6 (19.6 to 60) | 0.3 (0.2 to 0.5) | 1.98 (1.73 to 2.23) |
| Deaths | Chile | 21.5 (17.6 to 25.9) | 0.4 (0.3 to 0.5) | 26.6 (21.2 to 33.3) | 0.5 (0.4 to 0.7) | 0.94 (-0.32 to 2.21) |
| Deaths | China | 9937.9 (6618.5 to 12853.4) | 2.3 (1.5 to 3) | 3618.6 (2652.1 to 4956.1) | 1.1 (0.8 to 1.5) | -2.58 (-2.82 to -2.35) |
| Deaths | Colombia | 149.4 (125.5 to 177) | 1 (0.8 to 1.2) | 126.7 (98.6 to 159.7) | 0.9 (0.7 to 1.1) | -0.57 (-1.17 to 0.03) |
| Deaths | Comoros | 2.3 (1.1 to 3.4) | 0.8 (0.4 to 1.2) | 2.9 (1.7 to 4.5) | 0.9 (0.5 to 1.5) | 0.29 (-1.26 to 1.87) |
| Deaths | Congo | 3.9 (2.1 to 7) | 0.3 (0.2 to 0.5) | 7 (4.3 to 10.6) | 0.3 (0.2 to 0.4) | 0.08 (-0.46 to 0.62) |
| Deaths | Cook Islands | 0 (0 to 0) | 0.1 (0 to 0.1) | 0 (0 to 0) | 0.1 (0.1 to 0.2) | 1.34 (0.23 to 2.46) |
| Deaths | Costa Rica | 9.8 (8.4 to 11.5) | 0.7 (0.6 to 0.8) | 8.1 (6.5 to 10.2) | 0.6 (0.5 to 0.7) | -0.43 (-1.37 to 0.52) |
| Deaths | Coted'Ivoire | 18.1 (8.9 to 27.4) | 0.2 (0.1 to 0.4) | 40.2 (16.8 to 65.9) | 0.3 (0.1 to 0.4) | 0.4 (-0.09 to 0.89) |
| Deaths | Croatia | 19.1 (16 to 22.6) | 1.5 (1.2 to 1.7) | 8 (6.3 to 10.3) | 1 (0.8 to 1.3) | -1.34 (-2.81 to 0.15) |
| Deaths | Cuba | 36.1 (29 to 45) | 1 (0.8 to 1.3) | 23.2 (18 to 29.3) | 1 (0.7 to 1.2) | -0.21 (-0.85 to 0.44) |
| Deaths | Cyprus | 2.2 (1.5 to 3.2) | 0.8 (0.6 to 1.2) | 1.6 (1 to 2.5) | 0.6 (0.4 to 0.9) | -1.2 (-1.74 to -0.65) |
| Deaths | Czechia | 35.9 (30.8 to 41.8) | 1.2 (1 to 1.4) | 13.3 (10.4 to 16.9) | 0.6 (0.5 to 0.8) | -2.05 (-2.79 to -1.31) |
| Deaths | Democratic People's Republic of Korea | 116.5 (65.2 to 181.8) | 1.5 (0.8 to 2.3) | 71.5 (41.8 to 112.5) | 1.1 (0.6 to 1.8) | -0.86 (-0.96 to -0.77) |
| Deaths | Democratic Republic of the Congo | 57.1 (29.1 to 116) | 0.3 (0.1 to 0.5) | 112.1 (66.8 to 176.8) | 0.2 (0.1 to 0.4) | -0.24 (-0.48 to -0.01) |
| Deaths | Denmark | 13.2 (11.3 to 15.6) | 1.1 (0.9 to 1.3) | 8 (6.4 to 9.9) | 0.6 (0.5 to 0.8) | -1.68 (-2.09 to -1.27) |
| Deaths | Djibouti | 2.1 (1.3 to 3.1) | 0.9 (0.5 to 1.4) | 4.2 (2.3 to 6.9) | 0.8 (0.4 to 1.3) | -0.21 (-0.94 to 0.52) |
| Deaths | Dominica | 0.1 (0.1 to 0.2) | 0.5 (0.3 to 0.7) | 0.2 (0.1 to 0.2) | 0.9 (0.6 to 1.3) | 2.18 (1.84 to 2.52) |
| Deaths | Dominican Republic | 45.1 (27.5 to 69) | 1.3 (0.8 to 2) | 46 (29.7 to 66.9) | 1.2 (0.8 to 1.7) | -0.18 (-0.39 to 0.04) |
| Deaths | Ecuador | 11.2 (8.8 to 14.1) | 0.2 (0.2 to 0.3) | 64.7 (49.4 to 84.9) | 1 (0.7 to 1.3) | 5.01 (2.6 to 7.46) |
| Deaths | Egypt | 310.2 (206.1 to 617.5) | 1.1 (0.7 to 2.2) | 641.5 (436.6 to 893.6) | 1.4 (0.9 to 1.9) | 0.79 (0.57 to 1.01) |
| Deaths | El Salvador | 27.1 (19.5 to 38.6) | 1 (0.7 to 1.4) | 21.6 (14 to 31.2) | 0.9 (0.6 to 1.3) | -0.35 (-0.54 to -0.16) |
| Deaths | Equatorial Guinea | 0.7 (0.3 to 1.4) | 0.3 (0.1 to 0.5) | 2.1 (0.9 to 4.3) | 0.3 (0.1 to 0.6) | 0.03 (-0.29 to 0.35) |
| Deaths | Eritrea | 14.6 (8.6 to 24.8) | 0.7 (0.4 to 1.2) | 30.9 (18 to 48.9) | 0.9 (0.5 to 1.5) | 1.05 (0.77 to 1.33) |
| Deaths | Estonia | 5.4 (4.4 to 6.4) | 1.2 (1 to 1.4) | 2.8 (2.2 to 3.4) | 1 (0.8 to 1.2) | -0.18 (-1.67 to 1.34) |
| Deaths | Eswatini | 1.8 (1.1 to 3) | 0.4 (0.2 to 0.6) | 2.9 (1.8 to 4.4) | 0.5 (0.3 to 0.8) | 1.31 (1.03 to 1.59) |
| Deaths | Ethiopia | 177 (82.8 to 575.8) | 0.6 (0.3 to 1.8) | 343.5 (194.3 to 631.4) | 0.6 (0.3 to 1.1) | 0.2 (-0.11 to 0.52) |
| Deaths | Fiji | 1.7 (0.7 to 2.6) | 0.5 (0.2 to 0.7) | 1.6 (0.8 to 2.5) | 0.5 (0.2 to 0.7) | 0.05 (-0.31 to 0.41) |
| Deaths | Finland | 11.2 (9.4 to 13.2) | 0.9 (0.7 to 1) | 7.8 (6.2 to 9.6) | 0.7 (0.5 to 0.8) | -1.31 (-1.72 to -0.9) |
| Deaths | France | 142.6 (127.6 to 159.9) | 0.9 (0.8 to 1) | 120.3 (98.8 to 143.9) | 0.8 (0.6 to 0.9) | -0.47 (-1.17 to 0.23) |
| Deaths | Gabon | 1.4 (0.8 to 2.3) | 0.3 (0.2 to 0.4) | 2.4 (1.4 to 4) | 0.3 (0.2 to 0.5) | 0.25 (-0.42 to 0.93) |
| Deaths | Gambia | 0.5 (0.3 to 0.9) | 0.1 (0 to 0.1) | 1.3 (0.6 to 2) | 0.1 (0 to 0.2) | 0.35 (-1.04 to 1.75) |
| Deaths | Georgia | 12.2 (8.2 to 17.3) | 0.7 (0.5 to 1) | 11.4 (8.6 to 14.6) | 1.2 (0.9 to 1.6) | 2.13 (0.97 to 3.31) |
| Deaths | Germany | 222.2 (200.6 to 246.2) | 1.3 (1.2 to 1.4) | 128.6 (108.3 to 150.3) | 0.8 (0.7 to 0.9) | -1.57 (-1.79 to -1.35) |
| Deaths | Ghana | 81.2 (47.9 to 120.1) | 0.9 (0.5 to 1.4) | 119.7 (64.5 to 194.7) | 0.7 (0.4 to 1.2) | -0.82 (-1.2 to -0.44) |
| Deaths | Greece | 40.3 (35.8 to 45.2) | 1.4 (1.3 to 1.6) | 21.3 (17.9 to 25.1) | 1.1 (0.9 to 1.3) | -0.7 (-1.7 to 0.32) |
| Deaths | Greenland | 0.4 (0.2 to 0.6) | 2.3 (1.3 to 3.4) | 0.1 (0.1 to 0.2) | 0.9 (0.6 to 1.4) | -2.82 (-3.21 to -2.42) |
| Deaths | Grenada | 0.3 (0.2 to 0.4) | 0.7 (0.5 to 1) | 0.2 (0.2 to 0.3) | 0.8 (0.6 to 1.1) | 0.78 (0.3 to 1.26) |
| Deaths | Guam | 0.1 (0.1 to 0.2) | 0.2 (0.1 to 0.3) | 0.1 (0.1 to 0.2) | 0.2 (0.1 to 0.4) | 0.16 (-1.27 to 1.62) |
| Deaths | Guatemala | 39.2 (33.7 to 45.1) | 0.8 (0.6 to 0.9) | 35.8 (28.5 to 44.6) | 0.5 (0.4 to 0.7) | -1.1 (-1.92 to -0.27) |
| Deaths | Guinea | 16.3 (8.7 to 24.9) | 0.4 (0.2 to 0.6) | 30.1 (12 to 52.2) | 0.4 (0.2 to 0.7) | -0.27 (-0.75 to 0.21) |
| Deaths | Guinea-Bissau | 1.6 (1 to 2.6) | 0.2 (0.1 to 0.4) | 3.7 (1.8 to 6.2) | 0.3 (0.2 to 0.5) | 0.99 (-0.36 to 2.35) |
| Deaths | Guyana | 1.2 (0.9 to 1.5) | 0.3 (0.2 to 0.4) | 1.5 (1.2 to 2) | 0.5 (0.4 to 0.7) | 2.07 (-0.26 to 4.45) |
| Deaths | Haiti | 41.4 (18 to 150.9) | 1.2 (0.5 to 4.2) | 68.7 (34.5 to 149.9) | 1.2 (0.6 to 2.7) | 0.18 (-0.11 to 0.47) |
| Deaths | Honduras | 32.9 (22.4 to 45.1) | 1.2 (0.8 to 1.6) | 34.7 (17.9 to 57) | 0.8 (0.4 to 1.3) | -1.24 (-1.36 to -1.13) |
| Deaths | Hungary | 34.5 (30.5 to 38.8) | 1.2 (1.1 to 1.4) | 10.4 (8.6 to 12.5) | 0.5 (0.5 to 0.7) | -2.71 (-3.85 to -1.57) |
| Deaths | Iceland | 1.5 (1.2 to 1.8) | 1.8 (1.5 to 2.1) | 0.8 (0.6 to 1.1) | 0.9 (0.7 to 1.1) | -1.65 (-3.06 to -0.22) |
| Deaths | India | 2782.3 (1681.6 to 4229.3) | 0.7 (0.4 to 1) | 2689.7 (1985.5 to 3689.3) | 0.5 (0.4 to 0.8) | -0.62 (-0.92 to -0.31) |
| Deaths | Indonesia | 564.7 (312.4 to 907) | 0.6 (0.4 to 1) | 619.8 (404.1 to 866.2) | 0.7 (0.4 to 1) | 0.2 (0 to 0.39) |
| Deaths | Iran (Islamic Republic of) | 495.8 (302.7 to 693) | 1.5 (0.9 to 2.1) | 359 (213.7 to 465) | 1.4 (0.8 to 1.8) | -0.36 (-0.63 to -0.08) |
| Deaths | Iraq | 129.4 (79 to 232.8) | 1.3 (0.8 to 2.2) | 229.7 (150.7 to 348.6) | 1.3 (0.9 to 2) | 0.05 (-0.18 to 0.29) |
| Deaths | Ireland | 15.4 (12.9 to 18.3) | 1.2 (1 to 1.4) | 8.1 (6.4 to 10.1) | 0.6 (0.5 to 0.8) | -2.09 (-2.88 to -1.3) |
| Deaths | Israel | 23.9 (20.2 to 28.1) | 1.2 (1 to 1.4) | 23.5 (18.5 to 29.5) | 0.7 (0.5 to 0.9) | -1.66 (-2.8 to -0.52) |
| Deaths | Italy | 168.6 (158.6 to 178.7) | 1.3 (1.2 to 1.4) | 73.9 (66 to 82.9) | 0.7 (0.6 to 0.8) | -2.37 (-2.9 to -1.83) |
| Deaths | Jamaica | 6.7 (4.8 to 9) | 0.6 (0.4 to 0.8) | 4.6 (3.3 to 6.2) | 0.6 (0.4 to 0.8) | -0.06 (-1.27 to 1.15) |
| Deaths | Japan | 167.4 (159.7 to 175.4) | 0.5 (0.5 to 0.5) | 124.1 (116.3 to 132) | 0.6 (0.5 to 0.6) | 0.57 (0 to 1.14) |
| Deaths | Jordan | 21.5 (14.3 to 31.1) | 1 (0.7 to 1.5) | 38.2 (25 to 56) | 0.8 (0.5 to 1.2) | -0.84 (-1.04 to -0.64) |
| Deaths | Kazakhstan | 66 (51.5 to 83.5) | 1 (0.8 to 1.3) | 49.4 (38.2 to 62.1) | 0.7 (0.6 to 0.9) | -1.02  (-1.49 to -0.55) |
| Deaths | Kenya | 68.6 (44.8 to 120.8) | 0.5 (0.3 to 0.8) | 144.4 (98.6 to 229.5) | 0.6 (0.4 to 1) | 0.72 (0.34 to 1.1) |
| Deaths | Kiribati | 0.1 (0 to 0.1) | 0.2 (0.1 to 0.4) | 0.1 (0 to 0.2) | 0.2 (0.1 to 0.4) | 0.03 (-0.39 to 0.46) |
| Deaths | Kuwait | 5.8 (4.7 to 7.1) | 0.8 (0.7 to 1) | 4.2 (3.1 to 5.5) | 0.4 (0.3 to 0.5) | -2.61 (-4.01 to -1.2) |
| Deaths | Kyrgyzstan | 9.8 (7.3 to 13.2) | 0.4 (0.3 to 0.6) | 23.9 (18.4 to 30.5) | 0.8 (0.6 to 1.1) | 2.05 (1.17 to 2.93) |
| Deaths | Lao People's Democratic Republic | 14.4 (6.9 to 36.9) | 0.6 (0.3 to 1.6) | 20.1 (11.7 to 31.8) | 0.7 (0.4 to 1.1) | 0.25 (-0.01 to 0.5) |
| Deaths | Latvia | 7.7 (6.3 to 9.3) | 1 (0.8 to 1.2) | 3.1 (2.4 to 3.9) | 0.8 (0.6 to 1) | -0.54 (-2.2 to 1.16) |
| Deaths | Lebanon | 10 (6 to 15.6) | 0.7 (0.4 to 1.2) | 11.6 (6.6 to 18.4) | 0.7 (0.4 to 1.1) | -0.12 (-0.52 to 0.29) |
| Deaths | Lesotho | 2.4 (1.5 to 3.9) | 0.3 (0.2 to 0.5) | 4 (2.4 to 6.4) | 0.5 (0.3 to 0.8) | 1.69 (1.32 to 2.06) |
| Deaths | Liberia | 3.3 (1.9 to 5.9) | 0.2 (0.1 to 0.4) | 8.3 (3.6 to 13.1) | 0.3 (0.1 to 0.5) | 0.87 (-0.09 to 1.84) |
| Deaths | Libya | 31.6 (18 to 47.3) | 1.4 (0.8 to 2) | 28.5 (16.5 to 44.2) | 1.4 (0.8 to 2.2) | 0.04 (-0.28 to 0.37) |
| Deaths | Lithuania | 11.4 (9.6 to 13.5) | 1 (0.9 to 1.2) | 5.2 (4.1 to 6.3) | 1 (0.8 to 1.2) | -0.04 (-1.32 to 1.24) |
| Deaths | Luxembourg | 1.3 (1.1 to 1.6) | 1.5 (1.3 to 1.8) | 1 (0.8 to 1.2) | 0.7 (0.6 to 0.9) | -2.22 (-3.28 to -1.15) |
| Deaths | Madagascar | 51.9 (33.5 to 79.8) | 0.7 (0.5 to 1.1) | 100.3 (57.8 to 159.5) | 0.7 (0.4 to 1.1) | -0.18 (-0.62 to 0.26) |
| Deaths | Malawi | 23.8 (12.9 to 37.2) | 0.4 (0.2 to 0.6) | 36.9 (16.6 to 62.5) | 0.4 (0.2 to 0.6) | -0.15 (-0.65 to 0.36) |
| Deaths | Malaysia | 42.3 (20.5 to 66.3) | 0.5 (0.2 to 0.8) | 46.3 (24.5 to 69.7) | 0.4 (0.2 to 0.7) | -0.31 (-0.94 to 0.33) |
| Deaths | Maldives | 0.8 (0.4 to 1.8) | 0.6 (0.3 to 1.3) | 0.7 (0.4 to 1) | 0.5 (0.3 to 0.8) | -0.36 (-0.99 to 0.27) |
| Deaths | Mali | 8.8 (3.6 to 14.3) | 0.1 (0.1 to 0.2) | 20.9 (7.3 to 35.8) | 0.1 (0 to 0.2) | -0.35 (-1.23 to 0.55) |
| Deaths | Malta | 1 (0.8 to 1.3) | 0.9 (0.7 to 1.1) | 1 (0.7 to 1.3) | 1.2 (0.9 to 1.6) | 1.21 (-0.55 to 3) |
| Deaths | Marshall Islands | 0.1 (0 to 0.1) | 0.2 (0.1 to 0.3) | 0.1 (0 to 0.1) | 0.3 (0.2 to 0.5) | 1.4 (1.08 to 1.71) |
| Deaths | Mauritania | 2.7 (1.7 to 4.2) | 0.2 (0.1 to 0.3) | 7.5 (3.5 to 11.9) | 0.3 (0.1 to 0.5) | 1.18 (0.78 to 1.58) |
| Deaths | Mauritius | 1.2 (1 to 1.4) | 0.3 (0.2 to 0.3) | 0.7 (0.6 to 0.8) | 0.2 (0.2 to 0.3) | -0.16 (-2.01 to 1.72) |
| Deaths | Mexico | 411 (373.4 to 453.8) | 1 (0.9 to 1.1) | 302.1 (256.5 to 356.3) | 0.7 (0.6 to 0.8) | -1.06 (-1.45 to -0.67) |
| Deaths | Micronesia (Federated States of) | 0.1 (0.1 to 0.2) | 0.3 (0.2 to 0.4) | 0.1 (0.1 to 0.2) | 0.3 (0.1 to 0.4) | 0.01 (-0.3 to 0.31) |
| Deaths | Monaco | 0.1 (0 to 0.1) | 1.4 (0.9 to 2.1) | 0.1 (0.1 to 0.2) | 1.8 (1.1 to 2.5) | 1.08 (0.74 to 1.41) |
| Deaths | Mongolia | 8.2 (4.8 to 17.2) | 0.7 (0.4 to 1.5) | 12.4 (7.8 to 17.7) | 0.9 (0.6 to 1.3) | 0.85 (0.24 to 1.45) |
| Deaths | Montenegro | 5.3 (3.8 to 7.2) | 2.5 (1.8 to 3.4) | 2.3 (1.3 to 3.5) | 1.5 (0.9 to 2.3) | -1.55 (-2.89 to -0.2) |
| Deaths | Morocco | 71.3 (38 to 113.2) | 0.6 (0.3 to 0.9) | 63.7 (31.6 to 102.6) | 0.5 (0.2 to 0.8) | -0.44 (-0.64 to -0.25) |
| Deaths | Mozambique | 70.1 (41.8 to 122.3) | 0.8 (0.5 to 1.4) | 148.5 (71.2 to 270.4) | 0.8 (0.4 to 1.5) | 0 (-0.65 to 0.66) |
| Deaths | Myanmar | 146.5 (67.1 to 326.2) | 0.8 (0.4 to 1.7) | 161.5 (98.3 to 245.2) | 0.8 (0.5 to 1.2) | 0.09 (-0.18 to 0.35) |
| Deaths | Namibia | 3.1 (1.6 to 4.7) | 0.4 (0.2 to 0.6) | 6.5 (3.6 to 10.5) | 0.6 (0.3 to 1) | 1.35 (0.97 to 1.72) |
| Deaths | Nauru | 0 (0 to 0) | 0.3 (0.2 to 0.5) | 0 (0 to 0) | 0.4 (0.2 to 0.6) | 0.62 (0.43 to 0.81) |
| Deaths | Nepal | 74.9 (39.9 to 154.7) | 0.7 (0.4 to 1.4) | 79.1 (48 to 130.3) | 0.6 (0.4 to 1.1) | -0.22 (-0.47 to 0.03) |
| Deaths | Netherlands | 37.7 (32.6 to 43.2) | 1 (0.9 to 1.2) | 25.2 (20.8 to 30.4) | 0.7 (0.6 to 0.8) | -1.12 (-1.58 to -0.66) |
| Deaths | New Zealand | 13.2 (11.2 to 15.5) | 1.2 (1 to 1.4) | 9.5 (7.9 to 11.3) | 0.7 (0.6 to 0.9) | -1.78 (-2.21 to -1.33) |
| Deaths | Nicaragua | 19.2 (13.3 to 29.3) | 0.8 (0.6 to 1.3) | 16.7 (10.6 to 25.4) | 0.6 (0.4 to 1) | -0.81 (-0.91 to -0.71) |
| Deaths | Niger | 12.7 (7.4 to 23.2) | 0.2 (0.1 to 0.4) | 44.2 (18.3 to 73.8) | 0.3 (0.1 to 0.4) | 0.49 (-0.15 to 1.12) |
| Deaths | Nigeria | 144.8 (86.1 to 293.7) | 0.3 (0.2 to 0.5) | 465 (200.1 to 683.9) | 0.4 (0.2 to 0.5) | 0.84 (0.66 to 1.03) |
| Deaths | Niue | 0 (0 to 0) | 0.3 (0.2 to 0.5) | 0 (0 to 0) | 1.7 (1 to 2.5) | 6.27 (5.6 to 6.95) |
| Deaths | North Macedonia | 17.7 (12.4 to 25.8) | 2.6 (1.8 to 3.8) | 7.9 (5.2 to 11.5) | 1.8 (1.2 to 2.6) | -1.22 (-1.83 to -0.6) |
| Deaths | Northern Mariana Islands | 0 (0 to 0.1) | 0.2 (0.1 to 0.4) | 0 (0 to 0.1) | 0.3 (0.2 to 0.5) | 1.37 (1 to 1.75) |
| Deaths | Norway | 16.8 (15.6 to 18) | 1.5 (1.4 to 1.7) | 11.4 (10.3 to 12.6) | 0.9 (0.8 to 1) | -1.41 (-2.64 to -0.16) |
| Deaths | Oman | 4.6 (2.4 to 7.4) | 0.4 (0.2 to 0.7) | 5.6 (3.1 to 8.6) | 0.4 (0.2 to 0.6) | -0.61 (-0.87 to -0.35) |
| Deaths | Pakistan | 489.4 (277.9 to 809.5) | 0.8 (0.4 to 1.3) | 1063 (717.9 to 1575.4) | 1 (0.7 to 1.4) | 0.69 (0.44 to 0.93) |
| Deaths | Palau | 0 (0 to 0) | 0.2 (0.1 to 0.3) | 0 (0 to 0) | 0.2 (0.1 to 0.3) | -0.25 (-0.46 to -0.04) |
| Deaths | Palestine | 20.7 (12.9 to 34.4) | 1.7 (1.1 to 2.8) | 34.9 (23.5 to 53.5) | 1.4 (1 to 2.2) | -0.47 (-1.04 to 0.11) |
| Deaths | Panama | 16.8 (13.6 to 20.6) | 1.6 (1.3 to 1.9) | 12.1 (9.2 to 15.7) | 0.8 (0.6 to 1) | -2.29 (-3.28 to -1.3) |
| Deaths | Papua New Guinea | 4.6 (1.8 to 9.6) | 0.2 (0.1 to 0.4) | 13.8 (6.6 to 24.4) | 0.3 (0.1 to 0.5) | 0.83 (0.22 to 1.45) |
| Deaths | Paraguay | 10.2 (7.1 to 15.4) | 0.5 (0.3 to 0.7) | 18.9 (11.3 to 28.7) | 0.7 (0.4 to 1.1) | 1.55 (1.29 to 1.81) |
| Deaths | Peru | 185.8 (124.1 to 288.8) | 1.7 (1.2 to 2.7) | 156.6 (100.8 to 236.5) | 1.3 (0.8 to 1.9) | -1.04 (-1.38 to -0.69) |
| Deaths | Philippines | 229.6 (145 to 337.2) | 0.7 (0.5 to 1) | 295.8 (214.8 to 378.9) | 0.7 (0.5 to 0.9) | -0.23 (-0.5 to 0.05) |
| Deaths | Poland | 213.4 (203.5 to 223.4) | 1.7 (1.7 to 1.8) | 60.1 (53.7 to 66.7) | 0.8 (0.7 to 0.9) | -2.54 (-3.42 to -1.66) |
| Deaths | Portugal | 51.1 (43.9 to 59.2) | 1.8 (1.5 to 2.1) | 15.8 (12.8 to 19.5) | 0.8 (0.7 to 1) | -2.55 (-3.47 to -1.63) |
| Deaths | Puerto Rico | 8.2 (6.4 to 10.3) | 0.6 (0.5 to 0.8) | 2.7 (2.1 to 3.4) | 0.4 (0.3 to 0.5) | -0.96 (-1.92 to 0.01) |
| Deaths | Qatar | 0.8 (0.5 to 1.2) | 0.5 (0.3 to 0.8) | 2.3 (1.4 to 3.6) | 0.4 (0.2 to 0.6) | -0.93 (-1.3 to -0.56) |
| Deaths | Republic of Korea | 196 (128 to 250.6) | 1.2 (0.8 to 1.6) | 58.3 (34.8 to 78.7) | 0.7 (0.4 to 1) | -1.8 (-2.07 to -1.54) |
| Deaths | Republic of Moldova | 30.9 (26.1 to 36.3) | 1.9 (1.6 to 2.3) | 7.9 (6.3 to 9.8) | 1.2 (0.9 to 1.5) | -1.79 (-2.62 to -0.95) |
| Deaths | Romania | 140.9 (122.9 to 159.3) | 2 (1.7 to 2.2) | 40.1 (32.6 to 48.3) | 1 (0.8 to 1.2) | -1.82 (-2.1 to -1.54) |
| Deaths | Russian Federation | 682.3 (656.8 to 710.1) | 1.5 (1.5 to 1.6) | 299.5 (274.1 to 322.7) | 0.9 (0.8 to 1) | -1.88 (-2.82 to -0.93) |
| Deaths | Rwanda | 42 (25.6 to 66) | 0.9 (0.6 to 1.5) | 54.1 (30.9 to 83.8) | 0.8 (0.5 to 1.3) | -0.39 (-1.55 to 0.79) |
| Deaths | Saint Kitts and Nevis | 0.1 (0.1 to 0.2) | 0.7 (0.5 to 0.9) | 0.1 (0.1 to 0.2) | 0.9 (0.7 to 1.1) | 0.87 (0.48 to 1.27) |
| Deaths | Saint Lucia | 0.4 (0.3 to 0.5) | 0.6 (0.4 to 0.8) | 0.3 (0.2 to 0.4) | 0.8 (0.6 to 1) | 0.9 (0.33 to 1.47) |
| Deaths | Saint Vincent and the Grenadines | 0.4 (0.3 to 0.5) | 0.7 (0.5 to 0.9) | 0.3 (0.3 to 0.4) | 0.9 (0.7 to 1.2) | 1.11 (-0.86 to 3.12) |
| Deaths | Samoa | 0.5 (0.3 to 0.8) | 0.6 (0.3 to 0.9) | 0.6 (0.3 to 0.9) | 0.5 (0.3 to 0.9) | -0.03 (-0.4 to 0.34) |
| Deaths | San Marino | 0.1 (0.1 to 0.2) | 2.3 (1.5 to 3.4) | 0.1 (0.1 to 0.1) | 1.4 (0.9 to 2.1) | -1.52 (-1.77 to -1.27) |
| Deaths | Sao Tome and Principe | 0.2 (0.1 to 0.2) | 0.2 (0.1 to 0.3) | 0.1 (0.1 to 0.2) | 0.1 (0.1 to 0.2) | -1.39 (-2.96 to 0.21) |
| Deaths | Saudi Arabia | 60.5 (36.5 to 94.9) | 0.7 (0.4 to 1.1) | 37 (21.8 to 63.1) | 0.4 (0.2 to 0.6) | -2.24 (-2.35 to -2.14) |
| Deaths | Senegal | 12.9 (8.9 to 19.5) | 0.3 (0.2 to 0.4) | 30.6 (14.9 to 46.8) | 0.4 (0.2 to 0.6) | 1.07 (0.24 to 1.9) |
| Deaths | Serbia | 63.9 (41 to 96.6) | 2.3 (1.4 to 3.4) | 16.9 (11 to 25.9) | 0.9 (0.6 to 1.4) | -2.85 (-3.31 to -2.38) |
| Deaths | Seychelles | 0.2 (0.2 to 0.3) | 0.7 (0.5 to 1.1) | 0.1 (0.1 to 0.2) | 0.4 (0.2 to 0.6) | -1.7 (-3.73 to 0.36) |
| Deaths | Sierra Leone | 5.9 (3.5 to 9.6) | 0.2 (0.1 to 0.4) | 16.6 (7.3 to 25.9) | 0.4 (0.2 to 0.6) | 1.44 (0.46 to 2.43) |
| Deaths | Singapore | 4.9 (4 to 6) | 0.6 (0.5 to 0.7) | 6.4 (5.1 to 7.8) | 0.6 (0.5 to 0.8) | 0.05 (-1.9 to 2.03) |
| Deaths | Slovakia | 20.6 (15.1 to 28.4) | 1.2 (0.9 to 1.6) | 11 (7.3 to 15.8) | 1 (0.7 to 1.4) | -0.55 (-0.98 to -0.13) |
| Deaths | Slovenia | 4.8 (4 to 5.6) | 0.9 (0.7 to 1) | 1.8 (1.4 to 2.3) | 0.4 (0.3 to 0.6) | -2.4 (-3.59 to -1.19) |
| Deaths | Solomon Islands | 0.4 (0.1 to 0.6) | 0.2 (0.1 to 0.3) | 0.9 (0.4 to 1.4) | 0.3 (0.1 to 0.4) | 1.02 (0.28 to 1.77) |
| Deaths | Somalia | 24.5 (12.1 to 47.1) | 0.5 (0.2 to 0.9) | 53.9 (23 to 93.5) | 0.4 (0.2 to 0.7) | -0.37 (-0.9 to 0.17) |
| Deaths | South Africa | 64.7 (47.5 to 99.9) | 0.4 (0.3 to 0.6) | 82.6 (61.8 to 113.7) | 0.4 (0.3 to 0.6) | 0.42 (0.08 to 0.75) |
| Deaths | South Sudan | 24.2 (14 to 42.4) | 0.7 (0.4 to 1.2) | 39.8 (23.4 to 63.6) | 0.7 (0.4 to 1.1) | 0.28 (-0.36 to 0.92) |
| Deaths | Spain | 125.2 (110.2 to 141.7) | 1.1 (1 to 1.3) | 79.8 (66.1 to 93.9) | 0.9 (0.7 to 1.1) | -1.03 (-1.16 to -0.91) |
| Deaths | Sri Lanka | 40.4 (27.6 to 59.1) | 0.6 (0.4 to 0.8) | 35 (21.1 to 54) | 0.5 (0.3 to 0.8) | -0.45 (-0.84 to -0.05) |
| Deaths | Sudan | 141.3 (58.5 to 391.1) | 1.2 (0.5 to 3.3) | 284.5 (157.8 to 456.4) | 1.3 (0.7 to 2.1) | 0.25 (0.09 to 0.41) |
| Deaths | Suriname | 2.5 (1.4 to 3.6) | 1.5 (0.8 to 2.2) | 3.2 (2 to 4.6) | 1.7 (1.1 to 2.5) | 0.44 (-0.26 to 1.13) |
| Deaths | Sweden | 25.7 (22.2 to 29.5) | 1.2 (1.1 to 1.4) | 17.2 (14.4 to 20.7) | 0.7 (0.6 to 0.9) | -1.79 (-2.94 to -0.62) |
| Deaths | Switzerland | 14 (11.9 to 16.5) | 0.9 (0.8 to 1.1) | 11.7 (9.4 to 14.2) | 0.7 (0.5 to 0.8) | -1.07 (-2.02 to -0.12) |
| Deaths | Syrian Arab Republic | 120.8 (76.7 to 197.2) | 1.6 (1 to 2.6) | 70.1 (46.1 to 102.8) | 1.2 (0.8 to 1.8) | -0.9 (-1.21 to -0.58) |
| Deaths | Taiwan (Province of China) | 59.3 (51.9 to 67.7) | 0.8 (0.7 to 0.9) | 21.6 (17.4 to 26.5) | 0.5 (0.4 to 0.6) | -1.31 (-2.71 to 0.11) |
| Deaths | Tajikistan | 73 (40.3 to 130.6) | 2.4 (1.4 to 4.3) | 110.9 (66.9 to 177.5) | 2.4 (1.5 to 3.9) | -0.01 (-0.26 to 0.23) |
| Deaths | Thailand | 202.7 (114.3 to 292.4) | 0.9 (0.5 to 1.3) | 86.8 (56.6 to 120.9) | 0.6 (0.4 to 0.9) | -1.13 (-1.41 to -0.84) |
| Deaths | Timor-Leste | 2.5 (1.1 to 6.3) | 0.6 (0.3 to 1.4) | 4.1 (2.5 to 6.6) | 0.6 (0.4 to 1) | 0.12 (-0.36 to 0.59) |
| Deaths | Togo | 5 (3.3 to 7.2) | 0.2 (0.1 to 0.3) | 12.7 (5.7 to 20.1) | 0.3 (0.1 to 0.5) | 1.1 (0.62 to 1.58) |
| Deaths | Tokelau | 0 (0 to 0) | 0.3 (0.2 to 0.5) | 0 (0 to 0) | 2.4 (1.2 to 3.7) | 8.32 (7.52 to 9.13) |
| Deaths | Tonga | 0.2 (0.1 to 0.3) | 0.3 (0.2 to 0.6) | 0.2 (0.1 to 0.3) | 0.4 (0.2 to 0.7) | 0.51 (0.26 to 0.76) |
| Deaths | Trinidad and Tobago | 3.1 (2.4 to 3.8) | 0.6 (0.5 to 0.7) | 2.1 (1.6 to 2.7) | 0.6 (0.4 to 0.7) | 0.01 (-1.27 to 1.31) |
| Deaths | Tunisia | 42 (23.4 to 64.6) | 1.1 (0.6 to 1.6) | 25.3 (13.5 to 38.9) | 0.7 (0.4 to 1.1) | -1.28 (-1.47 to -1.09) |
| Deaths | Turkey | 553.2 (331.9 to 884.3) | 2.1 (1.3 to 3.3) | 293.3 (197.3 to 410.8) | 1.2 (0.8 to 1.7) | -1.85 (-2.08 to -1.61) |
| Deaths | Turkmenistan | 12.2 (9.7 to 14.9) | 0.6 (0.5 to 0.8) | 41.2 (31.5 to 52.2) | 2.1 (1.6 to 2.7) | 4.02 (3.16 to 4.89) |
| Deaths | Tuvalu | 0 (0 to 0) | 0.3 (0.1 to 0.5) | 0 (0 to 0) | 0.3 (0.1 to 0.4) | 0.1 (-0.08 to 0.28) |
| Deaths | Uganda | 93.6 (50.1 to 149.6) | 0.8 (0.4 to 1.3) | 258.9 (133.5 to 420.5) | 1 (0.5 to 1.6) | 0.74 (-0.06 to 1.55) |
| Deaths | Ukraine | 198.1 (142.9 to 264.4) | 1.3 (1 to 1.8) | 105.1 (79.5 to 132.8) | 1.3 (1 to 1.6) | 0.01 (-0.61 to 0.63) |
| Deaths | United Arab Emirates | 5.6 (3 to 11.2) | 0.8 (0.5 to 1.6) | 9.9 (6.1 to 16.5) | 0.6 (0.4 to 1) | -1.11 (-2.01 to -0.21) |
| Deaths | United Kingdom | 159.8 (154.1 to 165.6) | 1.1 (1.1 to 1.1) | 100.1 (93.1 to 106.3) | 0.6 (0.6 to 0.7) | -1.55 (-1.98 to -1.11) |
| Deaths | United Republic of Tanzania | 147.4 (88.3 to 216.6) | 0.9 (0.5 to 1.3) | 316.2 (181.9 to 487.6) | 1 (0.6 to 1.5) | 0.25 (0.02 to 0.49) |
| Deaths | United States of America | 724.4 (704.4 to 744) | 1 (1 to 1) | 591.7 (550.8 to 636.5) | 0.7 (0.7 to 0.8) | -0.97 (-1.38 to -0.55) |
| Deaths | United States Virgin Islands | 0.2 (0.2 to 0.4) | 0.6 (0.4 to 1) | 0.1 (0 to 0.1) | 0.4 (0.2 to 0.8) | -1.15 (-1.83 to -0.46) |
| Deaths | Uruguay | 9.8 (8 to 12) | 0.9 (0.7 to 1.1) | 7 (5.3 to 9.1) | 0.8 (0.6 to 1) | -0.91 (-1.53 to -0.28) |
| Deaths | Uzbekistan | 152.8 (102.3 to 214.1) | 1.4 (0.9 to 2) | 233.2 (181.6 to 294.7) | 1.8 (1.4 to 2.3) | 0.83 (0.44 to 1.23) |
| Deaths | Vanuatu | 0.1 (0.1 to 0.2) | 0.2 (0.1 to 0.3) | 0.3 (0.2 to 0.5) | 0.2 (0.1 to 0.4) | 0.99 (-0.62 to 2.62) |
| Deaths | Venezuela (Bolivarian Republic of) | 32.1 (28.4 to 36.3) | 0.4 (0.3 to 0.4) | 60 (43.8 to 79.1) | 0.7 (0.5 to 0.9) | 2.19 (1.3 to 3.09) |
| Deaths | Viet Nam | 147.2 (80 to 226) | 0.4 (0.2 to 0.7) | 131.6 (74.9 to 201.5) | 0.4 (0.2 to 0.6) | -0.15 (-0.46 to 0.15) |
| Deaths | Yemen | 69.2 (28.6 to 178.5) | 0.8 (0.3 to 1.9) | 163.1 (88.4 to 255.5) | 0.9 (0.5 to 1.5) | 0.58 (0.37 to 0.8) |
| Deaths | Zambia | 46.4 (28.5 to 69.2) | 0.9 (0.6 to 1.4) | 93.5 (51.4 to 145.4) | 0.9 (0.5 to 1.4) | -0.02 (-0.31 to 0.26) |
| Deaths | Zimbabwe | 27.3 (15.5 to 40) | 0.4 (0.3 to 0.7) | 68.5 (40.3 to 104.1) | 0.9 (0.5 to 1.3) | 1.71 (0.59 to 2.84) |
| DALYs | Afghanistan | 4421.2 (1807.3 to 14979.7) | 79.6 (32.9 to 259.5) | 16893.4 (8824.4 to 29648.3) | 94.6 (49.5 to 165.1) | 0.59 (-0.06 to 1.25) |
| DALYs | Albania | 3014.7 (1912.9 to 4193.3) | 207.9 (132.2 to 289.1) | 990.9 (555.6 to 1515.6) | 169.7 (95.3 to 259.5) | -0.63 (-0.86 to -0.41) |
| DALYs | Algeria | 11128.9 (5658.6 to 16929.9) | 81 (41.3 to 123.2) | 9166.1 (4784.6 to 13716.4) | 54.6 (28.7 to 81.6) | -1.27 (-1.38 to -1.15) |
| DALYs | American Samoa | 5.4 (3.4 to 8.7) | 22.6 (14.5 to 36.6) | 6.1 (3.8 to 9) | 31.8 (19.9 to 47.3) | 1.16 (0.51 to 1.82) |
| DALYs | Andorra | 20 (11.1 to 34.4) | 159.2 (86.6 to 278.7) | 11.9 (7.5 to 18.1) | 84.4 (53.2 to 127.5) | -2.03 (-2.47 to -1.59) |
| DALYs | Angola | 1521.9 (642.3 to 3895.5) | 24.6 (10.9 to 60.4) | 4461.8 (2536.8 to 7352.9) | 23.3 (13.3 to 38.3) | -0.14 (-0.52 to 0.25) |
| DALYs | Antigua and Barbuda | 12.4 (9.2 to 16.1) | 52.5 (38.9 to 68.1) | 16.4 (13.4 to 20.2) | 72.9 (59.1 to 90) | 1.22 (0.72 to 1.72) |
| DALYs | Argentina | 9351.3 (7603.4 to 11446.6) | 72 (58.6 to 88.1) | 8952.7 (7272 to 10840.4) | 65.9 (53.5 to 79.9) | -0.31 (-0.8 to 0.19) |
| DALYs | Armenia | 1929.6 (1363.7 to 2600.6) | 143.3 (101.7 to 192.7) | 905 (696.9 to 1173.8) | 119 (91.2 to 155.2) | -0.74 (-1.76 to 0.28) |
| DALYs | Australia | 4508.9 (3944.1 to 5113.8) | 88.8 (77.8 to 100.6) | 3481.8 (2855.4 to 4186.2) | 55.8 (45.7 to 67.1) | -1.56 (-2 to -1.12) |
| DALYs | Austria | 1451.1 (1226.2 to 1691.6) | 79.3 (67.1 to 92.3) | 895.6 (728.4 to 1099.1) | 51.2 (41.6 to 62.9) | -1.34 (-2.33 to -0.33) |
| DALYs | Azerbaijan | 5719.9 (3509.2 to 8867.9) | 181.7 (111.9 to 280.9) | 5406.8 (3286 to 8430.2) | 178 (107.7 to 277.8) | -0.03 (-0.4 to 0.35) |
| DALYs | Bahamas | 47.4 (35.7 to 62.6) | 44.7 (33.5 to 59.2) | 61.4 (44.5 to 81.7) | 54.2 (38.9 to 73.1) | 0.78 (-0.62 to 2.2) |
| DALYs | Bahrain | 100.5 (68.6 to 147.7) | 49.8 (33.9 to 73) | 123.5 (80.2 to 195.2) | 30.1 (19.6 to 47.6) | -1.6 (-2.03 to -1.17) |
| DALYs | Bangladesh | 46738.4 (24693.1 to 97438.6) | 74.4 (39.3 to 153.4) | 44025.9 (28175.1 to 67060.5) | 72.7 (46.4 to 111) | -0.07 (-0.36 to 0.22) |
| DALYs | Barbados | 68.3 (52 to 85.9) | 81.4 (61.7 to 102.7) | 43.8 (30.6 to 60.5) | 66.7 (46.1 to 92.7) | -0.79 (-1.88 to 0.31) |
| DALYs | Belarus | 1785.7 (1408.9 to 2202.3) | 57 (45 to 70.3) | 1321.6 (983.6 to 1734.5) | 64.8 (47.8 to 85.8) | 0.38 (-1.35 to 2.13) |
| DALYs | Belgium | 2458.1 (2096.7 to 2862.6) | 102 (87.1 to 118.8) | 2101.4 (1705.4 to 2573.9) | 83.1 (67.2 to 101.9) | -1.06 (-2.97 to 0.89) |
| DALYs | Belize | 70.4 (53.3 to 88.7) | 66.8 (50.5 to 84.3) | 89.3 (71.5 to 110.4) | 54.3 (43.3 to 67.5) | -0.71 (-1.29 to -0.12) |
| DALYs | Benin | 694.3 (436.6 to 1055.6) | 20.8 (13.1 to 31.6) | 2621.7 (1134.4 to 4337.3) | 32.5 (14.1 to 53.6) | 1.49 (0.95 to 2.02) |
| DALYs | Bermuda | 10.6 (7.1 to 14.6) | 67.5 (44.9 to 92.9) | 8.9 (6.5 to 11.8) | 81.4 (58.9 to 108) | 0.52 (0.17 to 0.86) |
| DALYs | Bhutan | 187.4 (59.6 to 346.5) | 54.6 (17.2 to 101.1) | 162.9 (96 to 269.7) | 66.4 (39.2 to 110) | 0.94 (0.27 to 1.62) |
| DALYs | Bolivia (Plurinational State of) | 3589.9 (2100 to 6518.4) | 103.6 (60.9 to 186.8) | 5506.6 (3366.1 to 8295.8) | 122.2 (74.7 to 183.9) | 0.53 (0.48 to 0.58) |
| DALYs | Bosnia and Herzegovina | 1882.5 (1349.2 to 2714.7) | 128.7 (92 to 185.8) | 670.2 (460.3 to 934.1) | 98.6 (67.8 to 137.6) | -0.96 (-1.39 to -0.52) |
| DALYs | Botswana | 207.2 (127.4 to 333.4) | 27.8 (17.1 to 44.6) | 372 (229.5 to 586.5) | 41.1 (25.3 to 64.9) | 1.27 (0.85 to 1.69) |
| DALYs | Brazil | 72941.4 (61886.9 to 84172.6) | 110.6 (93.6 to 128) | 56770.8 (46887.5 to 66302.2) | 89.5 (73.6 to 104.9) | -0.7 (-1.02 to -0.38) |
| DALYs | Brunei Darussalam | 108.3 (70.7 to 170.1) | 94.6 (61.5 to 149) | 103 (67.7 to 149.1) | 81.5 (53.6 to 117.8) | -0.53 (-0.9 to -0.16) |
| DALYs | Bulgaria | 3769.3 (3174.7 to 4376.6) | 164.8 (138.6 to 191.4) | 1367.4 (1097.1 to 1690.1) | 105.8 (84.4 to 131.4) | -1.55 (-3.75 to 0.7) |
| DALYs | Burkina Faso | 1130.2 (684.3 to 1843) | 17.6 (10.7 to 28.6) | 4153.7 (1866 to 6413) | 29.7 (13.4 to 46) | 1.77 (1.44 to 2.09) |
| DALYs | Burundi | 2306.6 (1279.3 to 3857.6) | 65.7 (36.6 to 109.3) | 4084.3 (1914.1 to 7108.2) | 54.4 (25.7 to 94.2) | -0.65 (-1.12 to -0.17) |
| DALYs | Cabo Verde | 79.3 (46.8 to 148.7) | 38.7 (22.9 to 72.5) | 131.9 (77 to 215.3) | 71.4 (41.4 to 117.6) | 2.08 (1.57 to 2.6) |
| DALYs | Cambodia | 3214.4 (1603.7 to 6620.3) | 55.2 (27.8 to 111.3) | 3758 (2275.6 to 5708) | 56.7 (34.4 to 86.1) | 0.14 (0.01 to 0.27) |
| DALYs | Cameroon | 1305.9 (819.1 to 1948) | 19.5 (12.3 to 29.1) | 5064.5 (2322.1 to 7932.5) | 29.2 (13.4 to 45.8) | 1.31 (0.72 to 1.9) |
| DALYs | Canada | 6105 (5425.6 to 6836.3) | 80.4 (71.5 to 90) | 4711.3 (3852.2 to 5727.3) | 56.9 (46.5 to 69.3) | -1.16 (-1.77 to -0.55) |
| DALYs | Central African Republic | 358.7 (178 to 835.1) | 22.7 (11.6 to 51.2) | 661.9 (346.1 to 1342.5) | 22.6 (11.9 to 45.5) | -0.14 (-0.35 to 0.08) |
| DALYs | Chad | 604.4 (361.4 to 1040.5) | 14.8 (8.8 to 25.3) | 3180.8 (1655.4 to 5063.7) | 26.6 (13.8 to 42.3) | 1.94 (1.69 to 2.19) |
| DALYs | Chile | 1762.7 (1447 to 2123.1) | 33.9 (27.8 to 40.9) | 2144.5 (1703.4 to 2687.7) | 43.6 (34.5 to 54.7) | 0.91 (-0.35 to 2.18) |
| DALYs | China | 829120.7 (549763.1 to 1073681.7) | 195.5 (129.3 to 253.3) | 297408.5 (217203.4 to 408284.7) | 88.8 (64.5 to 122.2) | -2.64 (-2.88 to -2.4) |
| DALYs | Colombia | 12264.5 (10295.8 to 14528.5) | 81.6 (68.5 to 96.6) | 10201.2 (7906.7 to 12911.5) | 70.8 (54.6 to 90) | -0.61 (-1.16 to -0.06) |
| DALYs | Comoros | 195.1 (94.9 to 288.6) | 69.1 (33.1 to 102.8) | 242.5 (140.1 to 377.1) | 78.9 (45.5 to 122.8) | 0.27 (-1.23 to 1.8) |
| DALYs | Congo | 322.2 (171.4 to 584.7) | 23.7 (12.7 to 42.7) | 574.6 (348.7 to 861.1) | 23.2 (14 to 34.8) | 0.03 (-0.5 to 0.55) |
| DALYs | Cook Islands | 0.5 (0.3 to 0.8) | 5.4 (3.2 to 8.9) | 0.4 (0.2 to 0.6) | 7.7 (4.2 to 13) | 1.44 (0.29 to 2.6) |
| DALYs | Costa Rica | 811.5 (692.3 to 947) | 56.5 (48.2 to 66) | 649.9 (518.5 to 812.8) | 46.9 (37.4 to 58.8) | -0.49 (-1.46 to 0.48) |
| DALYs | Coted'Ivoire | 1543.5 (756.8 to 2340) | 19.8 (9.9 to 30) | 3404.7 (1406.4 to 5576.6) | 22.4 (9.4 to 36.6) | 0.38 (-0.12 to 0.87) |
| DALYs | Croatia | 1556.5 (1298.4 to 1846) | 119.1 (99.3 to 141.4) | 649.8 (510.2 to 836.5) | 81.1 (63.4 to 105) | -1.34 (-2.79 to 0.13) |
| DALYs | Cuba | 2921.3 (2355.9 to 3643.9) | 82.5 (66.6 to 102.7) | 1876.9 (1447.8 to 2363.7) | 78.2 (60.1 to 98.7) | -0.23 (-0.87 to 0.42) |
| DALYs | Cyprus | 176.4 (120.2 to 264.5) | 68.4 (46.6 to 102.9) | 135.7 (82.7 to 202.6) | 47.8 (29.2 to 71.3) | -1.17 (-1.71 to -0.62) |
| DALYs | Czechia | 2887.3 (2480.7 to 3354.2) | 97.2 (83.7 to 112.7) | 1069.6 (838.1 to 1362.1) | 48 (37.6 to 61.2) | -2.07 (-2.81 to -1.33) |
| DALYs | Democratic People's Republic of Korea | 9744.1 (5447.5 to 15194.8) | 122.1 (68.3 to 190.3) | 5830.5 (3400.6 to 9200.6) | 91.9 (53.4 to 145.5) | -0.9 (-1 to -0.81) |
| DALYs | Democratic Republic of the Congo | 4778.6 (2415.7 to 9808.8) | 20.8 (10.8 to 41.4) | 9168 (5451 to 14478.5) | 19 (11.3 to 30) | -0.3 (-0.52 to -0.07) |
| DALYs | Denmark | 1098.7 (935.9 to 1289.4) | 92.9 (79.3 to 108.9) | 668.4 (532.8 to 836.5) | 52.5 (41.8 to 65.8) | -1.67 (-2.07 to -1.26) |
| DALYs | Djibouti | 177.7 (107.5 to 264.5) | 77.1 (46.6 to 114.8) | 354.7 (190.1 to 579.6) | 66.6 (35.7 to 108.9) | -0.26 (-1 to 0.49) |
| DALYs | Dominica | 12 (7.9 to 18.3) | 37 (24.3 to 56.2) | 13 (8.3 to 19.2) | 72.8 (45.6 to 108.6) | 2.22 (1.87 to 2.57) |
| DALYs | Dominican Republic | 3778.2 (2290.7 to 5796.3) | 107.5 (65.4 to 164.6) | 3779.1 (2429.2 to 5503.1) | 98.4 (63.2 to 143.3) | -0.23 (-0.45 to -0.01) |
| DALYs | Ecuador | 925.4 (723.6 to 1163.2) | 18.6 (14.6 to 23.4) | 5224.3 (3979.5 to 6869.7) | 79 (60 to 104.1) | 4.96 (2.56 to 7.41) |
| DALYs | Egypt | 25583.3 (16955.4 to 51338.1) | 90.6 (60.1 to 180.3) | 52351.7 (35656.1 to 72987.1) | 112 (76.2 to 156.1) | 0.76 (0.54 to 0.98) |
| DALYs | El Salvador | 2240.8 (1612 to 3189.2) | 81 (58.3 to 115.3) | 1745.7 (1128.3 to 2523.7) | 73 (47.1 to 105.9) | -0.42 (-0.6 to -0.23) |
| DALYs | Equatorial Guinea | 56 (27.3 to 117.2) | 22.1 (11.1 to 44.9) | 167.4 (72.8 to 346.8) | 21.9 (9.5 to 45.2) | -0.01 (-0.33 to 0.32) |
| DALYs | Eritrea | 1233.2 (724.7 to 2096.2) | 58.5 (34.3 to 99.1) | 2595 (1510.6 to 4117.4) | 78.9 (46 to 125.1) | 1.05 (0.76 to 1.34) |
| DALYs | Estonia | 438.5 (360.8 to 526) | 96.2 (79.2 to 115.3) | 222 (177.2 to 274.4) | 78.7 (62.6 to 97.5) | -0.44 (-2.33 to 1.49) |
| DALYs | Eswatini | 144.7 (89.9 to 246.5) | 29.7 (18.5 to 50.3) | 238.2 (146.8 to 358.7) | 44.6 (27.5 to 67.2) | 1.29 (1.01 to 1.58) |
| DALYs | Ethiopia | 14894.1 (6927.4 to 49150.5) | 46.9 (22.1 to 149.3) | 28544 (16083.2 to 52988.1) | 49.6 (28 to 92) | 0.19 (-0.14 to 0.51) |
| DALYs | Fiji | 135.9 (58.1 to 207.5) | 37.5 (16 to 57.3) | 132.9 (68.8 to 205.3) | 37.9 (19.6 to 58.5) | 0.07 (-0.36 to 0.5) |
| DALYs | Finland | 922.1 (776 to 1082.9) | 72.7 (61.2 to 85.3) | 639.1 (509.3 to 794.4) | 54.8 (43.6 to 68.2) | -1.29 (-1.71 to -0.87) |
| DALYs | France | 11606.3 (10383.3 to 13000.8) | 73.4 (65.7 to 82.1) | 9850.3 (8072.2 to 11792.9) | 64 (52.4 to 76.6) | -0.46 (-1.17 to 0.26) |
| DALYs | Gabon | 116 (66.7 to 191.8) | 22.2 (12.9 to 36.4) | 199 (112.3 to 327.2) | 24.1 (13.6 to 39.8) | 0.25 (-0.33 to 0.84) |
| DALYs | Gambia | 46.9 (24.6 to 73.3) | 7.4 (3.9 to 11.5) | 105.2 (48.2 to 169.7) | 8.2 (3.8 to 13.2) | 0.31 (-1.08 to 1.72) |
| DALYs | Georgia | 995.5 (671.4 to 1416.3) | 55.9 (37.7 to 79.5) | 910.4 (683.7 to 1176.5) | 99 (74.4 to 127.8) | 2.05 (0.87 to 3.25) |
| DALYs | Germany | 18181.1 (16407 to 20111.6) | 106.6 (96.2 to 117.9) | 10494.7 (8852.4 to 12274.8) | 66.2 (55.9 to 77.4) | -1.58 (-1.8 to -1.35) |
| DALYs | Ghana | 6800.5 (4025.1 to 10036.4) | 78 (45.8 to 115.4) | 9876.7 (5306.5 to 16050.5) | 59.9 (32.2 to 97.4) | -0.85 (-1.24 to -0.46) |
| DALYs | Greece | 3279 (2917.5 to 3672) | 119.4 (106.4 to 133.4) | 1741.7 (1460.4 to 2050.6) | 93 (77.7 to 109.7) | -0.69 (-1.69 to 0.33) |
| DALYs | Greenland | 33.8 (19.1 to 49.8) | 185.9 (105.3 to 275) | 11.8 (7.7 to 17.7) | 77.6 (50.9 to 116.4) | -2.8 (-3.19 to -2.41) |
| DALYs | Grenada | 24.4 (16.4 to 35.6) | 57.3 (38.3 to 83.5) | 20.1 (15.1 to 25.9) | 68.3 (51.1 to 88.5) | 0.77 (0.29 to 1.25) |
| DALYs | Guam | 9.1 (6.2 to 13.6) | 17.1 (11.6 to 25.6) | 9.3 (4.5 to 14.5) | 19.3 (9.3 to 30) | 0.26 (-1.13 to 1.66) |
| DALYs | Guatemala | 3287.8 (2831 to 3787) | 63 (54.2 to 72.6) | 2911.6 (2312.7 to 3636.1) | 44.8 (35.4 to 56.2) | -1.15 (-1.96 to -0.34) |
| DALYs | Guinea | 1402.1 (750.8 to 2146.3) | 35.6 (19.1 to 54.5) | 2560.4 (1013.9 to 4455) | 32.1 (12.9 to 55.7) | -0.3 (-0.79 to 0.19) |
| DALYs | Guinea-Bissau | 135.5 (81 to 223.6) | 21.1 (12.6 to 34.8) | 310.2 (145.5 to 510.6) | 26.9 (12.7 to 44.5) | 0.92 (-0.47 to 2.34) |
| DALYs | Guyana | 97.4 (75.1 to 124.2) | 25.7 (19.9 to 32.8) | 125 (95.1 to 165) | 45 (34.2 to 59.3) | 2.05 (-0.25 to 4.41) |
| DALYs | Haiti | 3470.8 (1490.2 to 12857.2) | 98.7 (43.4 to 353.1) | 5704.6 (2848.9 to 12531.1) | 101.3 (50.8 to 222) | 0.16 (-0.11 to 0.44) |
| DALYs | Honduras | 2725.1 (1860.4 to 3735.5) | 97.1 (66.3 to 133.2) | 2834.7 (1461.8 to 4649.8) | 66.2 (34.1 to 108.6) | -1.27 (-1.38 to -1.15) |
| DALYs | Hungary | 2791.1 (2471.3 to 3133.5) | 99.8 (88.3 to 112) | 834.2 (689.1 to 1007.8) | 44.5 (36.7 to 53.9) | -2.74 (-3.89 to -1.57) |
| DALYs | Iceland | 122.3 (100.5 to 146.5) | 145.5 (119.7 to 174.3) | 68.4 (52.5 to 86.1) | 74.5 (57.1 to 94) | -1.67 (-3.1 to -0.21) |
| DALYs | India | 230624.1 (138994.2 to 352128.9) | 55.2 (33.4 to 83.9) | 218108.9 (160423.2 to 300068.3) | 44.9 (32.8 to 62.2) | -0.65 (-0.95 to -0.35) |
| DALYs | Indonesia | 45983.1 (25298.1 to 74702.3) | 52.6 (28.8 to 86.1) | 49808.9 (32426.9 to 69908.2) | 55.4 (36 to 78.2) | 0.13 (-0.08 to 0.35) |
| DALYs | Iran (Islamic Republic of) | 41028.2 (25084.9 to 57636.3) | 127.3 (77.6 to 178.1) | 29483.6 (17387.3 to 38464.9) | 114.2 (66.7 to 150) | -0.36 (-0.64 to -0.08) |
| DALYs | Iraq | 10621.3 (6482.7 to 19205.9) | 102.3 (62.5 to 183.7) | 18820.6 (12338.6 to 28618.9) | 107.9 (70.8 to 164.4) | 0.06 (-0.18 to 0.31) |
| DALYs | Ireland | 1257.5 (1050.8 to 1487) | 96.2 (80.5 to 113.6) | 666.2 (523 to 830.4) | 50.7 (39.8 to 63.3) | -2.07 (-2.89 to -1.23) |
| DALYs | Israel | 1985.4 (1676.5 to 2336.5) | 100.3 (84.8 to 118) | 1928.5 (1522.2 to 2419.4) | 57.1 (45.1 to 71.6) | -1.7 (-2.82 to -0.57) |
| DALYs | Italy | 13607.2 (12814.1 to 14426.5) | 104.5 (98.3 to 110.9) | 5988.4 (5308.7 to 6718.2) | 57.4 (50.3 to 65) | -2.34 (-2.87 to -1.82) |
| DALYs | Jamaica | 557.9 (397.3 to 746.8) | 51.8 (36.9 to 69.4) | 374.6 (271.5 to 503) | 48.2 (34.8 to 64.7) | -0.08 (-1.29 to 1.14) |
| DALYs | Japan | 13688.2 (13049.1 to 14359.4) | 42.6 (40.6 to 44.7) | 10204.7 (9524.9 to 10903.5) | 48.3 (44.9 to 51.8) | 0.46 (-0.29 to 1.22) |
| DALYs | Jordan | 1771.2 (1180.1 to 2549) | 84.2 (56.1 to 121.3) | 3134.8 (2058.4 to 4589.1) | 65.4 (42.9 to 95.7) | -0.81 (-1.02 to -0.61) |
| DALYs | Kazakhstan | 5398.3 (4221.5 to 6817.6) | 81.1 (63.4 to 102.6) | 4061.3 (3133.5 to 5106.3) | 59.7 (46.1 to 75) | -1.06 (-1.55 to -0.57) |
| DALYs | Kenya | 5734.6 (3747.1 to 10214.2) | 39.8 (26 to 69.6) | 11781.6 (8012.9 to 18923.3) | 48.8 (33 to 79.4) | 0.68 (0.29 to 1.08) |
| DALYs | Kiribati | 6.4 (2.6 to 10.8) | 17.1 (7.1 to 28.8) | 9.4 (4 to 16.5) | 17.6 (7.4 to 30.8) | 0.02 (-0.41 to 0.46) |
| DALYs | Kuwait | 482.6 (390.5 to 591.6) | 68.8 (55.6 to 84.3) | 347.2 (258.4 to 454.1) | 31.6 (23.5 to 41.3) | -2.27 (-3.81 to -0.71) |
| DALYs | Kyrgyzstan | 830.7 (616.2 to 1113.6) | 37.7 (28.1 to 50.3) | 1955 (1503.2 to 2496.5) | 68.3 (52.6 to 87.2) | 1.97 (1.17 to 2.78) |
| DALYs | Lao People's Democratic Republic | 1186.9 (559.3 to 3073.8) | 51.4 (24.6 to 129.7) | 1631.2 (950.2 to 2578.6) | 54.9 (32 to 86.6) | 0.22 (-0.04 to 0.49) |
| DALYs | Latvia | 629.2 (518.3 to 763.4) | 84.2 (69.4 to 102.1) | 247.6 (191.5 to 314.1) | 64.4 (49.6 to 82) | -0.57 (-2.24 to 1.12) |
| DALYs | Lebanon | 823.9 (491.5 to 1291.6) | 60.8 (36.4 to 95.5) | 966.4 (543.6 to 1541) | 59.1 (32.8 to 94.7) | -0.08 (-0.51 to 0.35) |
| DALYs | Lesotho | 195.9 (123.7 to 320.9) | 22.9 (14.4 to 37.4) | 322.5 (193.6 to 517.4) | 38.8 (23.3 to 62.2) | 1.75 (1.36 to 2.15) |
| DALYs | Liberia | 286.2 (161.5 to 503.5) | 18.5 (10.5 to 32.2) | 693 (294.9 to 1090.5) | 24.7 (10.5 to 38.9) | 0.8 (-0.2 to 1.8) |
| DALYs | Libya | 2611.5 (1477.7 to 3910.7) | 112.8 (63.9 to 168.9) | 2312.5 (1327.7 to 3595.8) | 114.7 (64.6 to 179.8) | 0.03 (-0.29 to 0.35) |
| DALYs | Lithuania | 930.1 (781.7 to 1101) | 85.1 (71.5 to 100.6) | 417.2 (333.8 to 509.3) | 77.6 (62 to 94.9) | -0.09 (-1.34 to 1.17) |
| DALYs | Luxembourg | 110.2 (92.1 to 128.8) | 126 (105.4 to 147.2) | 79.6 (62.7 to 101.2) | 59.6 (46.9 to 76) | -2.18 (-3.28 to -1.07) |
| DALYs | Madagascar | 4428.8 (2862.6 to 6789.5) | 60.2 (38.8 to 92.4) | 8392.7 (4824.6 to 13374.6) | 55.8 (32.1 to 89) | -0.22 (-0.65 to 0.21) |
| DALYs | Malawi | 2044.4 (1100.6 to 3191.5) | 32.2 (17.4 to 50.1) | 3094 (1381.6 to 5248.1) | 30 (13.3 to 50.9) | -0.18 (-0.7 to 0.33) |
| DALYs | Malaysia | 3414 (1656.3 to 5351.7) | 41 (19.9 to 64.2) | 3704.6 (1956.4 to 5575) | 35.9 (18.9 to 53.9) | -0.3 (-0.95 to 0.35) |
| DALYs | Maldives | 62.9 (33.8 to 150.4) | 47.6 (26 to 111.2) | 55.9 (36.5 to 82.8) | 43.2 (28.2 to 63.9) | -0.35 (-1 to 0.3) |
| DALYs | Mali | 754.1 (304.5 to 1229.1) | 12.8 (5.3 to 20.8) | 1779.1 (611.8 to 3049.1) | 11.4 (4 to 19.4) | -0.38 (-1.28 to 0.52) |
| DALYs | Malta | 85.9 (63.7 to 105.5) | 75.5 (55.9 to 92.8) | 84.3 (61.4 to 109.8) | 100.8 (73.3 to 131.2) | 1.04 (0.16 to 1.92) |
| DALYs | Marshall Islands | 4.2 (2.4 to 6.5) | 15.4 (8.6 to 23.8) | 5.5 (3.1 to 8.5) | 23.9 (13.4 to 37.3) | 1.41 (1.09 to 1.73) |
| DALYs | Mauritania | 226.3 (142.6 to 355.3) | 18.1 (11.4 to 28.4) | 629.8 (289.1 to 997.8) | 26.5 (12.2 to 42.1) | 1.14 (0.73 to 1.55) |
| DALYs | Mauritius | 95.8 (83.3 to 111.6) | 22.3 (19.4 to 26) | 55.8 (46.4 to 66.3) | 18.6 (15.4 to 22.2) | -0.2 (-2.06 to 1.7) |
| DALYs | Mexico | 34038.3 (30866.7 to 37671.8) | 79 (71.6 to 87.4) | 24410.7 (20619 to 28963.5) | 57.1 (47.7 to 68.5) | -1.12 (-1.51 to -0.72) |
| DALYs | Micronesia (Federated States of) | 12.2 (7.1 to 19.7) | 20.9 (12.2 to 33.9) | 8.6 (4.8 to 13.4) | 20.8 (11.6 to 32.4) | -0.02 (-0.34 to 0.3) |
| DALYs | Monaco | 5.6 (3.6 to 8.3) | 118.5 (76.4 to 176.4) | 9.8 (6.3 to 13.8) | 149.3 (96.2 to 210.7) | 1.16 (0.85 to 1.47) |
| DALYs | Mongolia | 688 (404.4 to 1443.8) | 59 (34.7 to 122.9) | 1017.3 (644.2 to 1460.7) | 75.9 (48.1 to 108.6) | 0.79 (0.19 to 1.39) |
| DALYs | Montenegro | 435.7 (313.1 to 594.1) | 206.7 (148.3 to 282) | 184.6 (105.7 to 285) | 121.3 (68.2 to 188.8) | -1.62 (-2.99 to -0.24) |
| DALYs | Morocco | 5930.5 (3146.4 to 9430.1) | 47.1 (25 to 74.8) | 5229.7 (2561.3 to 8439.4) | 41 (19.9 to 66.3) | -0.47 (-0.67 to -0.26) |
| DALYs | Mozambique | 6116.2 (3657.8 to 10588.7) | 71.4 (42.5 to 124.9) | 12835.1 (6145.6 to 23227.2) | 68.7 (32.9 to 124.8) | -0.02 (-0.68 to 0.64) |
| DALYs | Myanmar | 12005 (5446.2 to 27051.3) | 63.1 (28.5 to 142.8) | 13134.6 (7979.5 to 19974.3) | 64.1 (38.8 to 97.6) | 0.07 (-0.2 to 0.34) |
| DALYs | Namibia | 254.2 (131.1 to 391.1) | 33 (17 to 50.7) | 537.8 (297 to 869.4) | 50.7 (27.9 to 81.9) | 1.37 (0.99 to 1.76) |
| DALYs | Nauru | 1.4 (0.9 to 2.1) | 25.6 (16.5 to 38.6) | 1.6 (0.9 to 2.5) | 30.8 (18 to 47.9) | 0.61 (0.42 to 0.81) |
| DALYs | Nepal | 6265 (3333.7 to 13044.4) | 57.6 (30.7 to 118) | 6427.2 (3903.3 to 10594.2) | 52.6 (31.9 to 86.8) | -0.27 (-0.53 to -0.01) |
| DALYs | Netherlands | 3094.3 (2671.6 to 3541.3) | 83.4 (72.1 to 95.3) | 2074.2 (1709.5 to 2505) | 57.1 (46.9 to 69) | -1.11 (-1.57 to -0.66) |
| DALYs | New Zealand | 1078 (916.9 to 1264.9) | 100.5 (85.5 to 117.8) | 771.7 (639.1 to 925.2) | 59.6 (49.4 to 71.5) | -1.71 (-2.89 to -0.51) |
| DALYs | Nicaragua | 1606.5 (1115.6 to 2454.1) | 68.7 (47.7 to 105.1) | 1367.8 (863.2 to 2075.2) | 53.1 (33.4 to 80.6) | -0.86 (-0.96 to -0.77) |
| DALYs | Niger | 1086.5 (637.8 to 1993.2) | 19.2 (11.3 to 34.9) | 3735.9 (1537.8 to 6221.4) | 22.1 (9.1 to 36.9) | 0.43 (-0.21 to 1.08) |
| DALYs | Nigeria | 12411.9 (7363 to 25347.7) | 22.9 (13.7 to 45.8) | 39605.2 (16915.7 to 58326.5) | 29.7 (12.8 to 43.8) | 0.84 (0.65 to 1.02) |
| DALYs | Niue | 0.3 (0.2 to 0.4) | 25.7 (16 to 38.7) | 0.7 (0.4 to 1) | 140.7 (86.3 to 211.6) | 6.35 (5.65 to 7.06) |
| DALYs | North Macedonia | 1456.6 (1022 to 2126.2) | 214.5 (150.2 to 312.9) | 637.6 (419.2 to 933.5) | 145.2 (94.3 to 213.8) | -1.27 (-1.89 to -0.64) |
| DALYs | Northern Mariana Islands | 2.9 (1.6 to 5.6) | 18.5 (10 to 35.1) | 3.9 (2.5 to 6) | 25.7 (16.3 to 39.4) | 1.37 (1.02 to 1.72) |
| DALYs | Norway | 1396.6 (1294.2 to 1503.5) | 129.4 (119.8 to 139.3) | 936.9 (845.7 to 1040.3) | 74 (66.5 to 82.6) | -1.62 (-2.97 to -0.26) |
| DALYs | Oman | 381.9 (200.1 to 609.6) | 36.9 (19.4 to 58.9) | 460.5 (256.3 to 705.5) | 30.4 (17 to 46.7) | -0.6 (-0.86 to -0.35) |
| DALYs | Pakistan | 40806.5 (23184.7 to 67809.8) | 64.7 (36.7 to 106.8) | 87674.5 (59035.4 to 130259.1) | 80 (53.9 to 118.8) | 0.68 (0.43 to 0.92) |
| DALYs | Palau | 1.1 (0.7 to 1.6) | 17.5 (11 to 26.5) | 0.7 (0.4 to 1.1) | 16.3 (9.9 to 24) | -0.26 (-0.47 to -0.05) |
| DALYs | Palestine | 1710.1 (1065 to 2855.5) | 140.3 (87.5 to 232.4) | 2860.8 (1924.7 to 4389.3) | 118.9 (80 to 182.5) | -0.45 (-1.05 to 0.15) |
| DALYs | Panama | 1393.4 (1128.4 to 1704.3) | 129.4 (104.8 to 158.3) | 987 (752.4 to 1283.4) | 65.9 (50.1 to 85.7) | -2.33 (-3.31 to -1.34) |
| DALYs | Papua New Guinea | 381.1 (154.5 to 796.7) | 17.7 (7 to 36.7) | 1134.1 (550.3 to 2019.2) | 22.9 (11 to 40.5) | 0.83 (0.23 to 1.43) |
| DALYs | Paraguay | 848.4 (588 to 1280) | 39.7 (27.5 to 60.1) | 1536.6 (920 to 2341.7) | 58.5 (34.9 to 89.5) | 1.52 (1.26 to 1.78) |
| DALYs | Peru | 15396 (10261.9 to 24035) | 144.2 (96.1 to 225) | 12788.1 (8203.5 to 19353.9) | 102.9 (65.9 to 155.9) | -1.08 (-1.36 to -0.8) |
| DALYs | Philippines | 18817.7 (11809 to 27893.4) | 58.5 (36.9 to 86.2) | 23998.1 (17421.7 to 30768.8) | 54 (39.2 to 69.3) | -0.24 (-0.52 to 0.04) |
| DALYs | Poland | 17466.5 (16642.9 to 18286.1) | 142.5 (135.7 to 149.2) | 4859.6 (4335.7 to 5413.5) | 63.2 (56.1 to 70.6) | -2.58 (-3.46 to -1.68) |
| DALYs | Portugal | 4153.5 (3575.6 to 4805.2) | 147.2 (127.2 to 170) | 1287.8 (1040.4 to 1594) | 69.4 (56.1 to 86.1) | -2.55 (-3.45 to -1.64) |
| DALYs | Puerto Rico | 660.6 (516.7 to 832.2) | 50.2 (39.4 to 63) | 213 (164.3 to 269.4) | 33 (25.6 to 41.6) | -1.23 (-2.05 to -0.39) |
| DALYs | Qatar | 63 (39.9 to 98.2) | 41.9 (26.5 to 65) | 185.4 (112.6 to 298.2) | 31.9 (19.4 to 50.9) | -0.91 (-1.28 to -0.54) |
| DALYs | Republic of Korea | 15770.3 (10302.5 to 20135.7) | 99.8 (65.2 to 127.6) | 4817.4 (2862.7 to 6506.4) | 59.6 (35 to 81.3) | -1.69 (-2.15 to -1.23) |
| DALYs | Republic of Moldova | 2572.4 (2173.9 to 3022.9) | 161.9 (136.8 to 190.2) | 648.3 (516 to 805.9) | 96.5 (76.2 to 121) | -1.81 (-2.62 to -0.98) |
| DALYs | Romania | 11592.2 (10119.6 to 13094.6) | 162 (141.5 to 182.7) | 3244.6 (2638.2 to 3914.9) | 81.1 (65.9 to 98) | -1.86 (-2.14 to -1.58) |
| DALYs | Russian Federation | 55857.1 (53747.8 to 58159.8) | 124.3 (119.6 to 129.5) | 24298.5 (22197.2 to 26220.5) | 72.2 (65.7 to 78.1) | -1.89 (-2.84 to -0.93) |
| DALYs | Rwanda | 3552.1 (2169.4 to 5587.4) | 78.9 (48.2 to 123.8) | 4530.7 (2575.9 to 6996.4) | 70.5 (40.1 to 108.9) | -0.39 (-1.6 to 0.84) |
| DALYs | Saint Kitts and Nevis | 10.4 (8 to 13.3) | 57.1 (43.8 to 73.1) | 9.8 (7.5 to 12.6) | 72.1 (54.5 to 93.5) | 0.85 (0.45 to 1.25) |
| DALYs | Saint Lucia | 32.8 (24.5 to 43.1) | 49.4 (37 to 64.9) | 26.8 (20.1 to 34.7) | 65.9 (49.2 to 85.9) | 0.91 (0.34 to 1.49) |
| DALYs | Saint Vincent and the Grenadines | 30.4 (23.8 to 38.4) | 57.4 (44.8 to 72.5) | 26 (20.2 to 32.6) | 77 (59.3 to 96.9) | 1.06 (-0.92 to 3.09) |
| DALYs | Samoa | 41.4 (24.6 to 65.6) | 45.3 (26.9 to 71.7) | 45.1 (24.6 to 72.3) | 44.4 (24.4 to 71.1) | -0.04 (-0.4 to 0.33) |
| DALYs | San Marino | 10.4 (7 to 15.3) | 189.2 (125.9 to 283.7) | 7 (4.4 to 10.4) | 118.9 (73.3 to 177.3) | -1.53 (-1.79 to -1.27) |
| DALYs | Sao Tome and Principe | 13.4 (7.7 to 20) | 18.2 (10.4 to 27.2) | 11.1 (4.8 to 20.3) | 11.2 (4.8 to 20.6) | -1.47 (-3.08 to 0.17) |
| DALYs | Saudi Arabia | 5014.8 (3019.6 to 7866.6) | 59.8 (36.1 to 93.9) | 2971.3 (1742 to 5085.1) | 29 (17 to 49.9) | -2.31 (-2.42 to -2.19) |
| DALYs | Senegal | 1104.6 (758.8 to 1662.7) | 22.1 (15.2 to 33.3) | 2563 (1246.4 to 3916.7) | 31.2 (15.2 to 47.7) | 1.02 (0.17 to 1.87) |
| DALYs | Serbia | 5228.7 (3341.8 to 7935.8) | 185.4 (117.6 to 282.8) | 1348.6 (881.8 to 2071.8) | 72.3 (47.2 to 111.2) | -2.89 (-3.37 to -2.41) |
| DALYs | Seychelles | 18.7 (12.8 to 27.1) | 59.9 (40.9 to 87) | 10.7 (6.2 to 16.2) | 35.6 (20.7 to 53.9) | -1.85 (-3.83 to 0.17) |
| DALYs | Sierra Leone | 506.3 (302.7 to 830.1) | 19.6 (11.8 to 31.9) | 1398.8 (617 to 2181.2) | 29.6 (13.1 to 46.3) | 1.4 (0.39 to 2.41) |
| DALYs | Singapore | 400.8 (328.9 to 487.6) | 45.4 (37.3 to 55.2) | 519.5 (417.4 to 634.5) | 50.4 (40.5 to 61.6) | 0.01 (-1.92 to 1.99) |
| DALYs | Slovakia | 1667.5 (1224.2 to 2302.9) | 96.2 (70.6 to 132.7) | 898.4 (597.1 to 1284.5) | 80.8 (53.7 to 115.5) | -0.59 (-1.02 to -0.17) |
| DALYs | Slovenia | 384.7 (321.3 to 455.4) | 69.8 (58.4 to 82.5) | 144.6 (112.5 to 185.5) | 35.4 (27.5 to 45.4) | -2.4 (-3.55 to -1.23) |
| DALYs | Solomon Islands | 29.8 (11.4 to 51.8) | 15.3 (5.8 to 26.6) | 70.7 (32.6 to 112.4) | 21.4 (9.9 to 34) | 1.01 (0.23 to 1.8) |
| DALYs | Somalia | 2079.2 (1027.5 to 4005) | 40.1 (19.8 to 76.4) | 4486.1 (1912.5 to 7800.6) | 33.5 (14.4 to 58.2) | -0.43 (-0.97 to 0.1) |
| DALYs | South Africa | 5327.1 (3904.5 to 8285.4) | 30.3 (22.2 to 47.1) | 6745.3 (5041.6 to 9289.5) | 34.1 (25.4 to 47) | 0.4 (0.08 to 0.72) |
| DALYs | South Sudan | 2069.5 (1203.4 to 3640.3) | 58.2 (33.9 to 101.9) | 3335.8 (1964.2 to 5335.5) | 60 (35.3 to 95.9) | 0.22 (-0.4 to 0.84) |
| DALYs | Spain | 10130.1 (8917 to 11433.7) | 94.3 (83.2 to 106.3) | 6529.9 (5407.3 to 7719.6) | 74.3 (61.5 to 87.8) | -1.01 (-1.13 to -0.89) |
| DALYs | Sri Lanka | 3262 (2227 to 4771.2) | 44.8 (30.6 to 65.6) | 2805.3 (1692.4 to 4333.8) | 40.2 (24.2 to 62.3) | -0.46 (-0.85 to -0.06) |
| DALYs | Sudan | 11835.9 (4870.1 to 33199.2) | 102.6 (42.8 to 279.8) | 23401 (12959.4 to 37518.2) | 109.7 (60.7 to 176) | 0.22 (0.06 to 0.37) |
| DALYs | Suriname | 206.1 (117.9 to 301.8) | 122 (69.9 to 178.7) | 259.9 (164.7 to 378.6) | 140 (88.7 to 204.1) | 0.42 (-0.27 to 1.11) |
| DALYs | Sweden | 2141.8 (1857.4 to 2460) | 103.4 (89.7 to 118.6) | 1433.4 (1197 to 1720.1) | 59.1 (49.4 to 71.1) | -1.79 (-2.94 to -0.63) |
| DALYs | Switzerland | 1147.6 (977.4 to 1354.3) | 73.8 (63 to 87) | 963.4 (776.4 to 1188.3) | 54.9 (44.2 to 67.7) | -1.06 (-2.01 to -0.09) |
| DALYs | Syrian Arab Republic | 9895.5 (6248.6 to 16275.5) | 133.1 (84.6 to 217.4) | 5531.8 (3631.1 to 8145.1) | 98.5 (63.9 to 148.2) | -0.94 (-1.26 to -0.61) |
| DALYs | Taiwan (Province of China) | 4825.7 (4229.4 to 5503.2) | 66.9 (58.7 to 76.2) | 1745.3 (1405.8 to 2146.7) | 43.1 (34.7 to 53) | -1.33 (-2.73 to 0.1) |
| DALYs | Tajikistan | 6127.6 (3360.8 to 10991.3) | 201 (111.9 to 356.6) | 9235 (5563.9 to 14771.2) | 200.9 (121.2 to 321.2) | -0.03 (-0.26 to 0.21) |
| DALYs | Thailand | 16420.2 (9216.5 to 23715.2) | 73.3 (40.8 to 106.3) | 6954.8 (4537.7 to 9668.1) | 50.9 (33.2 to 70.4) | -1.15 (-1.47 to -0.83) |
| DALYs | Timor-Leste | 204.3 (91.4 to 534.8) | 48 (21.9 to 118.9) | 336.2 (200.2 to 537.7) | 49.5 (29.5 to 79.2) | 0.08 (-0.39 to 0.55) |
| DALYs | Togo | 425.1 (281 to 609.2) | 18.1 (11.9 to 25.9) | 1060.9 (478.7 to 1674.4) | 25 (11.3 to 39.5) | 1.05 (0.57 to 1.54) |
| DALYs | Tokelau | 0.2 (0.1 to 0.3) | 24.2 (13.6 to 37.3) | 0.9 (0.5 to 1.4) | 203.3 (101.6 to 315.3) | 8.44 (7.6 to 9.29) |
| DALYs | Tonga | 14.5 (7.7 to 25.6) | 27.2 (14.5 to 48.1) | 15.8 (8.4 to 27.1) | 31.7 (16.9 to 54.4) | 0.49 (0.24 to 0.75) |
| DALYs | Trinidad and Tobago | 256.1 (199.4 to 313.5) | 48.8 (38.1 to 59.8) | 166.1 (125.4 to 214.6) | 45.9 (34.5 to 59.7) | -0.01 (-1.3 to 1.29) |
| DALYs | Tunisia | 3498.3 (1932.7 to 5380.5) | 87.7 (48.4 to 134.9) | 2080.7 (1103.6 to 3204.4) | 58.1 (30.7 to 89.6) | -1.3 (-1.5 to -1.11) |
| DALYs | Turkey | 45499.5 (27227.4 to 72778.2) | 172.2 (102.8 to 275.6) | 23905.5 (16046.1 to 33566.3) | 97.6 (65.3 to 137.3) | -1.86 (-2.09 to -1.63) |
| DALYs | Turkmenistan | 1028.6 (812.2 to 1251.7) | 52.2 (41.3 to 63.4) | 3355.3 (2570.3 to 4251.2) | 171 (131 to 216.7) | 3.94 (3.09 to 4.79) |
| DALYs | Tuvalu | 0.9 (0.5 to 1.8) | 20.8 (11.2 to 40.5) | 1 (0.6 to 1.6) | 21.1 (12 to 32.7) | 0.08 (-0.11 to 0.26) |
| DALYs | Uganda | 8041.7 (4289.7 to 12853.6) | 67.8 (36.4 to 108) | 21812.9 (11167.1 to 35467) | 84.5 (43.5 to 137.1) | 0.7 (-0.1 to 1.51) |
| DALYs | Ukraine | 16149.3 (11614.9 to 21593.8) | 108.3 (77.8 to 145) | 8557.4 (6470.5 to 10802.9) | 105.7 (79.2 to 133.9) | 0.04 (-0.59 to 0.67) |
| DALYs | United Arab Emirates | 455.1 (241.8 to 913.9) | 66.9 (36.1 to 131.7) | 794.8 (482.9 to 1329.1) | 47.1 (28.7 to 78.8) | -1.11 (-2.01 to -0.2) |
| DALYs | United Kingdom | 13024.3 (12550.8 to 13499.3) | 89.2 (85.9 to 92.5) | 8106.7 (7526.9 to 8613.1) | 51.7 (47.9 to 55.1) | -1.55 (-2 to -1.11) |
| DALYs | United Republic of Tanzania | 12564 (7529.6 to 18433.2) | 77.1 (46.2 to 113.6) | 26741.7 (15371.2 to 41188.9) | 84.3 (48.6 to 129.8) | 0.24 (0 to 0.48) |
| DALYs | United States of America | 59715 (58007.2 to 61457.1) | 81.4 (79.1 to 83.8) | 48339 (44920.9 to 52026.1) | 59.9 (55.5 to 64.6) | -0.97 (-1.39 to -0.55) |
| DALYs | United States Virgin Islands | 19.9 (12.2 to 34.3) | 47.7 (29.3 to 82.2) | 6.2 (3 to 11.4) | 35.3 (17.3 to 65.1) | -1.1 (-1.77 to -0.42) |
| DALYs | Uruguay | 804.4 (655.5 to 984.7) | 75.6 (61.7 to 92.4) | 560.4 (426.6 to 726.5) | 61.5 (46.7 to 79.9) | -0.97 (-1.6 to -0.35) |
| DALYs | Uzbekistan | 12692.5 (8483.2 to 17802.6) | 115.2 (77.4 to 161.3) | 19297.8 (14999 to 24420) | 149.6 (116.5 to 188.9) | 0.83 (0.44 to 1.23) |
| DALYs | Vanuatu | 11.4 (5.5 to 19.1) | 13.7 (6.6 to 22.9) | 26.9 (13.9 to 42.1) | 18.2 (9.5 to 28.5) | 0.99 (-0.69 to 2.7) |
| DALYs | Venezuela (Bolivarian Republic of) | 2642.5 (2340.6 to 2982.6) | 29 (25.7 to 32.8) | 4805 (3496.3 to 6350.3) | 54.6 (39.6 to 72.4) | 2.12 (1.22 to 3.03) |
| DALYs | Viet Nam | 12198.2 (6594.4 to 18723.5) | 35.8 (19.4 to 55) | 10755.3 (6096.6 to 16499.2) | 33.9 (19.2 to 52) | -0.19 (-0.51 to 0.13) |
| DALYs | Yemen | 5821.1 (2417.3 to 15154.7) | 63.8 (26.1 to 162.3) | 13475.7 (7349.4 to 21053.4) | 76.4 (41.5 to 119.7) | 0.56 (0.34 to 0.77) |
| DALYs | Zambia | 3967.5 (2430.6 to 5913.2) | 77.1 (47.1 to 114.9) | 7859.6 (4326.8 to 12223) | 73.9 (40.7 to 115) | -0.06 (-0.35 to 0.23) |
| DALYs | Zimbabwe | 2263.2 (1286.4 to 3302.6) | 36.9 (21 to 53.9) | 5604.4 (3298.2 to 8513.2) | 69.8 (41.1 to 106) | 1.69 (0.57 to 2.82) |

ASR, age-standardized rate; AAPC, average annual percentage changes; DALYs, disability adjusted life years.

## Supplementary Table 3. Decomposition analysis of DALYs burden for CABCs in 2021 by location and gender

| Location | Sex | Cause | Measure | Overall difference | Population size | Population age | Prevalence | Case fatality and disease severity | Percent change of Population size | Percent change of Population age | Percent change of Prevalence | Percent change of Case fatality and disease severity |
| --- | --- | --- | --- | --- | --- | --- | --- | --- | --- | --- | --- | --- |
| Global | Both | Brain and central nervous system cancer | DALYs | -498553.0332 | 298136.1592 | -27581.16299 | 283801.1534 | -1052909.183 | -59.8002899 | 5.532242541 | -56.92496776 | 211.1930151 |
| Global | Male | Brain and central nervous system cancer | DALYs | -339695.2577 | 171377.8358 | -15186.2603 | 119274.2609 | -615161.0941 | -50.4504646 | 4.470554107 | -35.112136 | 181.0920465 |
| Global | Female | Brain and central nervous system cancer | DALYs | -158857.7755 | 127587.4562 | -12457.65132 | 159941.038 | -433928.6184 | -80.31552487 | 7.842015463 | -100.6819072 | 273.1554166 |
| High SDI | Both | Brain and central nervous system cancer | DALYs | -73230.18011 | -13150.72078 | 207.9002513 | 34599.47836 | -94886.83794 | 17.9580615 | -0.283899686 | -47.24756693 | 129.5734051 |
| High SDI | Female | Brain and central nervous system cancer | DALYs | -32305.5969 | -5998.246453 | 142.9090874 | 14181.77116 | -40632.03069 | 18.56720515 | -0.442366342 | -43.89880552 | 125.7739667 |
| High SDI | Male | Brain and central nervous system cancer | DALYs | -40924.58321 | -7123.889139 | 73.61786551 | 20445.40361 | -54319.71554 | 17.40735905 | -0.179886659 | -49.95873386 | 132.7312615 |
| High-middle SDI | Both | Brain and central nervous system cancer | DALYs | -294463.0364 | -77958.01617 | 178.7330168 | 173748.683 | -390432.4363 | 26.4746357 | -0.060697947 | -59.00526094 | 132.5913232 |
| High-middle SDI | Female | Brain and central nervous system cancer | DALYs | -125381.7187 | -36995.19646 | 180.9785966 | 80652.08775 | -169219.5886 | 29.50605306 | -0.144342093 | -64.32523702 | 134.963526 |
| High-middle SDI | Male | Brain and central nervous system cancer | DALYs | -169081.3177 | -40139.19497 | 14.10150837 | 91202.34695 | -220158.5712 | 23.73958017 | -0.008340075 | -53.93993151 | 130.2086914 |
| Middle SDI | Both | Brain and central nervous system cancer | DALYs | -349205.0154 | -14815.70814 | -7686.515918 | 244528.9271 | -571231.7185 | 4.242696262 | 2.201147056 | -70.02446021 | 163.5806169 |
| Middle SDI | Male | Brain and central nervous system cancer | DALYs | -207239.1257 | -2832.028122 | -4313.791386 | 109633.0854 | -309726.3916 | 1.366550893 | 2.081552589 | -52.90173131 | 149.4536278 |
| Middle SDI | Female | Brain and central nervous system cancer | DALYs | -141965.8897 | -11236.7113 | -3359.59626 | 131200.4925 | -258570.0747 | 7.91507828 | 2.366481319 | -92.41691279 | 182.1353532 |
| Low-middle SDI | Both | Brain and central nervous system cancer | DALYs | 78442.33925 | 108849.0113 | -15484.79878 | 140860.6331 | -155782.5064 | 138.7630868 | -19.74035824 | 179.5721984 | -198.594927 |
| Low-middle SDI | Male | Brain and central nervous system cancer | DALYs | 19807.77309 | 60988.13389 | -9750.592365 | 65377.93063 | -96807.69907 | 307.9000028 | -49.22609079 | 330.061993 | -488.735905 |
| Low-middle SDI | Female | Brain and central nervous system cancer | DALYs | 58634.56616 | 47936.88269 | -5791.07475 | 75520.89007 | -59032.13185 | 81.75532937 | -9.876554273 | 128.7992647 | -100.6780398 |
| Low SDI | Male | Brain and central nervous system cancer | DALYs | 58029.73233 | 80546.94534 | -5863.208803 | 5096.89577 | -21750.89998 | 138.8028897 | -10.10380122 | 8.783248803 | -37.48233725 |
| Low SDI | Both | Brain and central nervous system cancer | DALYs | 140352.0599 | 152694.0448 | -11155.60473 | 37436.05801 | -38622.43814 | 108.7935901 | -7.948301391 | 26.67296656 | -27.51825528 |
| Low SDI | Female | Brain and central nervous system cancer | DALYs | 82322.32758 | 72081.01365 | -5269.497726 | 32352.26329 | -16841.45163 | 87.5594942 | -6.401055316 | 39.2995002 | -20.45793909 |

SDI, socio-demographic index; DALYs, disability adjusted life years.
